# Glucuronidated Hydroxyphenylacetic and Hydroxyphenylpropanoic Acids as Standards for Bioavailability Studies with Flavonoids

**DOI:** 10.1021/acsomega.5c09380

**Published:** 2025-12-25

**Authors:** Viola Janouchová, Martina Hurtová, Hana Kočová Vlčková, Zuzana Lomozová, Jana Pourová, Lucie Nováková, Lucie Petrásková, Helena Pelantová, Josef Cvačka, Kateřina Valentová

**Affiliations:** † 48311 Institute of Microbiology of the Czech Academy of Sciences, Vídeňská 1083, Prague CZ-142 00, Czech Republic; ‡ Faculty of Pharmacy, 37740 Charles University, Akademika Heyrovského 1203, Hradec Králové CZ-50005, Czech Republic; § Institute of Organic Chemistry and Biochemistry of the Czech Academy of Sciences, Flemingovo náměstí 542/2, Prague CZ-160 00, Czech Republic

## Abstract

Glucuronidation is a major phase II biotransformation of (poly)­phenols leading to potentially bioactive metabolites. Due to the limited availability of authentic standards, in this work, we have focused on the glucuronidation of a series of mono- and dihydroxyphenolic acids. Their reactivity with two glucuronidation reagents, 2,3,4-triaceto-1-bromo-α-d-glucuronic acid methyl ester and Schmidt imidate, was investigated. The use of Schmidt imidate led to the successful synthesis of six target glucuronides in moderate to excellent yields. Subsequent deprotection of these compounds afforded the final glucuronides of 2-hydroxyphenylacetic, 3-hydroxyphenylacetic, 4-hydroxyphenylacetic, 3,4-dihydroxyphenylacetic, 3-(4-hydroxyphenyl)­propionic, and 3-(3,4-dihydroxyphenyl)­propionic acid. These compounds, which are plausible polyphenolic metabolites, were fully characterized and used in a pilot metabolic study in rats after administration of a hawthorn berry extract (475 mg/kg). UHPLC-HRMS analysis identified two of the glucuronides, namely 4-hydroxyphenylacetic acid glucuronide and 3-hydroxyphenylacetic acid glucuronide in rat plasma, confirming their *in vivo* formation.

## Graphical abstract

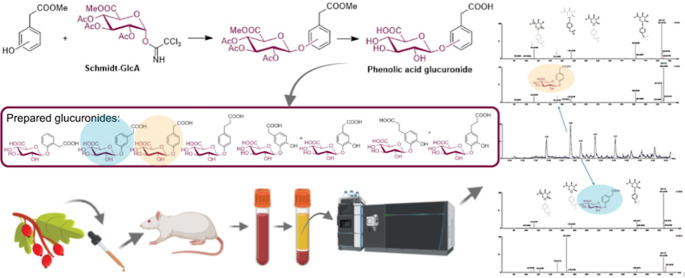

## 1 Introduction

Phenolic acids, characterized by a phenolic ring with one or more hydroxyl groups, are common dietary constituents, but also microbial metabolites of complex food polyphenols produced in the gut. Hydroxyphenylacetic and hydroxyphenylpropanoic acids are typical examples of such metabolites.^1^ These compounds are abundant in foods and exhibit diverse biological activities.^2,3^ In the human body, they are absorbed from the gut and subsequently conjugated with sulfate or glucuronide in enterocytes and hepatocytes. Although a large proportion of these conjugates is excreted, they remain in the bloodstream for some time and may exert biological activity.^4^ Low-molecular-weight phenolic metabolites, including some conjugates, have been found to cross the blood–brain barrier and are therefore able to exert direct neuroprotective effects.^5^ Fully structurally characterized compounds are required for the evaluation of biological activity and metabolic studies with foods rich in phenolic acids or their precursors. Only some of these compounds are commercially available but expensive. Therefore, we aimed to develop a synthetic method for the preparation of these derivatives. Recently, we have reported the preparation of sulfated hydroxyphenylacetic and hydroxyphenylpropanoic acids,^6^ and in this work, we have focused on the preparation of conjugates with glucuronic acid.

Glucuronidation is a phase II metabolic pathway responsible for the conjugation and subsequent elimination of various endogenous and exogenous compounds from the body. This usually involves the enzymatic transfer of a glucuronic acid moiety from the cofactor UDP-glucuronic acid to the hydroxyl groups, catalyzed by UDP-glucuronosyltransferases (UGTs).^7,8^ For synthetic purposes, this method is only suitable for the preparation of small amounts of analytical standards. There are two main reasons for this: (i) UGTs are transmembrane proteins^8^ that can hardly be expressed recombinantly (e.g., as microsomes from baculovirus-transfected insect cells expressing the recombinant human UGTs) and (ii) UDP-glucuronic acid is an expensive glucuronate donor. In addition, isolation and purification of the glucuronidated phenolic product from a system containing membranes is very challenging. Other possibilities include microbial biotransformation.^9^

On the other hand, chemical synthesis offers several methods for the production of glucuronides. Perhaps the best known is the reaction of 2,3,4-triaceto-1-bromo-α-d-glucuronic acid methyl ester (perAc-GlcA-Br) with phenols under basic conditions, e.g., the Koenigs-Knorr glycosidation using silver oxide^10,11^ or the modified Koenigs-Knorr glycosidation employing silver carbonate.^12^ The disadvantage of Koenigs–Knorr glycosidation is the use of heavy metals (besides Ag also Cd and Hg), which can form complexes with phenols. During synthesis, the hydroxy-groups of the sugar moiety are protected by acetates, which can be removed by hydrolysis or by Zemplén deacetylation.^13^ Electron-rich phenols can also be glucuronidated using a reaction with tetraacetyl-β-d-glucuronic acid methyl ester or with 2,3,4-tri-*O*-acetyl-1-*O*-(trichloroacetimidoyl)-α-d-glucuronic acid (Schmidt imidate; Schmidt-GlcA), in both cases catalyzed by Lewis acid (Scheme 1). The method with the glucuronate tetraacetate works only for low acidity phenols (p*K*_a_ ∼ 10.0 or higher).^10,14–16^

**Scheme 1:**
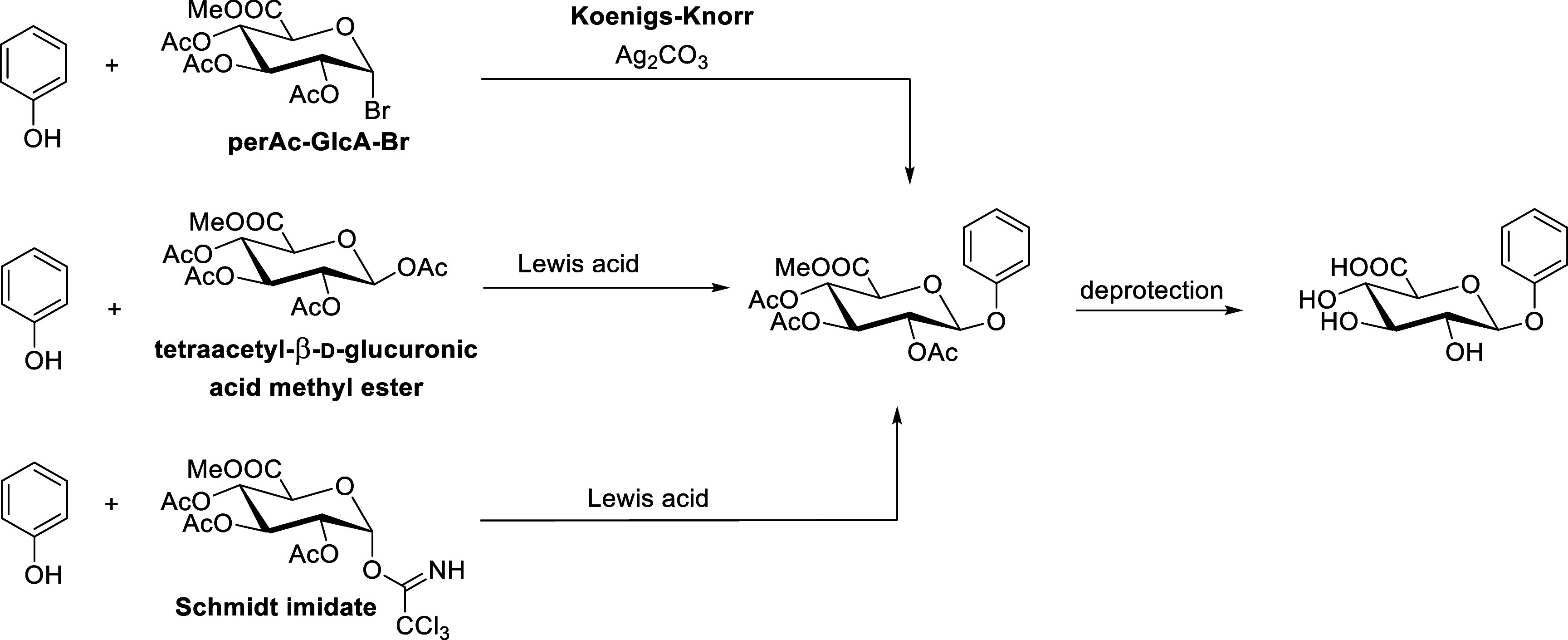
Glucuronidation of Phenols—Common Methods

There are several studies dealing with the identification of phenolic acid metabolites in plasma and urine after the consumption of polyphenol-rich substances, using UHPLC-MS/MS and UHPLC-HRMS to quantify and characterize the metabolites. Glucuronides of dihydrocaffeic acid, dihydroferrulic acid, and dihydrocoumaric acid were found in plasma and urine after coffee consumption.^17^ Isoferrulic acid 3′-*O*-glucuronide was found in plasma after consumption of flavonoid-rich supplements.^18^

In this work, we have focused on the glucuronidation of a series of mono- and dihydroxyphenolic acids (Figure 1). Using a multistep synthesis, we have synthesized a library of glucuronides in the form of free acids. The obtained glucuronides were subsequently used as fully characterized standards for a pilot metabolic study in rats after intake of a hawthorn berry extract. Hawthorn extract is rich in polyphenols such as phenolic acids, flavonols, flavanols, anthocyanins, procyanidins, and lignans. Extracts of hawthorn berries, flowers, and leaves are used for their antimicrobial, anti-inflammatory, anticancer and antiatherosclerotic effects.^19^

**Figure 1:**
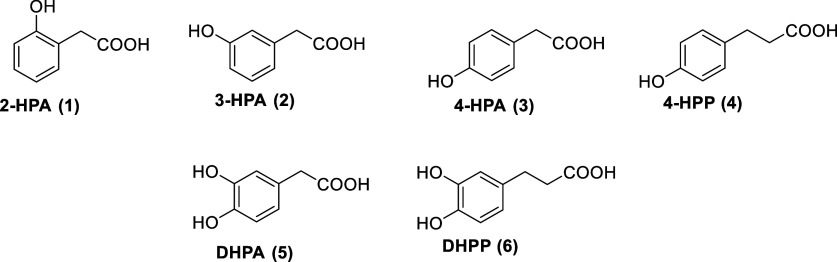
Phenolic acids selected for glucuronidation: 2-hydroxyphenylacetic acid (2-HPA, **1**), 3-hydroxyphenylacetic acid (3-HPA, **2**), 4-hydroxyphenylacetic acid (4-HPA, **3**), 3-(4-hydroxyphenyl)­propionic acid (4-HPP, **4**), 3,4-dihydroxyphenylacetic acid (DHPA, **5**), and 3-(3,4-dihydroxyphenyl)­propionic acid (DHPP, **6**).

## 2 Materials and Methods

### 2.1 Materials and Chemical Reagents

Aluminum plates coated with silica gel for analytical TLC (Silica Gel 60 F254) were from Merck (Darmstadt, Germany). 2-Hydroxyphenylacetic acid (2-HPA, **1**), 3-hydroxyphenylacetic acid (3-HPA, **2**), 4-hydroxyphenylacetic acid (4-HPA, **3**), and 3-(4-hydroxyphenyl)­propionic acid (4-HPP, **4**) were purchased from Merck (Darmstadt, Germany); 3,4-dihydroxyphenylacetic acid (DHPA, **5**) was purchased from Apollo Scientific (Bradbury, England) and 3-(3,4-hydroxyphenyl)­propionic acid (DHPP, **6**) from Thermo Fisher (Waltham, MA, USA). Acetone, cyclohexane, *N*,*N*-dimethylformamide, ethyl acetate, methanol, and toluene were purchased from VWR International (Stříbrná Skalice, Czech Republic), dichloromethane from Acros-Organics (Morris Plains, NJ, USA), methyl 1,2,3,4-tetra-*O*-acetyl-β-d-glucopyranosyluronate from Apollo Scientific (Bradbury, England), benzylamine from Sigma-Aldrich (Merck, Darmstadt, Germany), 2,2,2-trichloroacetonitrile from Thermo Fisher (Waltham, MA, USA), potassium carbonate from VWR International (Stříbrná Skalice, Czech Republic), molecular sieves 4 Å from Bld Pharmatech (Shanghai, China), potassium hydroxide from Lach-Ner (Neratovice, Czech Republic), boron trifluoride-diethyl ether complex from Merck (Darmstadt, Germany), Dowex 50WX8 from Sigma-Aldrich (Merck, Darmstadt, Germany) and Sephadex LH-20 from Cytiva Sweden AB (Uppsala, Sweden). For the metabolic study, a hawthorn extract (Hawthorn Berry, containing 2000 mg of extract containing mainly vitexin, orientin, hyperoside, and rutin in 30 mL of glycerin and water) was purchased from Naturés Answer (New York, USA). Quercetin, urethane, and DMSO were acquired from Sigma-Aldrich (St. Louis, USA). Saline was obtained from B. Braun (Melsungen, Germany) and heparin was sourced from Zentiva (Prague, Czech Republic). LC–MS grade water, acetonitrile (CH_3_CN) and methanol (MeOH) were boughtfrom Fisher Scientific (Loughborough, UK). Acetic acid and formic acid in LC–MS grade were supplied by VWR International S.A.S. (Fontenay-sous-Bois, France).

### 2.2 HPLC Analyses

All analytical HPLC analyses of the prepared glucuronides were carried out on a LCMS-2020 Prominence chromatograph (Shimadzu, Kyoto, Japan), equipped with a DGU-20 A_3_ mobile phase degasser, a SIL-20AC cooling autosampler, two LC-20AD high-pressure pumps, a CTO-10AS column oven and a SPD-M20A diode array detector. The data were acquired at a rate of 40 Hz using Shimadzu Solution software (version 5.75 SP2). Separation was achieved on a monolithic column (Chromolith Performance RP-18e, Merck, Darmstadt, Germany, 100 × 3 mm i.d.) fitted with a guard column (Merck, 5 × 4.6 mm). The mobile phases consisted of A: CH_3_CN/H_2_O/HCOOH (5:95:0.1) and B: CH_3_CN/H_2_O/HCOOH (80:20:0.1). The gradient program was as follows: 0–2 min, 0% B; 2–7 min, 0–90% B; 7–8 min, 90% B; 8–11 min, 90–0% B; 11–14 min, 0% B for re-equilibration. The flow rate was 1.2 mL/min, and the column temperature was kept at 25 °C.

### 2.3 HRMS Analyses

HRMS spectra were acquired using a hybrid mass spectrometer LTQ Orbitrap XL (Thermo Fisher Scientific, Waltham, MA, USA) assembled with an electrospray ion source. The mobile phase consisted of MeOH/H_2_O (4:1, *v*/*v*) using a flow rate of 100 μL/min. The samples were injected into the mobile phase flow dissolved in MeOH or MeOH/H_2_O using a 5 μL injection loop. In the negative ion mode, the spray, capillary, and tube lens voltage, and capillary temperature were set to 5.0 kV, −25 V, −125 V, and 275 °C, respectively. For the positive mode, the spray, capillary and tube lens voltage, and capillary temperature were adjusted to 5.0 kV, 9 V, 150 V, and 275 °C, respectively. The spectra were acquired with a resolution of 100,000.

### 2.4 NMR Analyses

The NMR analyses were carried out on spectrometers Bruker AVANCE III 700 and 600 MHz spectrometers (Bruker BioSpin, Rheinstetten, Germany) in CDCl_3_ at 20 °C (protected glucuronides) or in D_2_O at 30 °C (glucuronides—free acids). Spectra were referenced using the solvent residual signals (CDCl_3_: δ_H_ 7.263 ppm, δ_C_ 77.01 ppm; D_2_O: δ_H_ 4.732 ppm); ^13^C NMR spectra in D_2_O were referenced to the signal of acetone (δ_C_ 30.50 ppm). Structure elucidation was based on information extracted from ^1^H NMR, ^13^C NMR, COSY, ^1^H–^13^C HSQC, and ^1^H–^13^C HMBC experiments, acquired using the manufacturer’s software TopSpin 3.5. The strong overlap of glucose proton signals precluded the extraction of coupling constants except for *J*_H1, H2_ corresponding to the β-anomer. The carbon signals of glucose were assigned using their correlations observed in the HMBC spectra.

### 2.5 Electronic Circular Dichroism and Optical Rotation

Electronic circular dichroism (ECD) spectra were obtained using Jasco 815 spectrometer (Tokyo, Japan) in the spectral range 190–350 nm with a 0.01 cm cylindrical quartz cell, and in the near-UV range (250–280 nm) with a 0.5 cm quartz cell, using the following experimental setup: step resolution of 0.1 nm, scanning speed of 10 nm/min, response time of 8 s, spectral bandwidth of 1 nm and 3 accumulations. All samples were dissolved in acetonitrile; the solution with the same concentration as that used for optical rotation measurements was used to obtain CD spectra in the near-UV region. For all spectral regions, the samples were diluted 5-fold. CD spectra of samples **3c**, **4c**, **5c** + **5c′**, and **6c** + **6c′** were expressed as differential molar extinction (Δε) and molar extinction (ε) for absorption spectra, respectively, after baseline correction. Due to dissolution problems with sample **1c**, its CD spectrum is expressed only in differential absorption and absorption, respectively, as the sample was measured in a saturated solution of unknown concentration, in contrast to sample **2c**, which was insoluble in the solvent used.

Optical rotation (OR) was measured on an AUTOPOL VI polarimeter (Rudolph Research Analytical, USA). OR could not be obtained for samples **1c** and **2c** due to the problems with their solubility.

### 2.6 Preparation of Esters

The respective phenolic acid (300 mg) was dissolved in methanol (6 mL) and a catalytic amount of H_2_SO_4_ was added. The mixture was refluxed for 6 h or until the reaction was completed under TLC control. After evaporation of the solvent under vacuum, 5 mL of water was added, the mixture was extracted with EtOAc (3 × 5 mL), and the pooled organic layers were dried over Na_2_SO_4_ and evaporated. The residuum was purified by column chromatography (cyclohexane/EtOAc 2:1) affording the target ester.

#### 2.6.1 Methyl 2-Hydroxyphenylacetate (2-HPA-Me, **1a**)

Starting from 2-hydroxyphenylacetic acid (300 mg), methyl 2-hydroxyphenylacetate was obtained as a white powder (322 mg, 98%). ^1^H NMR (400 MHz, CDCl_3_): δ 7.37 (br s, 1H, OH), 7.20 (ddd, 1H, *J* = 8.1, 7.4, 1.7 Hz, C_Ar_H), 7.10 (dd, 1H, *J* = 7.5, 1.7 Hz, C_Ar_H), 6.95 (dd, 1H, *J* = 8.1, 1.3 Hz, C_Ar_H), 6.89 (ddd, 1H, *J* = 7.5, 7.4, 1.3 Hz, CArH), 3.76 (s, 3H, CH_3_), 3.69 (s, 2H, CH_2_) ppm. NMR data match the data reported in the literature.^20^

#### 2.6.2 Methyl 3-Hydroxyphenylacetate (3-HPA-Me, **2a**)

Starting from 3-hydroxyphenylacetic acid (300 mg), methyl 3-hydroxyphenylacetate was obtained as a colorless oil (327 mg, quant.). ^1^H NMR (400 MHz, CDCl_3_): δ 7.19 (dd, 1H, *J* = 8.1, 7.6 Hz, C_Ar_H), 6.83 (ddd, 1H, *J* = 7.6, 1.6, 0.9 Hz, C_Ar_H), 6.77 (dd, *J* = 2.6, 1.6 Hz, 1H, C_Ar_H), 6.75 (ddd, 1H, *J* = 8.1, 2.6, 1.0 Hz, C_Ar_H), 5.52 (br s, 1H, OH), 3.71 (s, 3H, CH_3_), 3.59 (s, 2H, CH_2_) ppm. NMR data match the data reported in the literature.^21^

#### 2.6.3 Methyl 4-Hydroxyphenylacetate (4-HPA-Me, **3a**)

Starting from 4-hydroxyphenylacetic acid (300 mg), methyl 4-hydroxyphenylacetate was obtained as a colorless oil (312 mg, 95%). ^1^H NMR (400 MHz, CDCl_3_): δ 7.14 (m, 2H, Σ*J* = 8.5 Hz, C_Ar_H), 6.77 (m, 2H, Σ*J* = 8.5 Hz, C_Ar_H), 5.12 (br s, 1H, OH), 3.71 (s, 3H, CH_3_), 3.57 (s, 2H, CH_2_) ppm. NMR data match the data reported in the literature.^21^

#### 2.6.4 Methyl 4-Hydroxyphenylpropionate (4-HPP-Me, **4a**)

Starting from 4-hydroxyphenylpropionic acid (300 mg), methyl 4-hydroxyphenylpropionate was obtained as a colorless oil (290 mg, 89%). ^1^H NMR (400 MHz, CDCl_3_): δ 7.07 (m, 2H, Σ*J* = 8.5 Hz, C_Ar_H), 6.76 (m, 2H, Σ*J* = 8.5 Hz, C_Ar_H), 4.88 (br s, 1H, OH), 3.68 (s, 3H, CH_3_), 2.89 (t, 2H, *J* = 7.8 Hz, CH_2_), 2.61 (t, 2H, *J* = 7.8 Hz, CH_2_) ppm. NMR data match the data reported in the literature.^22^

#### 2.6.5 Methyl 3,4-Dihydroxyphenylacetate (DHPA-Me, **5a**)

Starting from 3,4-dihydroxyphenylacetic acid (300 mg), methyl 3,4-dihydroxyphenylacetate was obtained as a colorless oil (290 mg, 89%). ^1^H NMR (400 MHz, CDCl_3_): δ 6.75 (d, 1H, *J* = 2.0 Hz, C_Ar_H), 6.74 (d, 1H, *J* = 8.1 Hz, C_Ar_H), 6.65 (dd, 1H, *J* = 8.1, 2.1 Hz, C_Ar_H), 5.98 (br s, 2H, OH), 3.73 (s, 3H, CH_3_), 3.53 (s, 2H, CH_2_) ppm. NMR data match the data reported in the literature.^23^

#### 2.6.6 Methyl 3,4-Dihydroxyphenylpropionate (DHPP-Me, **6a**)

Starting from 3,4-hydroxyphenylpropionic acid (300 mg), methyl 3,4-dihydroxyphenylpropionate was obtained as a colorless oil (300 mg, 93%). ^1^H NMR (400 MHz, CDCl_3_): δ 6.78 (d, 1H, *J* = 8.0 Hz, C_Ar_H), 6.72 (d, 1H, *J* = 2.0 Hz, C_Ar_H), 6.63 (dd, 1H, *J* = 8.0, 2.0 Hz, C_Ar_H), 5.41 (br s, 1H, OH), 3.68 (s, 3H, CH_3_), 2.85 (t, 2H, *J* = 7.7 Hz, CH_2_), 2.60 (t, 2H, *J* = 7.7 Hz, CH_2_) ppm. NMR data match the data reported in the literature.^24^

### 2.7 Preparation of the Schmidt Imidate

#### 2.7.1 Methyl 2,3,4-Tri-*O*-acetyl-d-glucopyranuronate

Methyl 1,2,3,4-tetra-*O*-acetyl-β-d-glucopyranosyluronate (940 mg, 2.50 mmol) was dissolved in DMF (6 mL) under an argon atmosphere. Benzylamine (321 mg, 3.00 mmol) was added and the reaction mixture was stirred for 16 h at rt. After evaporation under vacuum, the mixture was immediately purified by column chromatography (cyclohexane/EtOAc 2:1). A second column chromatography afforded the product as a colorless oil (700 mg, 84%). ^1^H NMR (400 MHz, CDCl_3_): δ 5.61–5.56 (m, 1H, CH), 5.22–5.17 (m, 1H, CH), 5.34–4.78 (m, 3H, 3 × CH), 3.78–3.74 (m, 3H, CH_3_), 2.05–1.99 (m, 9H, 3 × CH_3_) ppm. NMR data match the data reported in the literature.^25^

#### 2.7.2 Methyl 2,3,4-Tri-*O*-acetyl-1-*O*-(trichloroacetimidoyl)-α-d-glucopyranuronate (Schmidt-GlcA, **7**)^26^

Methyl 2,3,4-tri-*O*-acetyl-d-glucopyranuronate (170 mg, 0.51 mmol), 2,2,2-trichloroacetonitrile (529 mg, 3.66 mmol), K_2_CO_3_ (387 mg, 2.80 mmol) and molecular sieves (3 Å) were suspended in dry CH_2_Cl_2_ (2.5 mL) and the reaction mixture was stirred at rt for 16 h. The resulting mixture was filtered through a short layer of silica gel, eluted with Et_2_O, and the fractions containing the product were combined and evaporated. The crude product was purified using column chromatography (cyclohexane/EtOAc 2:1) to give the product **7** as a white powder (173 mg, 71%). ^1^H NMR (400 MHz, CDCl_3_): δ 8.74 (s, 1H, NH), 6.65 (d, 1H, *J* = 3.6 Hz, CH-1α), 5.64 (dd, 1H, Σ*J* = 9.9 Hz, CH-3), 5.28 (dd, 1H, Σ*J* = 9.6 Hz, CH-4), 5.16 (dd, 1H, *J* = 10.2, 3.6 Hz, CH-2), 4.51 (d, 1H, *J* = 10.2 Hz, CH-5), 3.76 (s, 3H, OCH_3_), 2.06 (s, 3H, CH_3_CO), 2.05 (s, 3H, CH_3_CO) 2.03 (s, 3H, CH_3_CO) ppm. NMR data match the data reported in the literature.^27^

### 2.8 Preparation of Protected Glucuronides

#### 2.8.1 (2*S*,3*R*,4*S*,5*S*,6*S*)-2-(2-(2-Methoxy-2-oxoethyl)­phenoxy)-6-(methoxycarbonyl)­tetrahydro-2*H*-pyran-3,4,5-triyl Triacetate (perAc-2-HPA-GlcA, **1b**)

Methyl 2-hydroxyphenylacetate (113 mg, 0.68 mmol), methyl 2,3,4-tri-*O*-acetyl-1-*O*-(trichloroacetimidoyl)-α-d-glucopyranuronate (217 mg, 0.45 mmol) and molecular sieves (4Å, powder, 45 mg) were suspended in dry CH_2_Cl_2_ (6 mL) under argon atmosphere. The mixture was stirred at rt for 1 h, then cooled to −50 °C, and BF_3_·Et_2_O (57 μL, 0.045 mmol) was added. The reaction mixture was allowed to warm to 0 °C and stirred at this temperature for 3 days. Column chromatography (toluene/acetone 5:1, then cyclohexane/EtOAc 2:1) gave the target compound as a white solid (90 mg, 41%). For HPLC, ^1^H and ^13^C NMR, and HRMS see Table S1 and Figures S1–S5 in the Supporting Information.

#### 2.8.2 (2*S*,3*R*,4*S*,5*S*,6*S*)-2-(3-(2-Methoxy-2-oxoethyl)­phenoxy)-6-(methoxycarbonyl)­tetrahydro-2*H*-pyran-3,4,5-triyl Triacetate (perAc-3-HPA-GlcA, **2b**)

Methyl 3-hydroxyphenylacetate (166 mg, 1 mmol), methyl 2,3,4-tri-*O*-acetyl-1-*O*-(trichloroacetimidoyl)-α-d-glucopyranuronate (320 mg, 0.67 mmol) and molecular sieves (4 Å, powder, 67 mg) were suspended in dry CH_2_Cl_2_ (8 mL) under argon atmosphere. The mixture was stirred at rt for 1 h, then cooled to −20 °C, and BF_3_·Et_2_O (10.5 μL, 0.0836 mmol) was added. The reaction mixture was allowed to warm to 0 °C and stirred at this temperature for 4 days. Column chromatography (toluene/acetone 5:1, then cyclohexane/EtOAc 2:1) gave the target compound as a white solid (195 mg, 60%). For HPLC, ^1^H and ^13^C NMR, and HRMS see Table S2 and Figures S6–S10 in the Supporting Information.

#### 2.8.3 (2*S*,3*R*,4*S*,5*S*,6*S*)-2-(4-(2-Methoxy-2-oxoethyl)­phenoxy)-6-(methoxycarbonyl)­tetrahydro-2*H*-pyran-3,4,5-triyl Triacetate (perAc-4-HPA-GlcA, **3b**)

Methyl 4-hydroxyphenylacetate (60 mg, 0.36 mmol), methyl 2,3,4-tri-*O*-acetyl-1-*O*-(trichloroacetimidoyl)-α-d-glucopyranuronate (163 mg, 0.34 mmol) and molecular sieves (4Å, powder, 34 mg) were suspended in dry CH_2_Cl_2_ (4 mL) under argon atmosphere. The mixture was stirred at rt for 1 h, then cooled to −50 °C, and BF_3_·Et_2_O (43 μL, 0.338 mmol) was added. The reaction mixture was allowed to warm to 0 °C and stirred at this temperature for 16 h. Column chromatography (toluene/acetone 2:1) gave the target compound as a clear oil (163 mg, 99%). For HPLC, ^1^H and ^13^C NMR, and HRMS see Table S3 and Figures S11–S15 in the Supporting Information.

#### 2.8.4 (2*S*,3*R*,4*S*,5*S*,6*S*)-2-(4-(3-Methoxy-3-oxopropyl)­phenoxy)-6-(methoxycarbonyl)­tetrahydro-2*H*-pyran-3,4,5-triyl Triacetate (perAc-4-HPP-GlcA, **4b**)

Methyl 4-hydroxyphenylpropionate (59 mg, 0.33 mmol), methyl 2,3,4-tri-*O*-acetyl-1-*O*-(trichloroacetimidoyl)-α-d-glucopyranuronate (150 mg, 0.31 mmol) and molecular sieves (4Å, powder, 31 mg) were suspended in dry CH_2_Cl_2_ (3.5 mL) under argon atmosphere. The mixture was stirred at rt for 1 h, then cooled to −20 °C, and BF_3_·Et_2_O (39 μL, 0.31 mmol) was added. The reaction mixture was allowed to warm to 0 °C and stirred at room temperature for 3 days. Column chromatography (cyclohexane/EtOAc 2:1) gave the target compound as a clear oil (116 mg, 75%). For HPLC, ^1^H and ^13^C NMR, and HRMS see Table S4 and Figures S16–S20 in the Supporting Information.

#### 2.8.5 (2*S*,3*R*,4*S*,5*S*,6*S*)-2-(2-Hydroxy-4-(2-methoxy-2-oxoethyl)­phenoxy)-6-(methoxycarbonyl)­tetrahydro-2*H*-pyran-3,4,5-triyl Triacetate (perAc-DHPA-4′-GlcA, **5b**) and (2*S*,3*R*,4*S*,5*S*,6*S*)-2-(2-Hydroxy-5-(2-methoxy-2-oxoethyl)­phenoxy)-6-(methoxycarbonyl)­tetrahydro-2*H*-pyran-3,4,5-triyl Triacetate (perAc-DHPA-3′-GlcA, **5b′**)

Methyl 3,4-hydroxyphenylacetate (175 mg, 0.96 mmol), methyl 2,3,4-tri-*O*-acetyl-1-*O*-(trichloroacetimidoyl)-α-d-glucopyranuronate (300 mg, 0.63 mmol) and molecular sieves (4 Å, powder, 62 mg) were suspended in dry CH_2_Cl_2_ (7 mL) under argon atmosphere. The mixture was stirred at rt for 1 h, then cooled to −50 °C, and BF_3_·Et_2_O (80 μL, 0.64 mmol) was added. The mixture was allowed to warm to 0 °C and stirred at this temperature for 2 days. Column chromatography (toluene/acetone 5:1) gave the target compound as a clear oil (150 mg, 48%). For HPLC, ^1^H and ^13^C NMR, and HRMS see Tables S5 and S6, and Figures S21–S25 in the Supporting Information.

#### 2.8.6 (2*S*,3*R*,4*S*,5*S*,6*S*)-2-(2-Hydroxy-4-(3-methoxy-3-oxopropyl)­phenoxy)-6-(methoxycarbonyl)­tetrahydro-2*H*-pyran-3,4,5-triyl Triacetate (perAc-DHPP-4′-GlcA, **6b**) and (2*S*,3*R*,4*S*,5*S*,6*S*)-2-(2-Hydroxy-5-(3-methoxy-3-oxopropyl)­phenoxy)-6-(methoxycarbonyl)­tetrahydro-2*H*-pyran-3,4,5-triyl Triacetate (perAc-DHPP-3′-GlcA, **6b′**)

Methyl 3,4-dihydroxyphenylpropionate (186 mg, 0.95 mmol), methyl 2,3,4-tri-*O*-acetyl-1-*O*-(trichloroacetimidoyl)-α-d-glucopyranuronate (302 mg, 0.63 mmol) and molecular sieves (4 Å, powder, 65 mg) were suspended in dry CH_2_Cl_2_ (7 mL) under argon atmosphere. The mixture was stirred at rt for 1 h, then cooled to −50 °C, and BF_3_·Et_2_O (79.0 μL, 0.63 mmol) was added. The reaction mixture was stirred at −50 °C for 3 h and then allowed to warm to 0 °C and stirred at this temperature for 2 days. Column chromatography (toluene/acetone 5:1) gave the target compound as a white powder (245 mg, 76%). For HPLC, ^1^H and ^13^C NMR, and HRMS see Tables S7 and S8, and Figures S26–S30 in the Supporting Information.

### 2.9 Preparation of Carboxylic Acids

Potassium hydroxide (9 equiv) was dissolved in water and MeOH (1:1). The mixture was cooled down to 0 °C and protected glucuronides (1 equiv) were added. The reaction mixture was stirred at 0 °C for 3–6 days as determined by TLC monitoring. After the reaction was completed, the solvents were removed under vacuum and the solid residue was dissolved in water. Acidic Dowex 50WX8 was then added and the mixture was shaken vigorously until reaching pH 2. Dowex filtered off and the mixture was subsequently lyophilized. The desired acids were isolated by C18 column chromatography (5% acetonitrile in water).

#### 2.9.1 (2*S*,3*S*,4*S*,5*R*,6*S*)-6-(2-(carboxymethyl)­phenoxy)-3,4,5-trihydroxytetrahydro-2*H*-pyran-2-carboxylic Acid (2-HPA-GlcA, **1c**)

Starting from perAc-2-HPA-GlcA (90 mg), the desired product 2-HPA-GlcA was isolated as a white solid (37 mg, 61%). For HPLC, ^1^H and ^13^C NMR, HRMS, and CD data see Table S9 and Figures S31–S36 in the Supporting Information.

#### 2.9.2 (2*S*,3*S*,4*S*,5*R*,6*S*)-6-(3-(Carboxymethyl)­phenoxy)-3,4,5-trihydroxytetrahydro-2*H*-pyran-2-carboxylic Acid (3-HPA-GlcA, **2c**)

Starting from perAc-3-HPA-GlcA (195 mg), the desired product 3-HPA-GlcA was isolated as a white solid (76 mg, 58%). For HPLC, ^1^H and ^13^C NMR, and HRMS see Table S10 and Figures S37–S41 in the Supporting Information.

#### 2.9.3 (2*S*,3*S*,4*S*,5*R*,6*S*)-6-(4-(Carboxymethyl)­phenoxy)-3,4,5-trihydroxytetrahydro-2*H*-pyran-2-carboxylic Acid (4-HPA-GlcA, **3c**)

Starting from perAc-4-HPA-GlcA (163 mg), the desired product 4-HPA-GlcA was isolated as a white solid (75 mg, 68%) with [α]_589_^20^ −49.1 (0.0082 g/100 mL). For HPLC, ^1^H and ^13^C NMR, and HRMS see Table S11 and Figures S42–S47 in the Supporting Information.

#### 2.9.4 (2*S*,3*S*,4*S*,5*R*,6*S*)-6-(4-(2-Carboxyethyl)­phenoxy)-3,4,5-trihydroxytetrahydro-2*H*-pyran-2-carboxylic Acid (4-HPP-GlcA, **4c**)

Starting from perAc-4-HPP-GlcA (190 mg), the desired product 4-HPP-GlcA was isolated as a white solid (121 mg, 92%) with [α]_589_^20^ −57.1 (0.0059 g/100 mL). For HPLC, ^1^H and ^13^C NMR, HRMS, and CD data see Table S12 and Figures S48–S53 in the Supporting Information.

#### 2.9.5 (2*S*,3*S*,4*S*,5*R*,6*S*)-6-(4-(Carboxymethyl)-2-hydroxyphenoxy)-3,4,5-trihydroxytetrahydro-2*H*-pyran-2-carboxylic Acid (DHPA-4′-GlcA, **5c**) and (2*S*,3*S*,4*S*,5*R*,6*S*)-6-(5-(Carboxymethyl)-2-hydroxyphenoxy)-3,4,5-trihydroxytetrahydro-2*H*-pyran-2-carboxylic Acid (DHPA-3′-GlcA, **5c′**)

Starting from a mixture of perAc-4′-DHPA-GlcA (**5b**) and perAc-3′-DHPA-GlcA (**5b**′) (124 mg), a mixture of desired products 4′-DHPA-GlcA (**5c**) and 3′-DHPA-GlcA (**5c′**) was isolated as a white solid (64 mg, 75%) with [α]_589_^20^ −63.2 (0.0122 g/100 mL). For HPLC, ^1^H and ^13^C NMR, HRMS, and CD data see Tables S13, S14, and Figures S54–S59 in the Supporting Information.

#### 2.9.6 (2*S*,3*S*,4*S*,5*R*,6*S*)-6-(4-(2-Carboxyethyl)-2-hydroxyphenoxy)-3,4,5-trihydroxytetrahydro-2*H*-pyran-2-carboxylic Acid (DHPP-4′-GlcA, **6c**) and (2*S*,3*S*,4*S*,5*R*,6*S*)-6-(5-(2-Carboxyethyl)-2-hydroxyphenoxy)-3,4,5-trihydroxytetrahydro-2*H*-pyran-2-carboxylic Acid (DHPP-3′-GlcA, **6c′**)

Starting from a mixture of perAc-4′-DHPP-GlcA (**6b**) and perAc-3′-DHPP-GlcA (**6b′**) (186 mg), a mixture of desired products 4′-DHPP-GlcA (**6c**) and 3′-DHPP-GlcA (**6c′**) was isolated as a white solid (37 mg, 28%) with [α]_589_^20^ −72.9 (0.0028 g/100 mL). For HPLC, ^1^H and ^13^C NMR, HRMS, and CD data see Tables S15, S16, and Figures S60–S65 in the Supporting Information.

### 2.10 Pilot Metabolic Study in Rat

#### 2.10.1 Animals and Study Design

The *in vivo* experiments were exerted on male Wistar:Han rats (Velaz s.r.o., Czech Republic). The animals were kept under standard conditions (23–25 °C, 12 h dark/light cycle, a standard diet and tap water *ad libitum*). The study was approved by the Ministry of Education, Youth, and Sports of the Czech Republic (MSMT-26317/2023-4) and was in accordance with the Guide for the Care and Use of Laboratory Animals (US National Institutes of Health 2011, eighth Edition, ISBN-13:978-0-309-15400-0).

Briefly, conscient rats received hawthorn extract (475 mg/kg in 2 mL of aqueous glycerol) via oral gavage. The animals were then anaesthetized (urethane 1.2 g/kg, *i.p.*) and blood samples (∼300 μL each) were collected from *arteria carotis communis sinistra* at 2, 3, 4, 5, and 6 h postadministration. After each collection, the catheter was flushed with heparinized saline. Blood samples were immediately centrifuged (10 min, 2500*g*) and the plasma samples were stored at −80 °C until further analysis. At the end of the experiment, rats were euthanized with KCl (1 M in saline, 1.5 mL *i.v.*).

#### 2.10.2 Sample Preparation and UHPLC-HRMS Analysis of Rat Plasma

Before analysis, plasma samples were precipitated with CH_3_CN containing 0.1% HCOOH in a 1:2 ratio (sample/acetonitrile) and centrifuged (21,255*g*, 15 min). The collected supernatants were evaporated to dryness under vacuum and reconstituted in a dilution solvent consisting of 50% methanol with 0.1% formic acid. The reconstituted samples were then subjected to analysis.

The analysis of pretreated plasma samples was carried out using a UPLC system (I-Class, Waters, Milford, USA) coupled to a HRMS (Synapt G2-Si quadrupole time-of-flight (Q-TOF), Waters, Milford, USA), for confirmation of the presence of phenolic acid glucuronides. A 10 μL aliquot was injected onto an analytical column (ACE C18-PFP, 2.1 × 100 mm; 1.7 μm). The separation was achieved using gradient elution with 0.1% aqueous CH_3_COOH as phase A and MeOH as phase B at a flow rate of 0.35 mL/min. The gradient started at 2% B, increased linearly to 98% B over 15 min, and was followed by a 2 min equilibration step. The total chromatographic run time, including column re-equilibration, was 17 min.

The electrospray ionization (ESI) source was operated in negative mode with a capillary voltage of −1.3 kV, sampling cone voltage of 20 V, source offset of 40 V, and source temperature of 150 °C. Nitrogen was used as the desolvation gas at 1000 L/h and 600 °C, and as a cone gas at 50 L/h. Argon served as the collision gas. The pressure of the nebulization gas was maintained at 6.5 bar.

Both data-independent acquisition (DIA) was employed for the detection of phenolic acid glucuronides. DIA allowed simultaneous acquisition of MS and MS/MS data in negative ion mode. MS spectra were acquired over the *m*/*z* range 50–1200 using a fixed collision energy of 4 eV for MS scans, and a ramped collision energy from 15 to 30 eV for MS/MS scans. A targeted MS/MS scan of the deprotonated molecules of the obtained glucuronides present in the sample was additionally acquired at a collision energy of 10 eV. Leucine enkephalin (200 pg/μL) was used as an internal calibrant, and a 0.5 mM aqueous sodium formate served as the external calibrant. Mass spectrometric data were acquired and processed using MassLynx 4.1 software, and analyte identification was performed using the UNIFI Scientific Information System Software. Compounds were identified based on three main criteria: retention time, precursor ion mass accuracy below 5 ppm, and identification of characteristic fragment ions with mass accuracy also below 5 ppm.

## 3 Results and Discussion

The carboxylic group of phenolic acids is incompatible with most glucuronidation methods; therefore, we protected the starting phenolic acids by a simple reaction with methanol, catalyzed by sulfuric acid.^28^ The target esters were purified by column chromatography and obtained in high yields (Scheme 2). The predicted p*K*_a_ values of these esters ranged between 9.6 and 9.9.^29^ As glucuronidation agents, we chose perAc-GlcA-Br and Schmidt-GlcA (**7**), which were prepared from methyl 1,2,3,4-tetra-*O*-acetyl-β-d-glucopyranosyluronate following previously published procedures.^26^

**Scheme 2:**
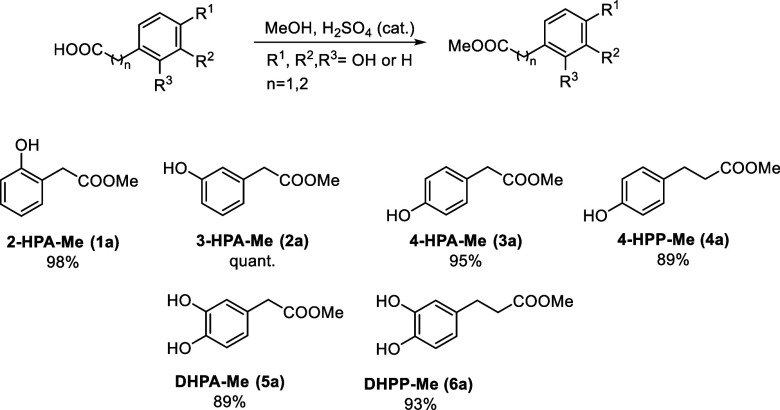
Overview of Prepared Phenolic Acid Methyl Esters and Their Isolated Yields

Subsequently, 4-hydroxyphenylacetic acid methyl ester (4-HPA-Me, **3a**) was chosen as the test substrate and various glucuronidation conditions were investigated. First, we explored the reactivity of 4-HPA-Me (**3a**) under the Koenigs–Knorr conditions. A series of reactions of 4-HPA-Me (**3a**) with perAc-GlcA-Br, using silver-based catalysts (Ag_2_O, freshly prepared Ag_2_CO_3_, Ag_2_CO_3_/Celite–Fétizon’s reagent) and various dry solvents (CH_2_Cl_2_, pyridine, acetonitrile) were performed, but only the starting material or degraded perAc-GlcA-Br was observed. In rare cases, heavy metal-free carbonates^30,31^ were used in glucuronidation reactions; by analogy with a published procedure,^32^ we tested the reaction of 4-HPA-Me (**3a**) and perAc-GlcA-Br in the presence of KI catalyzed by K_2_CO_3_; however, the desired product was not detected. Finally, biphasic systems and phase transfer conditions^33^ were used in some glycosidation reactions and we hoped that a similar approach might apply to glucuronidation. However, the reactions of 4-HPA-Me and perAc-GlcA-Br catalyzed by tetrabutylammonium hydrogensulfate (TBAHS) in a biphasic system of CH_2_Cl_2_, NaOH/H_2_O or EtOAc, Na_2_CO_3_/H_2_O only led to degradation of the starting compounds.

We have therefore turned to another glucuronidation agent. In analogy to the literature,^34^ the reaction of 4-HPA-Me (**3a**) with Schmidt imidate (**7**) in dry CH_2_Cl_2_ catalyzed by BF_3_·Et_2_O (0.4 equiv) in the presence of molecular sieves at 0 °C–rt gave the desired protected glucuronide perAc-4-HPA-GlcA (**3b**) in a 41% yield. The reaction was further optimized to a nearly quantitative yield by increasing the amount of BF_3_·Et_2_O (1 equiv) and lowering the reaction temperature (0 °C was not allowed to be exceeded; Table 1, Scheme 3).

**Table 1:** Optimization of the Reaction Conditions for the Reaction of 4-HPA-Me (**3a**) with the Schmidt Imidate (**7**)

entry	BF_3_ **·Et**_2_O [equiv]	*T* [°C]	yield [%]
1	0.4	0- rt	41
2	1	0 - rt	62
3	1	0	99

**Scheme 3:**
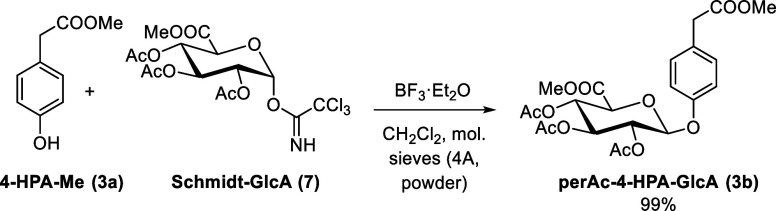
Reaction of 4-HPA-Me (**3a**) with the Schmidt Imidate (**7**)

The other selected hydroxyphenolic acid esters also gave the desired protected glucuronides in good yields (41–75%) under optimized reaction conditions, although in some cases a longer reaction time was required. Dihydroxyphenolic acid esters (DHPA-Me (**5a**) and DHPP-Me (**6a**)) afforded the target glucuronides as mixtures of their 3′- and 4′-substituted regioisomers with a slight excess of the 4′-glucuronidated isomer, probably due to its lower steric hindrance (Figure 2).

**Figure 2:**
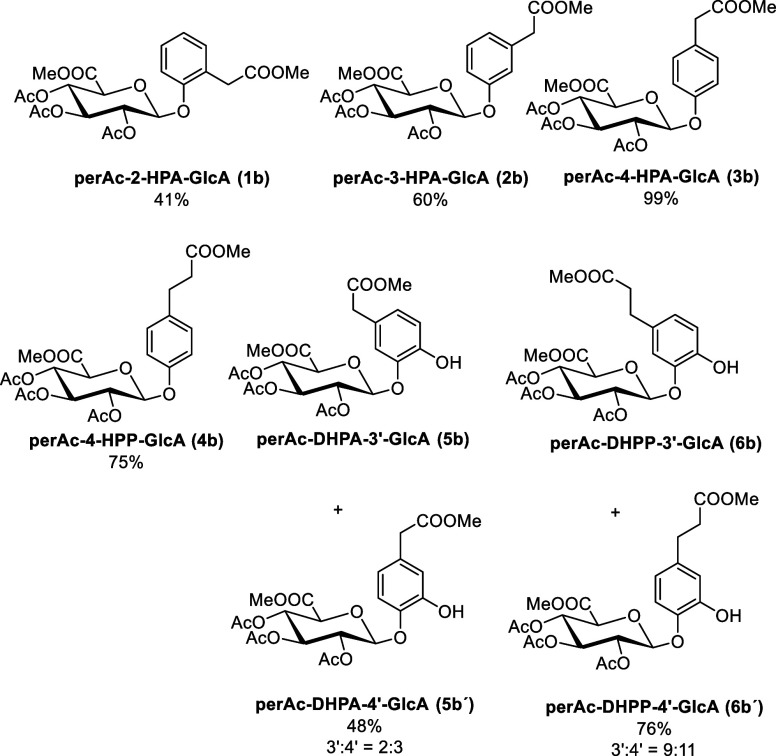
An overview of the protected phenolic acid glucuronides synthesized by the reaction of phenolic acid methyl esters with Schmidt imidate and the isolated yields.

With these protected glucuronides in hand, we focused on their conversion to phenolic acid glucuronides. Attempts at deprotection via the Na_2_CO_3_-methanol/water method led to a significant amount of a side-product of acetate elimination, which sometimes occurs for this type of molecules.^35^ Therefore, to remove the acetate and methyl ester protecting groups, the protected glucuronides were reacted with excess KOH (9 equiv) in methanol/water at 0 °C for several days. After completion of the reaction, treatment with Dowex 50WX8 and purification on C18 silica gel afforded the desired phenolic acid glucuronides in high purity (Scheme 4).

**Scheme 4:**
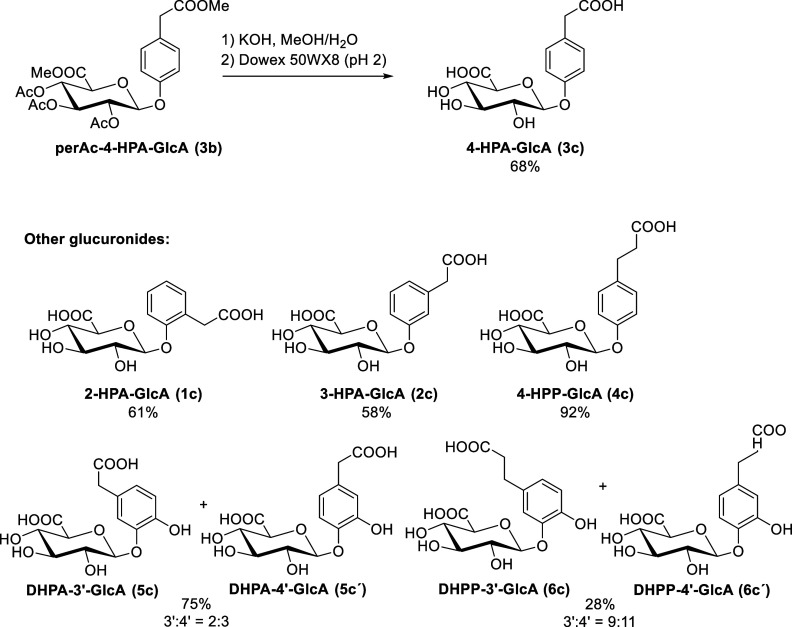
Prepared Phenolic Acid Glucuronides and Their Isolated Yields

Structure elucidation of both protected and free glucuronides was based on ^1^H NMR, ^13^C NMR, COSY, ^1^H–^13^C HSQC, and ^1^H–^13^C HMBC experiments. The extensive overlap of glucose proton resonances prevented the determination of most coupling constants, except for *J*_H1,H2_. The observed values, ranging from 7.1 to 7.6 Hz, are consistent with the β-anomeric configuration in all samples. This assignment was further supported by the magnitude of the one-bond carbon–proton coupling constant *J*_C1,H1_ (164–165 Hz) detected in all samples.

The obtained glucuronides, as potential polyphenol metabolites, were used as fully characterized standards in a pilot animal metabolomic study. Two of them, namely 4-HPA-GlcA (**3c**) and 3-HPA-GlcA (**2c**), were identified in rat plasma collected after extract administration. Both compounds were confirmed based on the following criteria: retention times matching those of the characterized standards (2.84 min for 4-HPA-GlcA and 3.23 min for 3-HPA-GlcA; Figure 3A); mass accuracy of the deprotonated molecules (*m*/*z* 327.0722) within 3 ppm in MS scan for both compounds; and the presence of three characteristic fragments (*m*/*z* 283.0823, 175.0248, and 113.0244) in the MS/MS scan with mass accuracy within 5 ppm (Figure 3B,C). Therefore, this pilot metabolomic study confirmed the presence of these two synthesized phenolic acid glucuronides as true metabolites in rat plasma.

**Figure 3:**
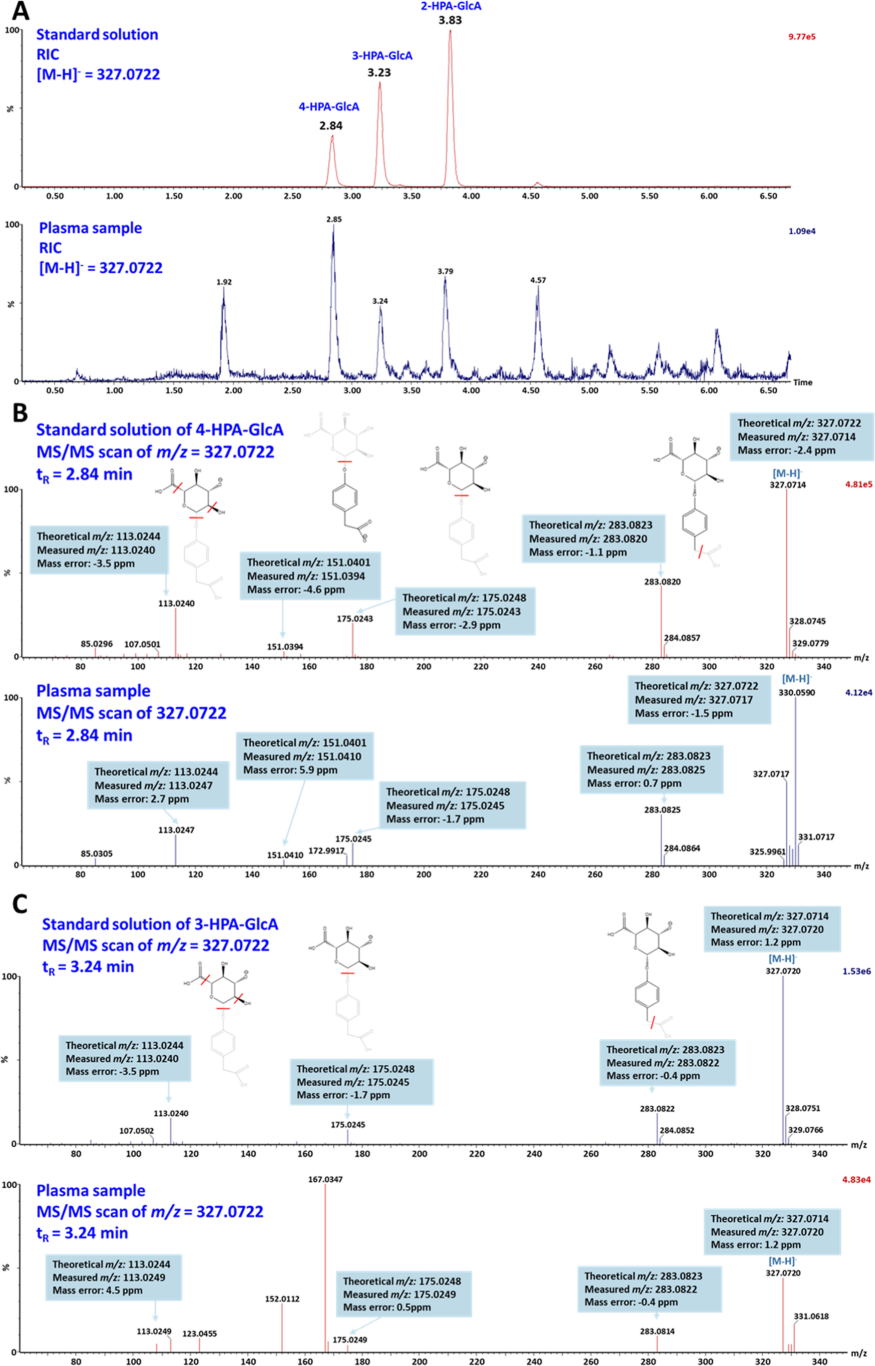
Chromatograms and MS/MS of 3-HPA-GlcA and 4-HPA-GlcA in the rat plasma: (A) reconstructed ion chromatogram of ions with *m*/*z* 327.0722, (B) MS/MS spectra of 4-HPA-GlcA (**3c**), and (C) MS/MS spectra of 3-HPA-GlcA (**2c**).

## 4 Conclusion

We have successfully prepared a small library of phenolic acid glucuronides by studying the reactivity of phenolic acid methyl esters with the two best-known glucuronidation reagents: perAc-GlcA-Br (Koenigs–Knorr) and Schmidt imidate (Schmidt-GlcA, **7**). While perAc-GlcA-Br proved to be ineffective, reactions with Schmidt imidate yielded six target glucuronides (perAc-2-HPA-GlcA **1b**, perAc-3-HPA-GlcA **2b**, perAc-4-HPA-GlcA **3b**, perAc-4-HPP-GlcA **4b**, perAc-DHPA-3′-GlcA **5b** + perAc-DHPA-4′-GlcA **5b′**, perAc-DHPP-3′-GlcA **6b** + perAc-DHPP-4′-GlcA **6b′**) in moderate to excellent yields. These were then deprotected with KOH to give the desired phenolic acid glucuronides (2-HPA-GlcA **1c**, 3-HPA-GlcA **2c**, 4-HPA-GlcA **3c**, 4-HPP-GlcA **4c**, DHPA-3′-GlcA **5c** + DHPA-4′-GlcA **5c′**, DHPP-3′-GlcA **6c** + DHPP-4′-GlcA **6c′**). The obtained glucuronides are potential polyphenol metabolites and were subsequently used as fully characterized standards for a pilot metabolic study in animals, where two of them were confirmed as true metabolites in rat plasma. These compounds can be used for larger metabolic studies and to evaluate their biological activity.

## Supplementary Material



## Notes

Additional data, Tables, and Figures on the structural characterization (^1^H and ^13^C NMR data, HPLC analyses, and HRMS analyses) of isolated products (PDF)

## References

[1] Gutiérrez-Díaz I., Fernández-Navarro T., Salazar N., Bartolomé B., Moreno-Arribas M. V., López P., Suárez A., de Los Reyes-Gavilán C. G., Gueimonde M., González S. (2018). Could fecal phenylacetic and phenylpropionic acids
be used as indicators of health status?. J.
Agric. Food Chem..

[2] Kumar N., Goel N. (2019). Phenolic acids: Natural
versatile molecules with promising therapeutic
applications. Biotechnol. Rep..

[3] Robbins R. J. (2003). Phenolic
acids in foods: An overview of analytical methodology. J. Agric. Food Chem..

[4] Williamson G., Clifford M. N. (2010). Colonic
metabolites of berry polyphenols: The missing
link to biological activity?. Br. J. Nutr..

[5] Carecho R., Carregosa D., Dos Santos C. N. (2020). Low molecular weight (poly)­phenol
metabolites across the blood-brain barrier: The underexplored journey. Brain Plast..

[6] Kolaříková V., Brodsky K., Petrásková L., Pelantová H., Cvačka J., Havlíček L., Křen V., Valentová K. (2022). Sulfation of phenolic acids: Chemoenzymatic vs. chemical
synthesis. Int. J. Mol. Sci..

[7] Yang G., Ge S., Singh R., Basu S., Shatzer K., Zen M., Liu J., Tu Y., Zhang C., Wei J., Shi J., Zhu L., Liu Z., Wang Y., Gao S., Hu M. (2017). Glucuronidation:
Driving factors and their impact on glucuronide disposition. Drug Metab. Rev..

[8] Rowland A., Miners J. O., Mackenzie P. I. (2013). The UDP-glucuronosyltransferases:
Their role in drug metabolism and detoxification. Int. J. Biochem. Cell Biol..

[9] Ohnuki T., Ejiri M., Kizuka M., Fujiwara M., Nishi T. (2019). Practical
one-step glucuronidation via biotransformation. Bioorg. Med. Chem. Lett..

[10] Walther R., Jarlstad Olesen M. T., Zelikin A. N. (2019). Extended scaffold glucuronides: En
route to the universal synthesis of *O*-aryl glucuronide
prodrugs. Org. Biomol. Chem..

[11] Florent J.-C., Dong X., Gaudel G., Mitaku S., Monneret C., Gesson J.-P., Jacquesy J.-C., Mondon M., Renoux B., Andrianomenjanahary S., Michel S., Koch M., Tillequin F., Gerken M., Czech J., Straub R., Bosslet K. (1998). Prodrugs of
anthracyclines for use in antibody-directed enzyme prodrug therapy. J. Med. Chem..

[12] Cruz L., Basílio N., Mateus N., Pina F., de Freitas V. (2015). Characterization
of kinetic and thermodynamic parameters of cyanidin-3-glucoside methyl
and glucuronyl metabolite conjugates. J. Phys.
Chem. B.

[13] Wang, Z. Zemplén Deacetylation. In Comprehensive Organic Name Reactions and Reagents; John Wiley & Sons, Inc., 2010; pp 3123–3128.

[14] Fischer B., Nudelman A., Ruse M., Herzig J., Gottlieb H. E., Keinan E. (1984). A novel method for stereoselective glucuronidation. J. Org. Chem..

[15] Hayes J. A., Eccles K. S., Lawrence S. E., Moynihan H. A. (2012). Preparation and
characterisation of solid state forms of paracetamol-*O*-glucuronide. Carbohydr. Res..

[16] London J. A., Wang E. C. S., Barsukov I. L., Yates E. A., Stachulski A. V. (2021). Synthesis
and toxicity profile in 293 human embryonic kidney cells of the β
D-glucuronide derivatives of *ortho*-, *meta*- and *para*-cresol. Carbohydr.
Res..

[17] Gómez-Juaristi M., Martínez-López S., Sarria B., Bravo L., Mateos R. (2018). Bioavailability of
hydroxycinnamates in an instant
green/roasted coffee blend in humans. Identification of novel colonic
metabolites. Food Funct..

[18] Bresciani L., Martini D., Mena P., Tassotti M., Calani L., Brigati G., Brighenti F., Holasek S., Malliga D.-E., Lamprecht M., Del Rio D. (2017). Absorption profile of (poly)­phenolic
compounds after consumption of three food supplements containing 36
different fruits, vegetables, and berries. Nutrients.

[19] Tabaszewska M., Najgebauer-Lejko D., Zbylut-Górska M., Skoczylas Ł., Tokarczyk G. (2023). Effect of hawthorn berry pre-treatment and preservation
methods on the extractability of color-determining compounds and selected
antioxidative substances. LWT.

[20] Zheng Y., Zhao Y., Tao S., Li X., Cheng X., Jiang G., Wan X. (2021). Green esterification
of carboxylic
acids promoted by *tert*-butyl nitrite. Eur. J. Org Chem..

[21] Valero M., Becker D., Jess K., Weck R., Atzrodt J., Bannenberg T., Derdau V., Tamm M. (2019). Directed iridium-catalyzed
hydrogen isotope exchange reactions of phenylacetic acid esters and
amides. Chem.—Eur. J..

[22] Poǹkina D., Kuranov S., Khvostov M., Zhukova N., Meshkova Y., Marenina M., Luzina O., Tolstikova T., Salakhutdinov N. (2023). Hepatoprotective effect of a new FFAR1 agonist—*N*-alkylated isobornylamine. Molecules.

[23] Geiseler B., Fruk L. (2012). Bifunctional catechol based linkers
for modification of TiO_2_ surfaces. J. Mater. Chem..

[24] Bourne G. T., Golding S. W., McGeary R. P., Meutermans W. D. F., Jones A., Marshall G. R., Alewood P. F., Smythe M. L. (2001). The development
and application of a novel safety-catch linker for BOC-based assembly
of libraries of cyclic peptides. J. Org. Chem..

[25] Chen Y., Li Y., Yu H., Sugiarto G., Thon V., Hwang J., Ding L., Hie L., Chen X. (2013). Tailored design and
synthesis of heparan sulfate oligosaccharide analogues using sequential
one-pot multienzyme systems. Angew. Chem., Int.
Ed..

[26] T
Brown R., Scheinmann F., V Stachulski A. (1997). Intermediates
for glucuronide synthesis: 7-Hydroxycoumarin glucuronide. J. Chem. Res..

[27] Soliman S. E., Bassily R. W., El-Sokkary R. I., Nashed M. A. (2003). Acetylated methyl
glucopyranuronate trichloroacetimidate as a glycosyl donor for efficient
synthesis of disaccharides. Carbohydr. Res..

[28] Hajji C., Roller S., Beigi M., Liese A., Haag R. (2006). Polyglycerol-supported
chromium-salen as a high-loading dendritic catalyst for stereoselective
Diels–Alder reactions. Adv. Synth. Catal..

[29] Cao, H. ; Chen, X. ; Jassbi, A. R. ; Xiao, J. Microbial biotransformation of bioactive flavonoids. 2015, 33, 214–223. doi: 10.1016/j.biotechadv.2014.10.012. 25447420

[30] Jones A. E., Wilson H. K., Meath P., Meng X., Holt D. W., Johnston A., Oellerich M., Armstrong V. W., Stachulski A. V. (2009). Convenient syntheses of the *in vivo* carbohydrate metabolites of mycophenolic acid: reactivity
of the
acyl glucuronide. Tetrahedron Lett..

[31] Nasseri S. A., Betschart L., Opaleva D., Rahfeld P., Withers S. G. (2018). A mechanism-based
approach to screening metagenomic libraries for discovery of unconventional
glycosidases. Angew. Chem., Int. Ed..

[32] Du K., Cao X., Zhang P., Zheng H. (2014). Synthesis and anti-tumor
activity
of glycosyl oxadiazoles derivatives. Bioorg.
Med. Chem. Lett..

[33] Carrière D., Meunier S. J., Tropper F. D., Cao S., Roy R. (2000). Phase transfer
catalysis toward the synthesis of *O*-, *S*-, *Se*- and *C*-glycosides. J. Mol. Catal. A: Chem..

[34] Zhang Q., Raheem K. S., Botting N. P., Slawin A. M. Z., Kay C. D., O’Hagan D. (2012). Flavonoid
metabolism: The synthesis of phenolic glucuronides
and sulfates as candidate metabolites for bioactivity studies of dietary
flavonoids. Tetrahedron.

[35] Stachulski A. V., Meng X. (2013). Glucuronides from metabolites to
medicines: a survey of the *in vivo* generation, chemical
synthesis and properties of
glucuronides. Nat. Prod. Rep..

