# Supplementary material for: Glucuronidated Hydroxyphenylacetic and Hydroxyphenylpropanoic Acids as Standards for Bioavailability Studies with Flavonoids
Source: ACS Omega. 2025 Dec 25;11(1):1620–9. doi: 10.1021/acsomega.5c09380 (PMC12809306; doi:10.1021/acsomega.5c09380)
Supplement: Supplementary file 1 [file ao5c09380_si_001.pdf]

Supporting information

Glucuronidated Hydroxyphenylacetic and Hydroxyphenylpropanoic Acids as Standards for Bioavailability Studies with Flavonoids

Viola Janouchová,[1] Martina Hurtová,[1] Hana Kočová Vlčková,[2] Zuzana Lomozová,[2] Jana Pourová,[2] Lucie Nováková,[2] Lucie Petrásková, [1] Helena Pelantová, [1] Josef Cvačka, [3] Kateřina Valentová[1,*]

[1] *Institute of Microbiology of the Czech Academy of Sciences, Vídeňská 1083, CZ-142 00 Prague 4, Czech Republic*

[2] *Faculty of Pharmacy, Charles University, Akademika Heyrovského 1203, CZ-50005 Hradec Králové, Czech Republic*

[3] *Institute of Organic Chemistry and Biochemistry of the Czech Academy of Sciences, Flemingovo náměstí 542/2, 160 00 Prague 6, Czech Republic*

*Corresponding author: kata.valentova@email.cz

Table of contents

1. Protected glucuronides **5**

Protected 2-hydroxyphenylacetic acid glucuronide (**perAc-2-HPA-GlcA, 1b**) **5**

Figure S1. HPLC chromatogram for **perAc-2-HPA-GlcA** (**1b**) 5

Table S1. ^{1}H and ^{13}C NMR data for **perAc-2-HPA-GlcA** (**1b**) 5

Figure S2. ^{1}H NMR spectrum of **perAc-2-HPA-GlcA** (**1b**) 6

Figure S3. ^{13}C NMR spectrum of **perAc-2-HPA-GlcA** (**1b**) 6

Figure S4. MS (ESI$^+$) spectrum of **perAc-2-HPA-GlcA** (**1b**) 7

Figure S5. HRMS (ESI$^+$) spectrum of **perAc-2-HPA-GlcA** (**1b**) 7

Protected 3-hydroxyphenylacetic acid glucuronide (**perAc-3-HPA-GlcA, 2b**) **8**

Figure S6. HPLC chromatogram for **perAc-3-HPA-GlcA** (**2b**) 8

Table S2. ^{1}H and ^{13}C NMR data for **perAc-3-HPA-GlcA** (**2b**) 8

Figure S7. ^{1}H NMR spectrum of **perAc-3-HPA-GlcA** (**2b**) 9

Figure S8. ^{13}C NMR spectrum of **perAc-3-HPA-GlcA** (**2b**) 9

Figure S9. MS (ESI$^+$) spectrum of **perAc-3-HPA-GlcA** (**2b**) 10

Figure S10. HRMS (ESI$^+$) spectrum of **perAc-3-HPA-GlcA** (**2b**) 10

Protected 4-hydroxyphenylacetic acid (4-HPA)-glucuronide (**perAc-4-HPA-GlcA, 3b**) **11**

Figure S11. HPLC chromatogram for **perAc-4-HPA-GlcA** (**3b**) 11

Table S3. ^{1}H and ^{13}C NMR data for **perAc-4-HPA-GlcA** (**3b**) 11

Figure S12. ^{1}H NMR spectrum of **perAc-4-HPA-GlcA** (**3b**) 12

Figure S13. ^{13}C NMR spectrum of **perAc-4-HPA-GlcA** (**3b**) 12

Figure S14. MS (ESI$^+$) spectrum of **perAc-4-HPA-GlcA** (**3b**) 13

Figure S15. HRMS (ESI$^+$) spectrum of **perAc-4-HPA-GlcA** (**3b**) 13

Protected 3-(4-hydroxyphenyl)propionic acid glucuronide (**perAc-4-HPP-GlcA, 4b**) **14**

Figure S16. HPLC chromatogram for **perAc-4-HPP-GlcA** (**4b**) 14

Table S4. ^{1}H and ^{13}C NMR data for **perAc-4-HPP-GlcA** (**4b**) 14

Figure S17. ^{1}H NMR spectrum of **perAc-4-HPP-GlcA** (**4b**) 15

Figure S18. ^{13}C NMR spectrum of **perAc-4-HPP-GlcA** (**4b**) 15

Figure S19. MS (ESI$^+$) spectrum of **perAc-4-HPP-GlcA** (**4b**) 16

Figure S20. HRMS (ESI$^+$) spectrum of **perAc-4-HPP-GlcA** (**4b**) 16

Protected 3,4-dihydroxyphenylacetic acid glucuronide (**perAc-DHPA-GlcA, 5b**) **17**

Figure S21. HPLC chromatogram for **perAc-DHPA-GlcA** (**5b** + **5b′**) 17

Table S5. ^{1}H and ^{13}C NMR data for **perAc-DHPA-3′-GlcA** (**5b**) 17

Table S6. ^{1}H and ^{13}C NMR data for **perAc-DHPA-4′-GlcA** (**5b′**) 18

Figure S23. ^{13}C NMR spectrum of **perAc-DHPA-GlcA** (**5b** + **5b′**) 19
Figure S24. MS (ESI$^+$) spectrum of **perAc-DHPA-GlcA** (**5b** + **5b′**) 20
Figure S25. HRMS (ESI$^+$) spectrum of **perAc-DHPA-GlcA** (**5b** + **5b′**) 20
Protected 3-(3,4-dihydroxyphenyl)propionic acid glucuronide (**perAc-DHPP-GlcA**, **6b + 6b′**) **21**
Figure S26. HPLC chromatogram for **perAc-DHPP-GlcA** (**6b** + **6b′**) 21
Table S7. ^{1}H and ^{13}C NMR data for **perAc-DHPA-3′-GlcA** (**6b**) 21
Table S8. ^{1}H and ^{13}C NMR data for **perAc-DHPP-4′-GlcA** (**6b′**) 22
Figure S27. ^{1}H NMR spectrum of **perAc-DHPP-GlcA** (**6b** + **6b′**) 23
Figure S28. ^{13}C NMR spectrum of **perAc-DHPP-GlcA** (**6b** + **6b′**) 23
Figure S29. MS (ESI$^+$) spectrum of **perAc-DHPP-GlcA** (**6b** + **6b′**) 24
Figure S30. HRMS (ESI$^+$) spectrum of **perAc-DHPP-GlcA** (**6b** + **6b′**) 24
2. Glucuronides – Free acids 25
2-Hydroxyphenylacetic acid glucuronide (**2-HPA-GlcA**, **1c**) **........ 25**
Figure S31. HPLC chromatogram for **2-HPA-GlcA** (**1c**) 25
Table S9. ^{1}H and ^{13}C NMR data for **2-HPA-GlcA** (**1c**) 25
Figure S33. ^{13}C NMR spectrum of **2-HPA-GlcA** (**1c**) 26
Figure S34. MS (ESI$^-$) spectrum of **2-HPA-GlcA** (**1c**) 27
Figure S35. HRMS (ESI$^-$) spectrum of **2-HPA-GlcA** (**1c**) 27
Figure S36. CD spectrum of **2-HPA-GlcA** (**1c**) 28
3-Hydroxyphenylacetic acid glucuronide (**3-HPA-GlcA**, **2c**) **........ 29**
Figure S37. HPLC chromatogram for **3-HPA-GlcA** (**2c**) 29
Table S10. ^{1}H and ^{13}C NMR data for **3-HPA-GlcA (2c)** 29
Figure S38. ^{1}H NMR spectrum of **3-HPA-GlcA** (**2c**) 30
Figure S39. ^{13}C NMR spectrum of **3-HPA-GlcA** (**2c**) 30
Figure S40. MS (ESI$^-$) spectrum of **3-HPA-GlcA** (**2c**) 31
Figure S41. HRMS (ESI$^-$) spectrum of **3-HPA-GlcA** (**2c**) 31
4-Hydroxyphenylacetic acid glucuronide (**4-HPA-GlcA**, **3c**) **........ 32**
Figure S42. HPLC chromatogram for **4-HPA-GlcA** (**3c**) 32
Table S11. ^{1}H and ^{13}C NMR data for **4-HPA-GlcA** (**3c**) 32
Figure S43. ^{1}H NMR spectrum of **4-HPA-GlcA** (**3c**) 33
Figure S44. ^{13}C NMR spectrum of **4-HPA-GlcA** (**3c**) 33
Figure S45. MS (ESI$^-$) spectrum of **4-HPA-GlcA** (**3c**) 34
Figure S46. HRMS (ESI$^-$) spectrum of **4-HPA-GlcA** (**3c**) 34

Figure S47. CD spectrum of **4-HPA-GlcA** (**3c**) 35

4-Hydroxyphenylpropionic acid glucuronide (**4-HPP-GlcA**, **4c**) **36**

Figure S48. HPLC chromatogram for **4-HPP-GlcA** (**4c**) 36

Table S12. ^{1}H and ^{13}C NMR data for **4-HPP-GlcA** (**4c**) 36

Figure S49. ^{1}H NMR spectrum of **4-HPP-GlcA** (**4c**) 37

Figure S50. ^{13}C NMR spectrum of **4-HPP-GlcA** (**4c**) 37

Figure S51. MS (ESI^{-}) spectrum of **4-HPP-Glc** (**4c**) 38

Figure S52. HRMS (ESI^{-}) spectrum of **4-HPP-GlcA** (**4c**) 38

Figure S53. CD spectrum of **4-HPP-GlcA** (**4c**) 39

3,4-Dihydroxyphenylacetic acid glucuronide (**DHPA-GlcA**, **5c** + **5c′**) **39**

Figure S54. HPLC chromatogram for **DHPA-GlcA** (**5c** + **5c′**) 40

Table S13. ^{1}H and ^{13}C NMR data for **4′-DHPA-GlcA (5c)** 40

Table S14. ^{1}H and ^{13}C NMR data for **3′-DHPA-GlcA** (**5c′**) 40

Figure S55. ^{1}H NMR spectrum of **DHPA-GlcA** (**5c** + **5c′**) 41

Figure S56. ^{13}C NMR spectrum of **DHPA-GlcA** (**5c** + **5c′**) 42

Figure S57. MS (ESI^{-}) spectrum of **DHPA-GlcA** (**5c** + **5c′**) 42

Figure S58. HRMS (ESI^{-}) spectrum of **DHPA-GlcA** (**5c** + **5c′**) 43

Figure S59. CD spectrum of **DHPA-GlcA** (**5c** + **5c′**) 43

3-(3,4-Dihydroxyphenyl)propionic acid glucuronide (**DHPP-GlcA**, **6c** + **6c′**) **44**

Figure S60. HPLC chromatogram for **DHPP-GlcA** (**6c** + **6c′**) 44

Table S15. ^{1}H and ^{13}C NMR data for **DHPP-4′-GlcA** (**6c**) 44

Table S16. ^{1}H and ^{13}C NMR data for **DHPP-3′-GlcA** (**6c′**) 45

Figure S61. ^{1}H NMR spectrum of **DHPP-GlcA** (**6c** + **6c′**) 46

Figure S63. MS (ESI^{-}) spectrum of **DHPP-GlcA** (**6c** + **6c′**) 47

Figure S64. HRMS (ESI^{-}) spectrum of **DHPP-GlcA** (**6c** + **6c′**) 47

Figure S65. CD spectrum of **DHPP-GlcA** (**6c + 6c′**) 48

1. Protected glucuronides

Protected 2-hydroxyphenylacetic acid glucuronide (**perAc-2-HPA-GlcA**, **1b**)

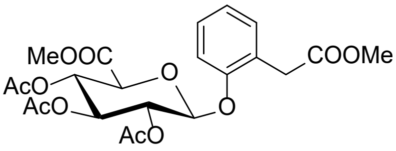

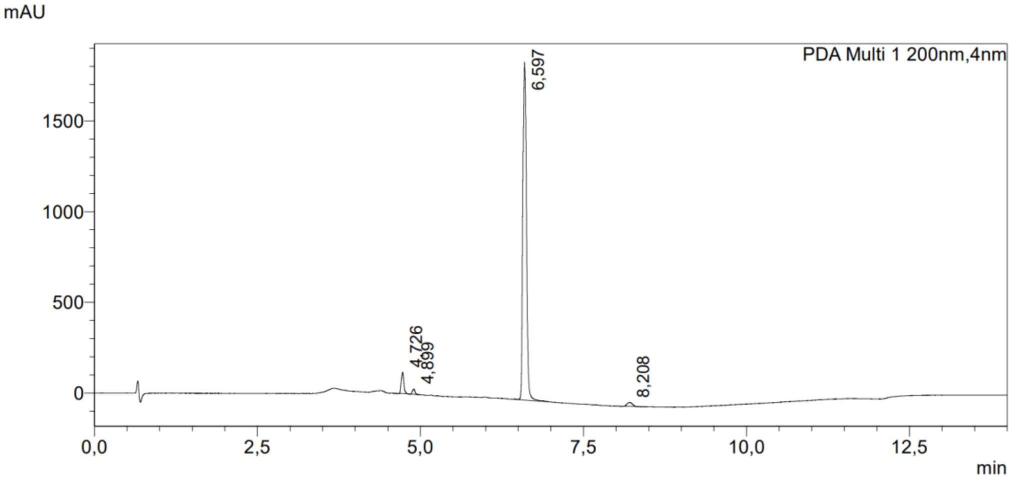

Figure S1. HPLC chromatogram for **perAc-2-HPA-GlcA** (**1b**)
(RT= 6.597 min, 94% purity)

Table S1. ^{1}H and ^{13}C NMR data for **perAc-2-HPA-GlcA** (**1b**)
700.13 MHz for ^{1}H, 176.05 MHz for ^{13}C, $CDCl_3$, 20 °C

Atom	δ_C	m.	δ_H	n_H	m.	*J* [Hz]	diagnostic HMBC
**1**	98.83	d	5.167	1	d	7.1	5
**2**	70.77	d	5.298	1	m	-	Ac
**2-CO**	169.29	s	-	0	-	-	2, Ac
**Ac**	20.67	q	2.077	3	s	-	
**3**	71.84	d	5.33^{H}	1	m	-	Ac
**3-CO**	170.11	s	-	0	-	-	3, Ac
**Ac**	20.63	q	2.056	3	s	-	
**4**	69.05	d	5.34^{H}	1	m	-	Ac
**4-CO**	169.37	s	-	0	-	-	4, Ac
**Ac**	20.53	q	2.048	3	s	-	
**5**	72.51	d	4.177	1	m	-	1, 5-OMe
**5-CO**	166.85	s	-	0	-	-	5, 5-OMe
**5-OMe**	52.98	q	3.727	3	s	-	
**1′**	154.54	s	-	0	-	-	1, 1′′, 3′, 5′
**2′**	124.30	s	-	0	-	-	1′′, 4′, 6′
**3′**	131.19	d	7.209	1	dd	7.5, 1.4	1′′, 5′
**4′**	123.51	d	7.056	1	br dd	-	6′

**5′**	128.72	d	7.257	1	br ddd	Σ*J* = 17.4	3′
**6′**	115.29	d	7.037	1	br d	-	4′
**1″**	34.98	t	3.722	1	d	16.4	3′
			3.528	1	d	16.4	
**1″-CO**	171.67	s	-	0	-	-	1″, 1″-OMe
**1″-OMe**	51.92	q	3.687	3	s	-	

[H] … HSQC readout

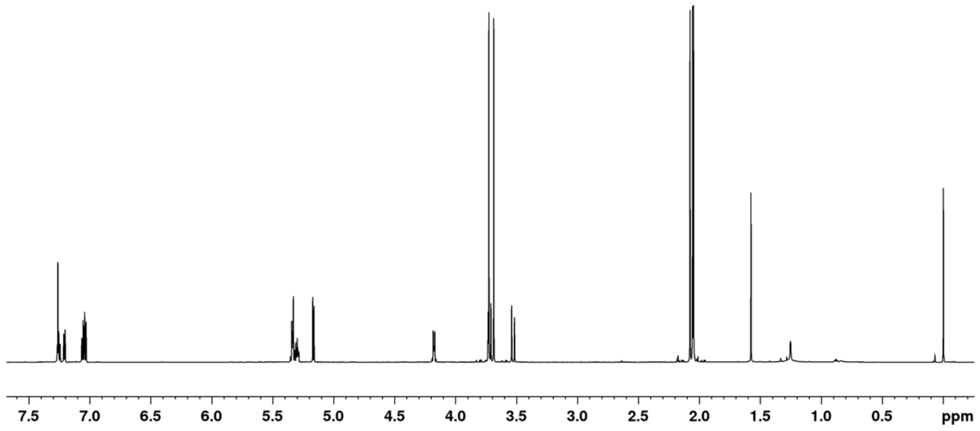

Figure S2. ^{1}H NMR spectrum of **perAc-2-HPA-GlcA** (**1b**)
(700.13 MHz, $CDCl_3$, 20 °C)

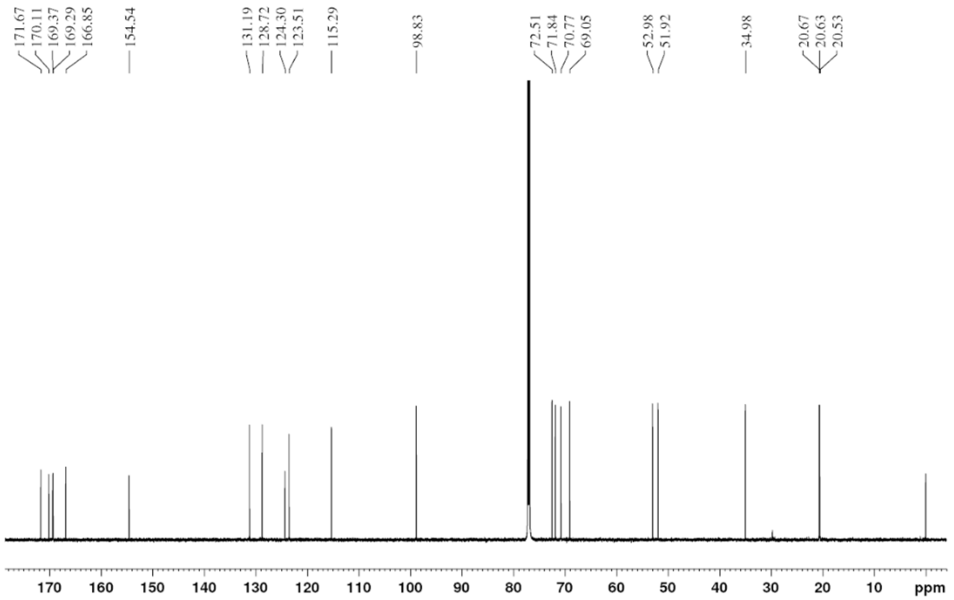

Figure S3. ^{13}C NMR spectrum of **perAc-2-HPA-GlcA** (**1b**)
(176.05 MHz, $CDCl_3$, 20 °C)

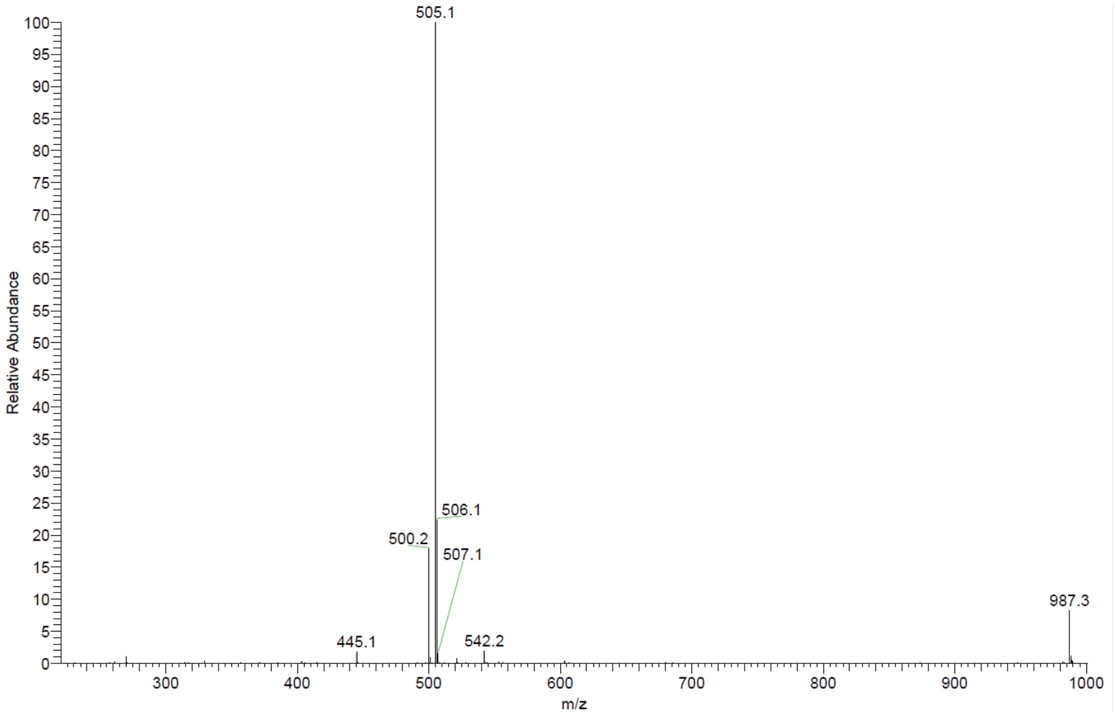

Figure S4. MS (ESI$^+$) spectrum of **perAc-2-HPA-GlcA** (**1b**)
([M+ Na]$^+$, *m/z* 505.1)

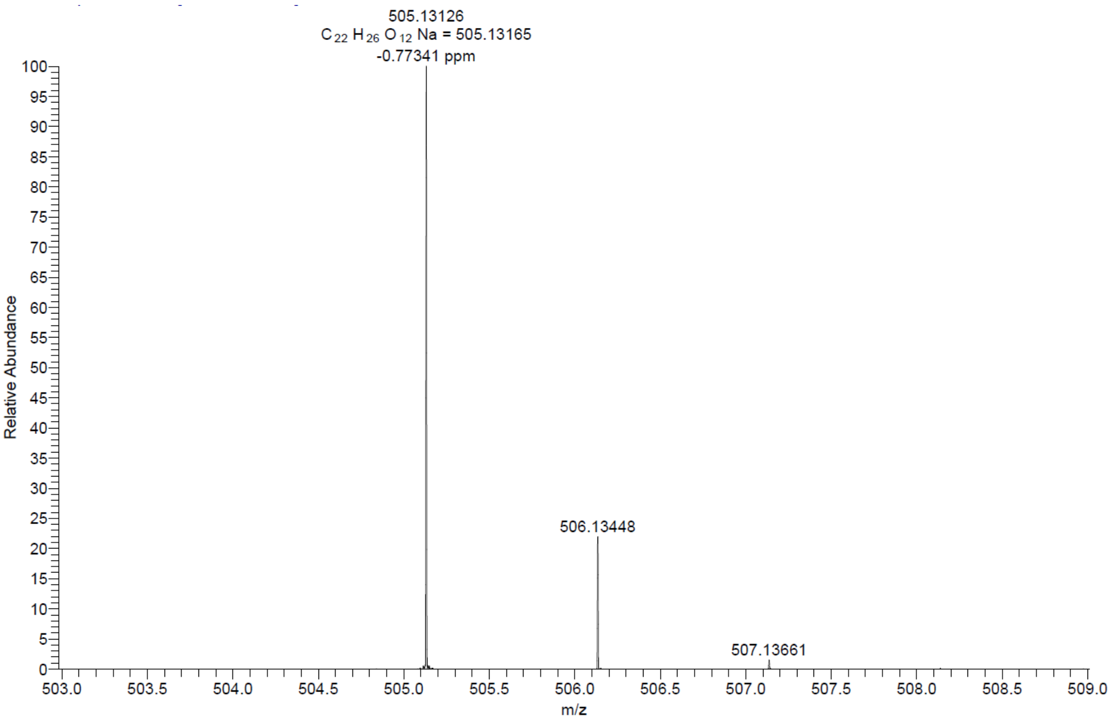

Figure S5. HRMS (ESI$^+$) spectrum of **perAc-2-HPA-GlcA** (**1b**)
Calculated (for $C_{22}H_{26}O_{12}Na$) 505.13165, measured 505.13126 (-0.8 ppm)

Protected 3-hydroxyphenylacetic acid glucuronide (perAc-3-HPA-GlcA, 2b)

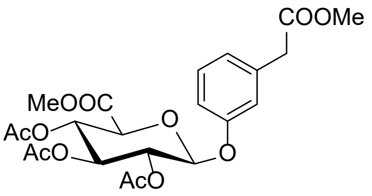

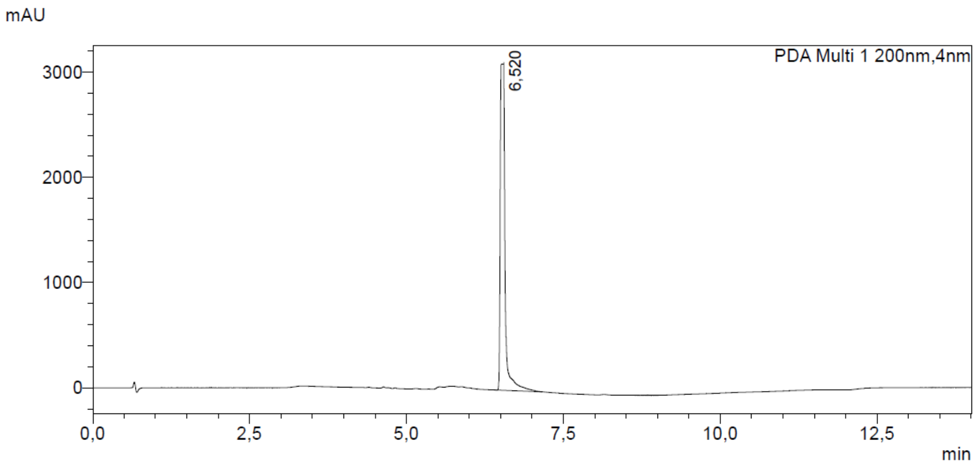

Figure S6. HPLC chromatogram for **perAc-3-HPA-GlcA (2b)** (RT= 6.520 min, 99% purity)

Table S2. ^{1}H and ^{13}C NMR data for **perAc-3-HPA-GlcA (2b)** (700.13 MHz for ^{1}H, 176.05 MHz for ^{13}C, $CDCl_3$, 20 °C)

Atom	δ_C	m.	δ_H	n_H	m.	*J* [Hz]	diagnostic HMBC
**1**	98.92	d	5.155	1	d	7.4	5
**2**	70.99	d	5.276	1	m	-	
**2-CO**	169.23	s	-	0	-	-	2, Ac
**Ac**	20.62[a]	q	2.061	3	s	-	
**3**	71.80	d	5.338	1	m	-	Ac
**3-CO**	170.10	s	-	0	-	-	3, Ac
**Ac**	20.61[a]	q	2.047	3	s	-	
**4**	69.05	d	5.338	1	m	-	Ac
**4-CO**	169.35	s	-	0	-	-	4, Ac
**Ac**	20.50	q	2.042	3	s	-	
**5**	72.60	d	4.185	1	m	-	1, OMe
**CO**	166.85	s	-	0	-	-	5, OMe
**OMe**	52.96	q	3.723	3	s	-	
**1′**	156.68	s	-	0	-	-	1, 5′
**2′**	118.09	d	6.91[H]	1	m	-	CH_2, 4′, 5′
**3′**	135.63	s	-	0	-	-	CH_2, 5′
**4′**	124.43	d	6.991	1	br d	ΣJ = 15.6	CH_2
**5′**	129.70	d	7.249	1	dm	7.7	

**6′**	115.48	d	6.90[H]	1	m	-	2′, 4′, 5′
**CH_2**	40.93	t	3.594	2	s	-	CH_2
**CO**	171.60	s	-	0	-	-	CH_2, OMe
**OMe**	52.10	q	3.689	3	s	-	

[a] … might be interchanged; [H] … HSQC readout

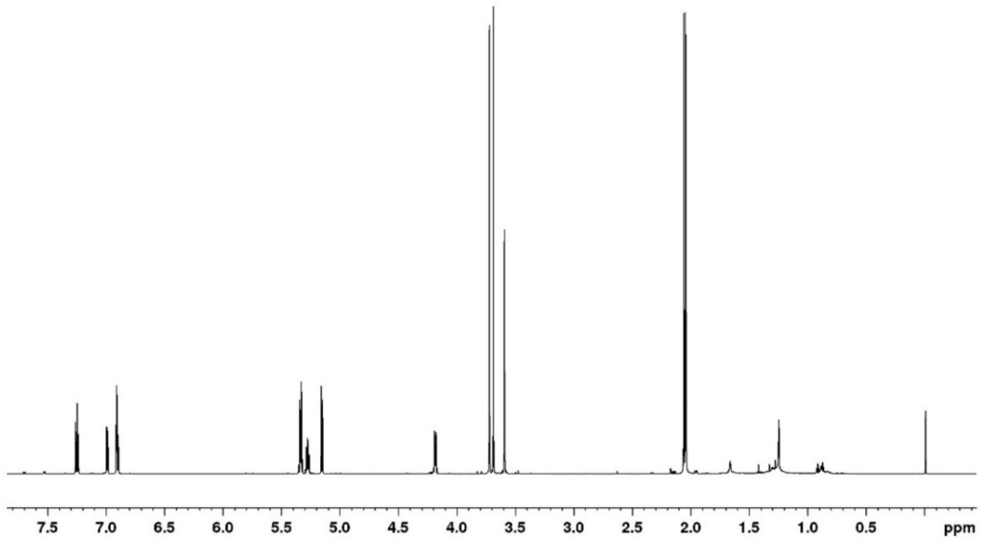

Figure S7. ^{1}H NMR spectrum of **perAc-3-HPA-GlcA** (**2b**)
(700.13 MHz, $CDCl_3$, 20 °C)

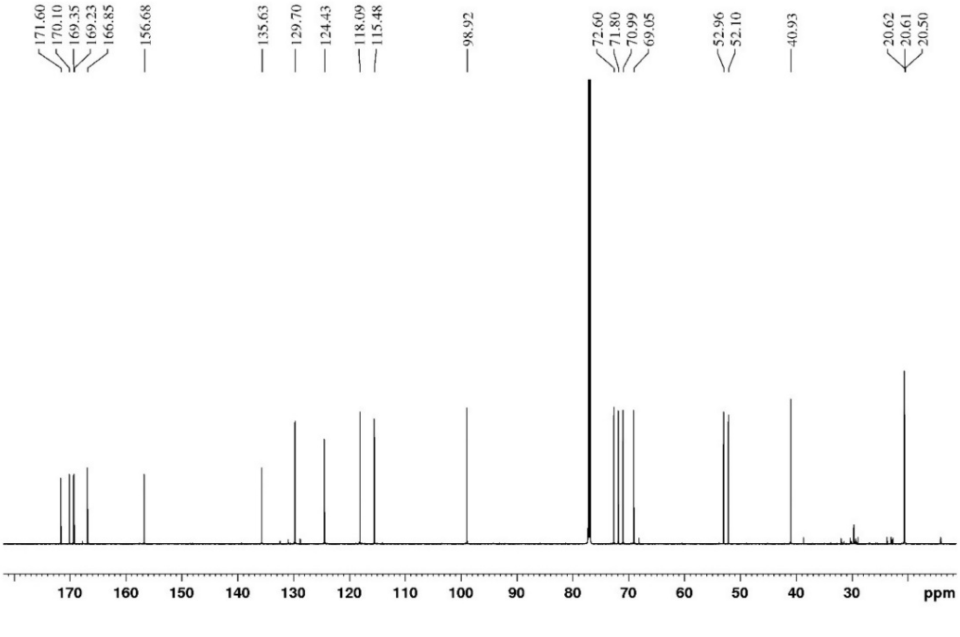

Figure S8. ^{13}C NMR spectrum of **perAc-3-HPA-GlcA** (**2b**)
(176.05 MHz, $CDCl_3$, 20 °C)

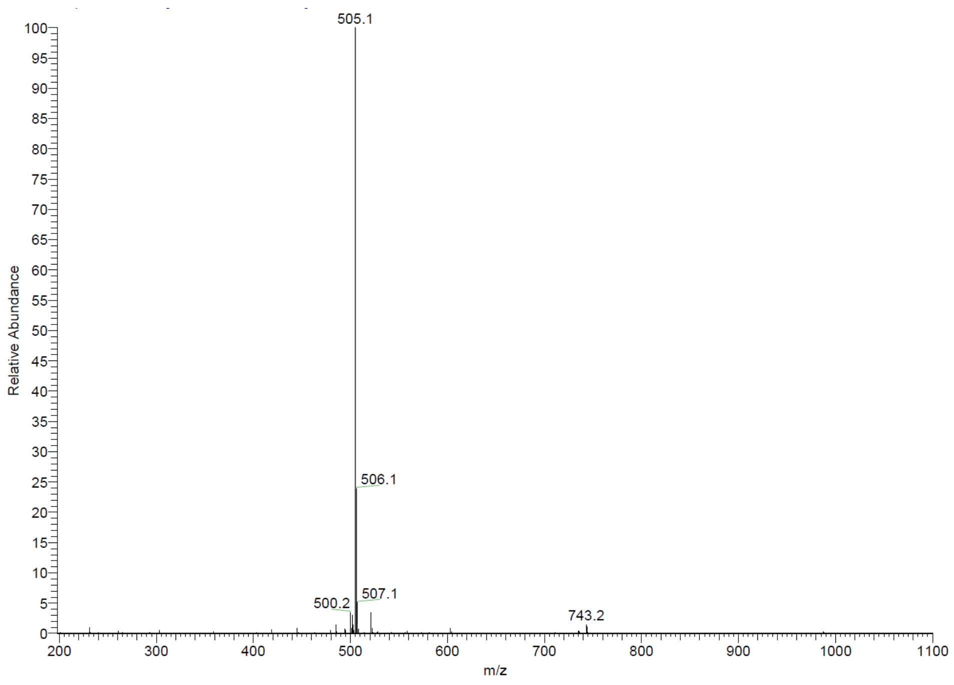

Figure S9. MS (ESI$^+$) spectrum of **perAc-3-HPA-GlcA** (**2b**)
([M+ Na]$^+$, *m/z* 505.1)

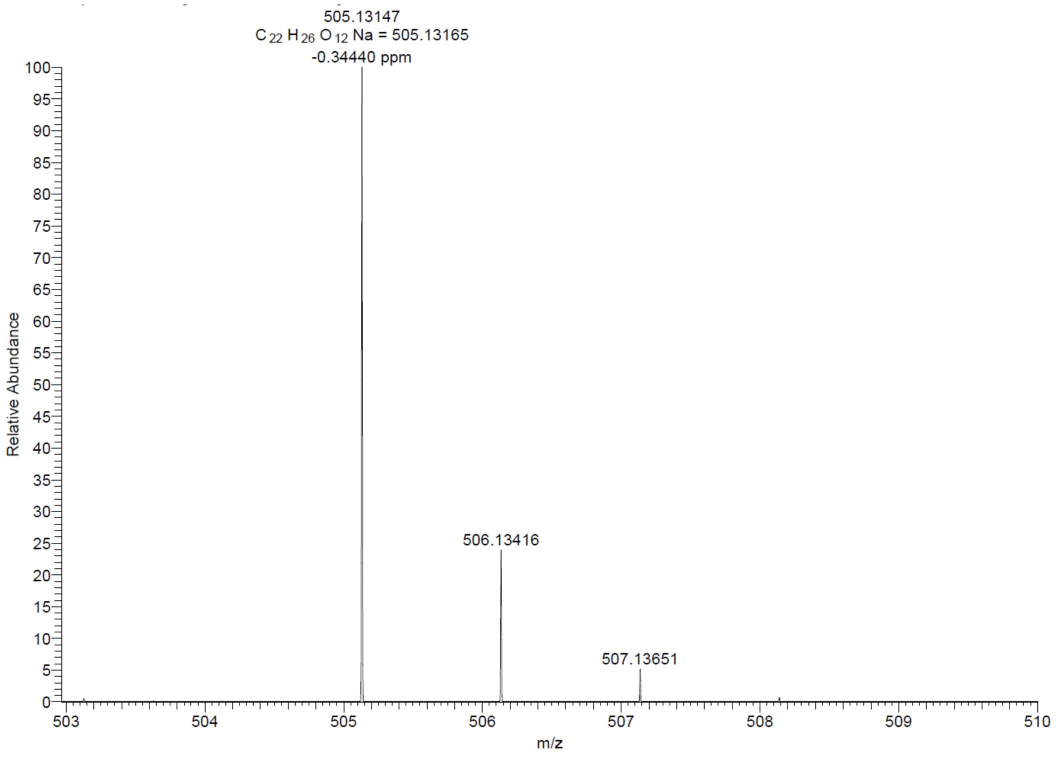

Figure S10. HRMS (ESI$^+$) spectrum of **perAc-3-HPA-GlcA** (**2b**)
Calculated (for $C_{22}H_{26}O_{12}Na$) 505.13165, measured 505.13147 (-0.3 ppm)

Protected 4-hydroxyphenylacetic acid (4-HPA)-glucuronide (perAc-4-HPA-GlcA, 3b)

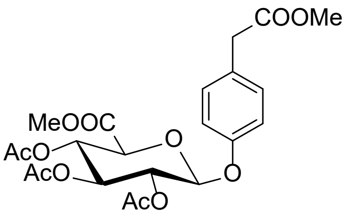

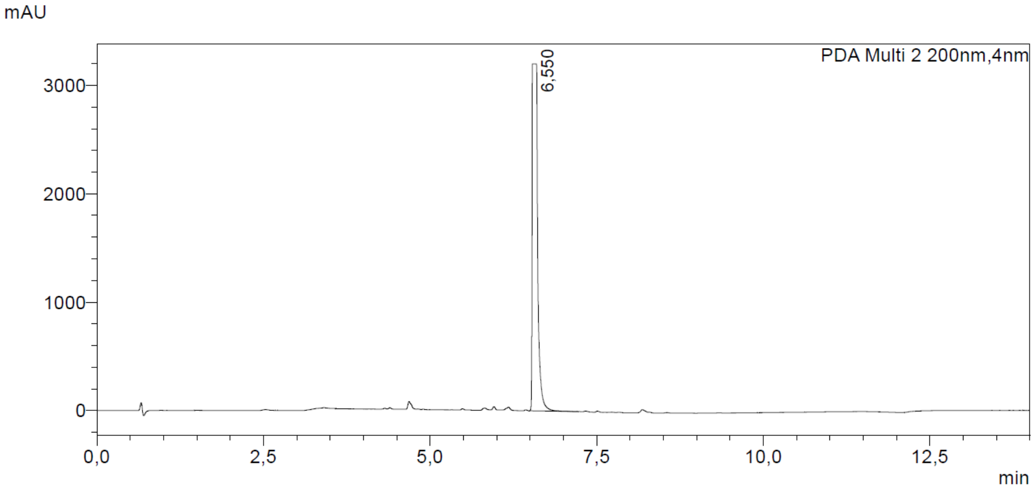

Figure S11. HPLC chromatogram for **perAc-4-HPA-GlcA** (**3b**)
(RT= 6.550 min, 99% purity)

Table S3. ^{1}H and ^{13}C NMR data for **perAc-4-HPA-GlcA** (**3b**)
(700.13 MHz for ^{1}H, 176.05 MHz for ^{13}C, $CDCl_3$, 20 °C)

Atom	δ_C	m.	δ_H	n_H	m.	*J* [Hz]	diagnostic HMBC
**1**	99.12	d	5.118	1	d	7.5	5
**2**	70.97	d	5.271	1	m	-	
**2-CO**	169.22	s	-	0	-	-	2, Ac
**Ac**	20.61^{a}	q	2.053	3	s	-	
**3**	71.81	d	5.332	1	m	-	Ac
**3-CO**	170.11	s	-	0	-	-	3, Ac
**Ac**	20.60^{a}	q	2.045	3	s	-	
**4**	69.07	d	5.332	1	m	-	Ac
**4-CO**	169.34	s	-	0	-	-	4, Ac
**Ac**	20.50	q	2.040	3	s	-	
**5**	72.60	d	4.166	1	m	-	1, OMe
**CO**	166.83	s	-	0	-	-	5, OMe
**OMe**	52.98	q	3.726	3	s	-	
ipso-	155.78	s	-	0	-	-	1, *o-*, *m-*
ortho-	117.15^{x}	d	6.948	2	m	Σ*J* = 8.7	
meta-	130.47^{x}	d	7.201	2	m	Σ*J* = 8.7	
para-	129.07	s	-	0	-	-	CH_2, *o-*

**CH_2**	40.26	t	3.576	2	s	-	CH_2
**CO**	172.03	s	-	0	-	-	CH_2, OMe
**OMe**	52.07	q	3.682	3	s	-	

[a] … might be interchanged; [x] … 2C

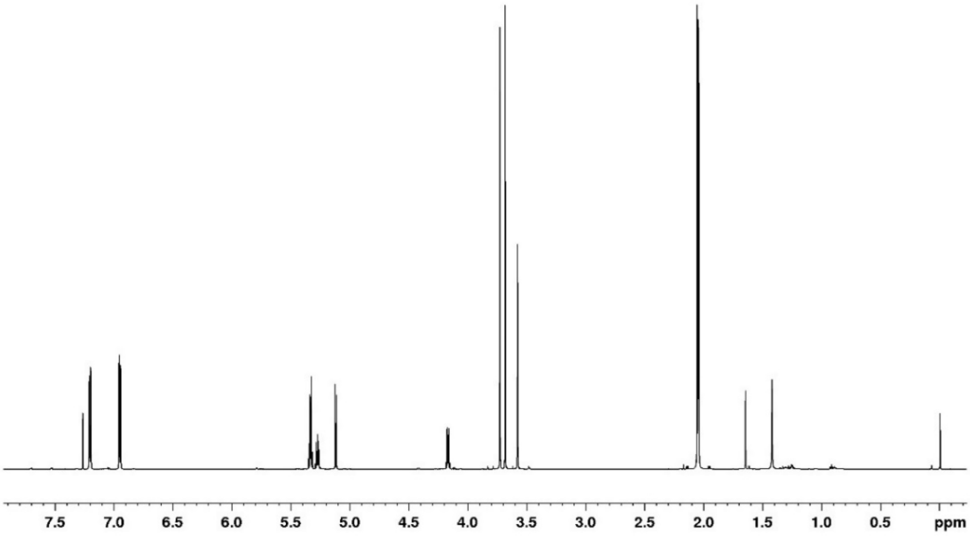

Figure S12. ^1H NMR spectrum of **perAc-4-HPA-GlcA** (**3b**)
(700.13 MHz, $CDCl_3$, 20 °C)

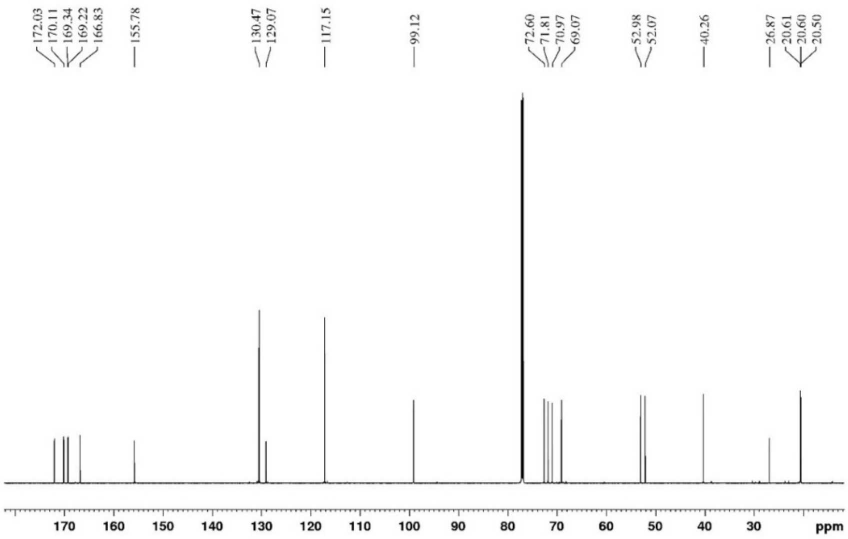

Figure S13. ^{13}C NMR spectrum of **perAc-4-HPA-GlcA** (**3b**)
(176.05 MHz, $CDCl_3$, 20 °C)

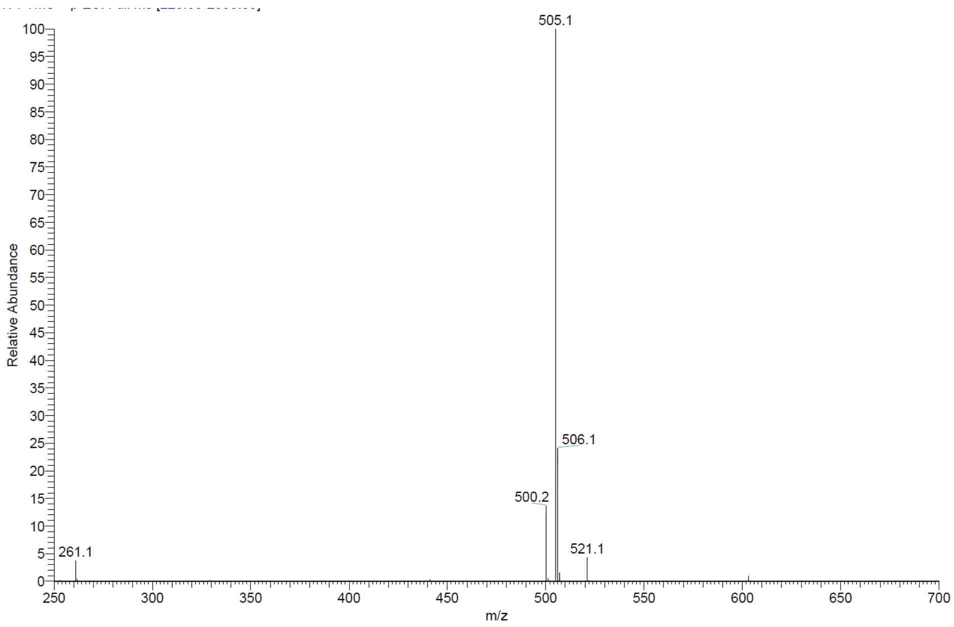

Figure S14. MS (ESI$^+$) spectrum of **perAc-4-HPA-GlcA** (**3b**)
([M+ Na]$^+$, *m/z* 505.1)

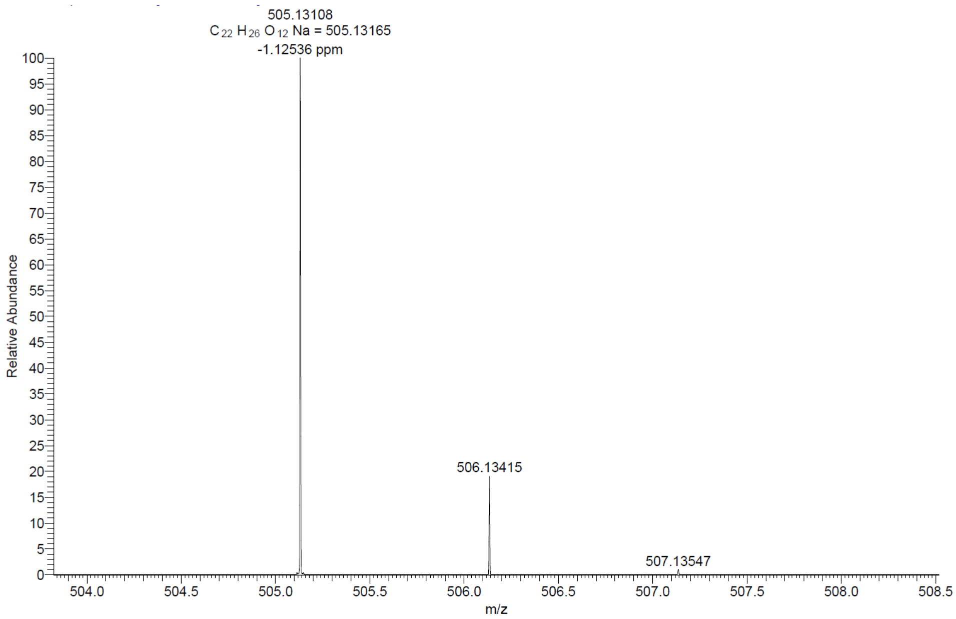

Figure S15. HRMS (ESI$^+$) spectrum of **perAc-4-HPA-GlcA** (**3b**)
Calculated (for $C_{22}H_{26}O_{12}Na$) 505.13165, measured 505.13108 (-1.1 ppm)

Protected 3-(4-hydroxyphenyl)propionic acid glucuronide (perAc-4-HPP-GlcA, 4b)

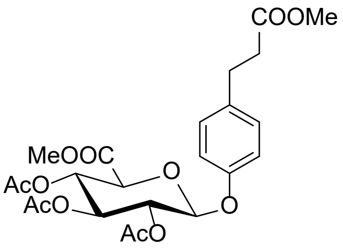

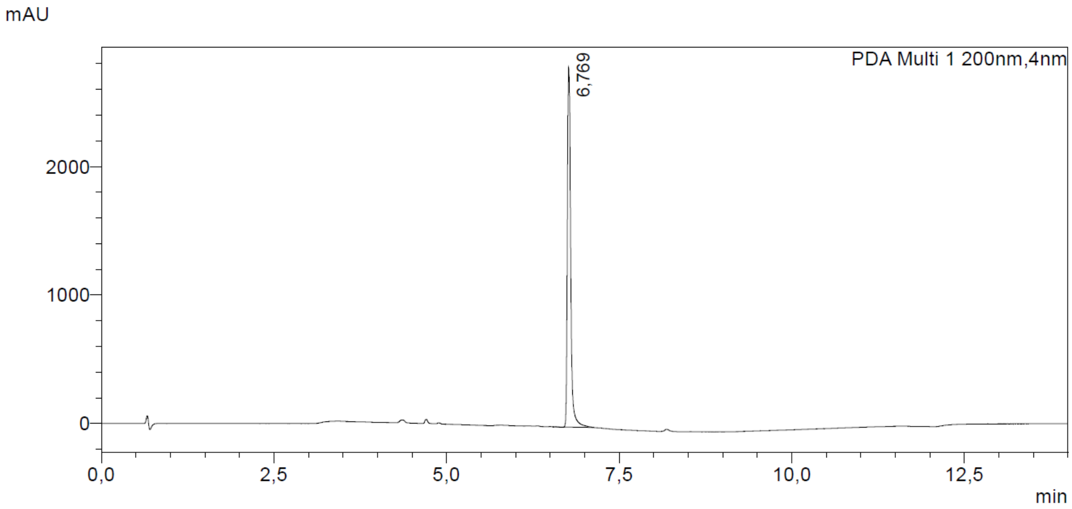

Figure S16. HPLC chromatogram for **perAc-4-HPP-GlcA** (**4b**)
(RT= 6.769 min, 99% purity)

Table S4. ^{1}H and ^{13}C NMR data for **perAc-4-HPP-GlcA** (**4b**)
(600.23 MHz for ^{1}H, 150.93 MHz for ^{13}C, $CDCl_3$, 20 °C)

Atom	δ_C	m.	δ_H	n_H	m.	*J* [Hz]	diagnostic HMBC
**1**	99.34	d	5.100	1	d	7.4	5
**2**	71.04	d	5.268	1	m	-	
**2-CO**	169.24	s	-	0	-	-	2, Ac
**Ac**	20.63	q	2.060	3	s	-	
**3**	71.88	d	5.334	1	m	-	Ac
**3-CO**	170.14	s	-	0	-	-	Ac
**Ac**	20.63	q	2.049	3	s	-	
**4**	69.13	d	5.334	1	m	-	Ac
**4-CO**	169.35	s	-	0	-	-	Ac
**Ac**	20.52	q	2.043	3	s	-	
**5**	72.65	d	4.159	1	m	-	1, OMe
**CO**	166.88	s	-	0	-	-	5, OMe
**OMe**	52.98	q	3.732	3	s	-	
ipso-	155.21	s	-	0	-	-	1, *o-*, *m-*

ortho-	117.19[x]	d	6.918	2	m	Σ*J* = 8.7	
meta-	129.41[x]	d	7.123	2	m	Σ*J* = 8.7	1′
para-	135.71	s	-	0	-	-	1′, 2′, *o-*
**1′**	30.10	t	2.902	2	t	7.7	2′
**2′**	35.75	t	2.597	2	t	7.7	1′, OMe
**CO**	173.24	s	-	0	-	-	1′, 2′, OMe
**OMe**	51.65	q	3.665	3	s	-	

[x] … 2C

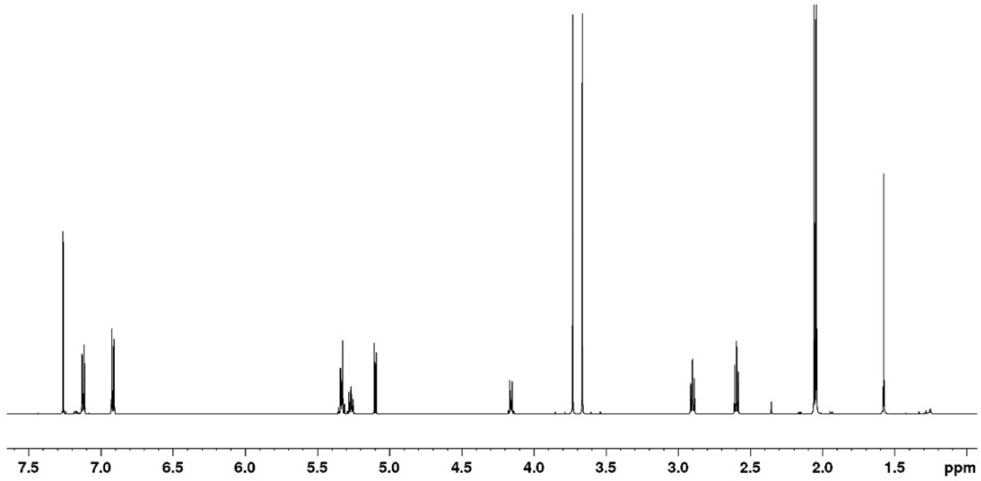

Figure S17. ^{1}H NMR spectrum of **perAc-4-HPP-GlcA** (**4b**)
(600.23 MHz, $CDCl_3$, 20 °C)

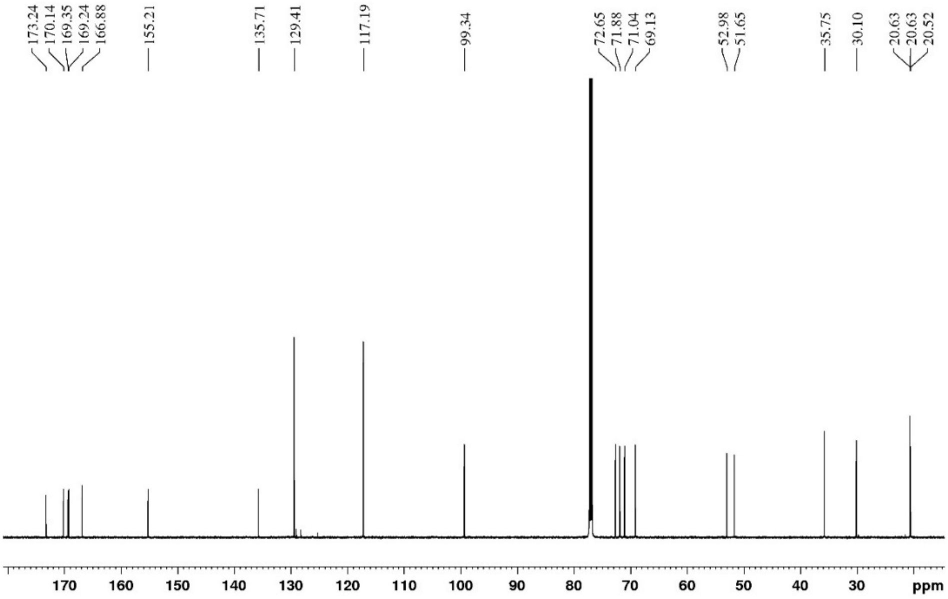

Figure S18. ^{13}C NMR spectrum of **perAc-4-HPP-GlcA** (**4b**)
(150.93 MHz, $CDCl_3$, 20 °C)

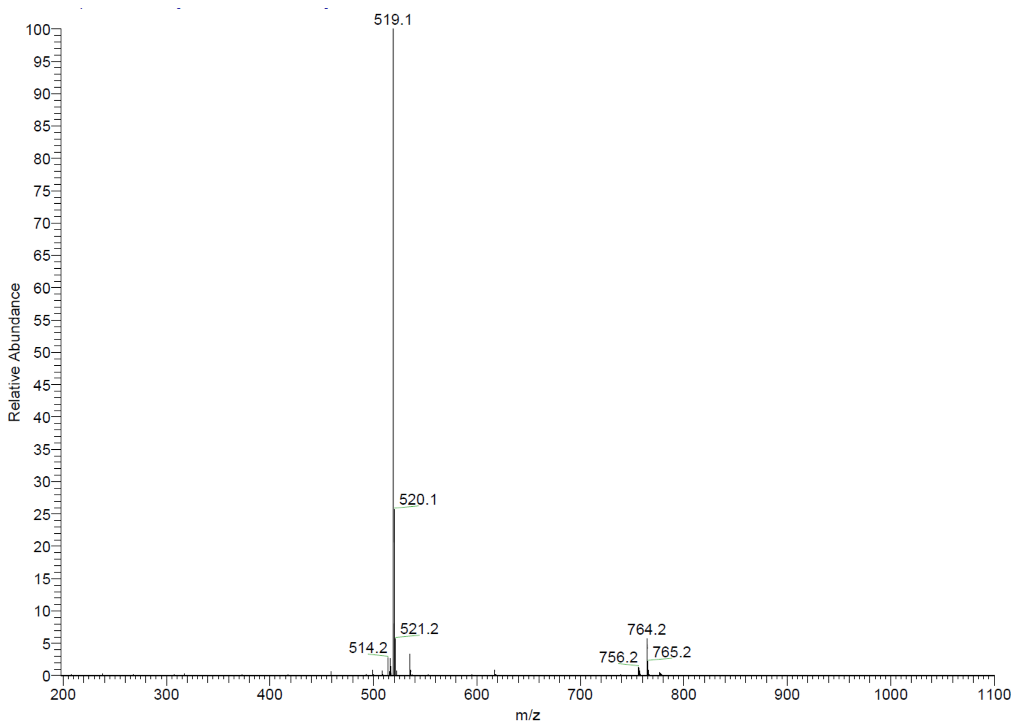

Figure S19. MS (ESI$^+$) spectrum of **perAc-4-HPP-GlcA** (**4b**)
([M+ Na]$^+$, *m/z* 519.1)

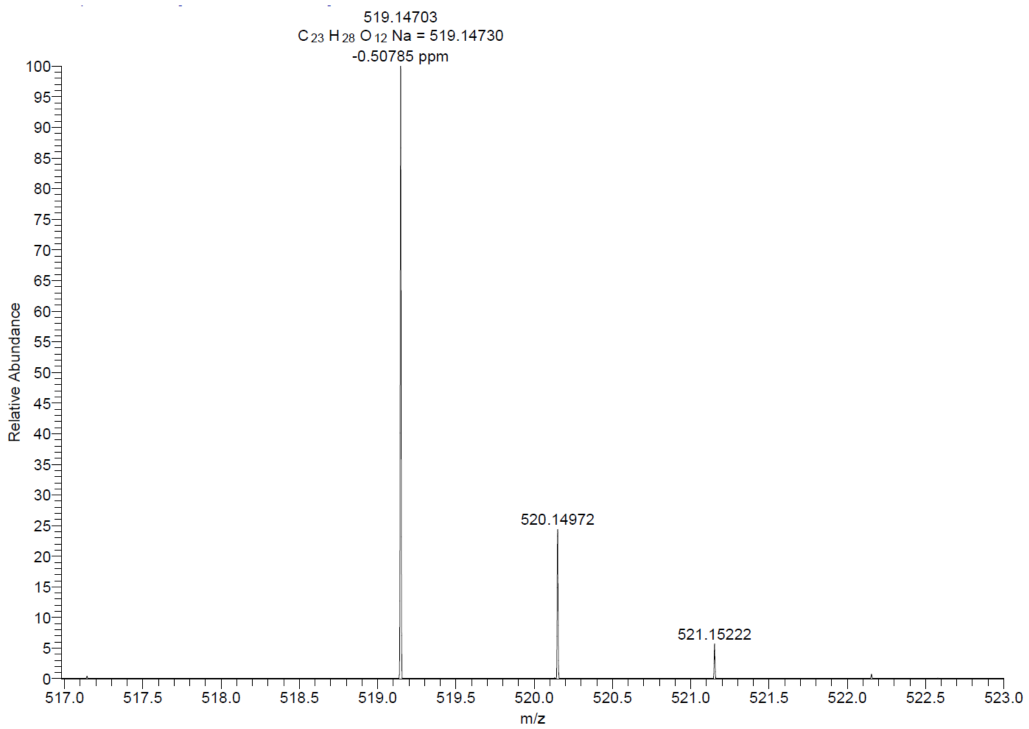

Figure S20. HRMS (ESI$^+$) spectrum of **perAc-4-HPP-GlcA** (**4b**)
Calculated (for $C_{23}H_{28}O_{12}Na$) 519.14730, measured 519.14703 (-0.5 ppm).

Protected 3,4-dihydroxyphenylacetic acid glucuronide (perAc-DHPA-GlcA, 5b)

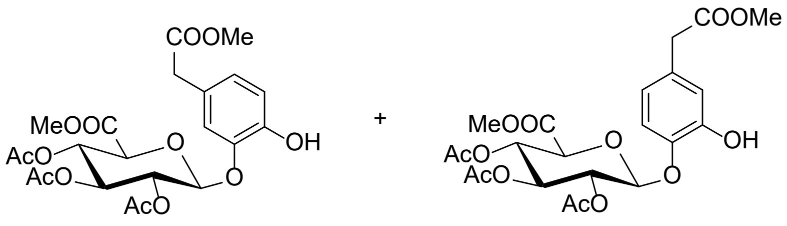

protected-DHPA-3'-Glc *protected*-DHPA-4'-Glc

Approximate molar ratio of 3′:4′ isomer = 40:60

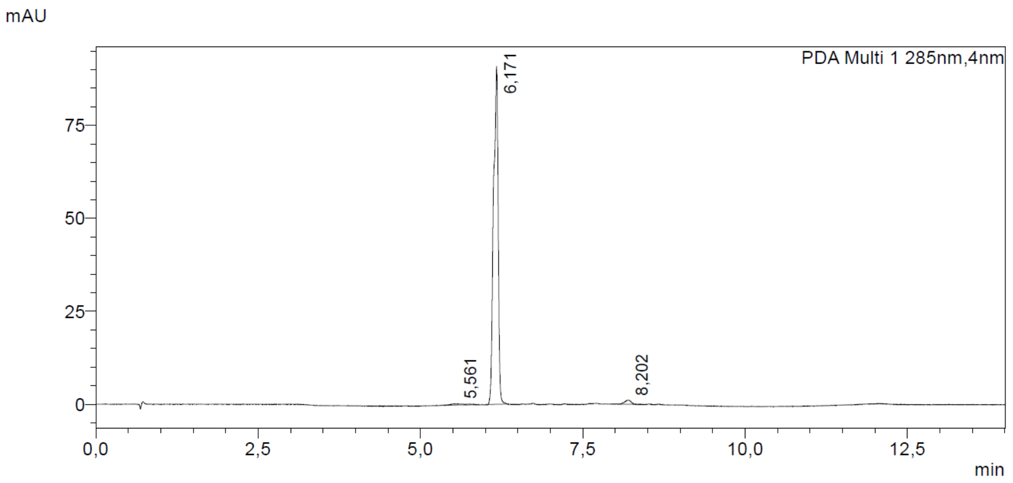

Figure S21. HPLC chromatogram for **perAc-DHPA-GlcA** (**5b** + **5b′**)
(RT= 6.171 min, 97% purity)

Table S5. ^{1}H and ^{13}C NMR data for **perAc-DHPA-3′-GlcA** (**5b**)
(700.13 MHz for ^{1}H, 176.05 MHz for ^{13}C, $CDCl_3$, 20 °C)

Atom	δ_C	m.	δ_H	n_H	m.	*J* [Hz]	diagnostic HMBC
**1**	101.39	d	5.028	1	d	7.6	5
**2**	71.03	d	5.281	1	dd	9.3, 7.6	
**2-CO**	169.83	s	-	0	-	-	2, Ac
**Ac**	20.70	q	2.114	3	s	-	
**3**	71.26	d	5.355	1	dd	9.3, 9.2	Ac
**3-CO**	169.93	s	-	0	-	-	3, Ac
**Ac**	20.58	q	2.058	3	s	-	
**4**	68.93	d	5.309	1	dd	9.4, 9.2	Ac
**4-CO**	169.41	s	-	0	-	-	4, Ac
**Ac**	20.47	q	2.052	3	s	-	
**5**	72.45	d	4.178	1	d	9.4	1, OMe
**5-CO**	166.70	s	-	0	-	-	5, OMe
**OMe**	52.08	q	3.764	3	s	-	
**1′**	125.99	s	-	0	-	-	1″
**2′**	118.82	d	6.890	1	d	2.1	

**3′**	143.80	s	-	0	-	-	1, 2′, 5′,
**4′**	146.65	s	-	0	-	-	OH, 2′, 5′, 6′
**5′**	116.56	d	6.898	1	d	8.3	OH
**6′**	126.34	d	6.929	1	dd	8.3, 2.1	
**4′-OH**	-	-	6.030	1	s	-	
**1″**	40.24	t	3.526	2	s	-	2′, 6′
**1″-CO**	172.05	s	-	0	-	-	1″, OMe
**OMe**	52.08	q	3.689	3	s	-	

Table S6. ^{1}H and ^{13}C NMR data for **perAc-DHPA-4′-GlcA** (**5b′**)
(700.13 MHz for ^{1}H, 176.05 MHz for ^{13}C, $CDCl_3$, 20 °C)

Atom	δ_C	m.	δ_H	n_H	m.	*J* [Hz]	diagnostic HMBC
**1**	101.45	d	4.989	1	d	7.6	5
**2**	71.04	d	5.274	1	dd	9.3, 7.6	
**2-CO**	169.81	s	-	0	-	-	2, Ac
**Ac**	20.69	q	2.104	3	s	-	
**3**	71.29	d	5.350	1	dd	9.3, 9.2	Ac
**3-CO**	169.95	s	-	0	-	-	3, Ac
**Ac**	20.58	q	2.056	3	s	-	
**4**	68.93	d	5.307	1	dd	9.4, 9.2	Ac
**4-CO**	169.39	s	-	0	-	-	4, Ac
**Ac**	20.47	q	2.048	3	s	-	
**5**	72.46	d	4.154	1	d	9.4	1, OMe
**5-CO**	166.67	s	-	0	-	-	5, OMe
**OMe**	53.16	q	3.761	3	s	-	
**1′**	131.37	s	-	0	-	-	1″
**2′**	117.47	d	6.878	1	d	2.1	OH
**3′**	147.41	s	-	0	-	-	OH, 2′, 5′
**4′**	143.15	s	-	0	-	-	1, 2′, 5′, 6′
**5′**	117.92	d	6.906	1	d	8.2	
**6′**	117.92	d	6.906	1	d	8.2	
**3′-OH**	-	-	6.140	1	s	-	
**1″**	40.55	t	3.534	2	s	-	2′, 6′
**1″-CO**	171.86	s	-	0	-	-	1″, OMe
**OMe**	52.10	q	3.683	3	s	-	

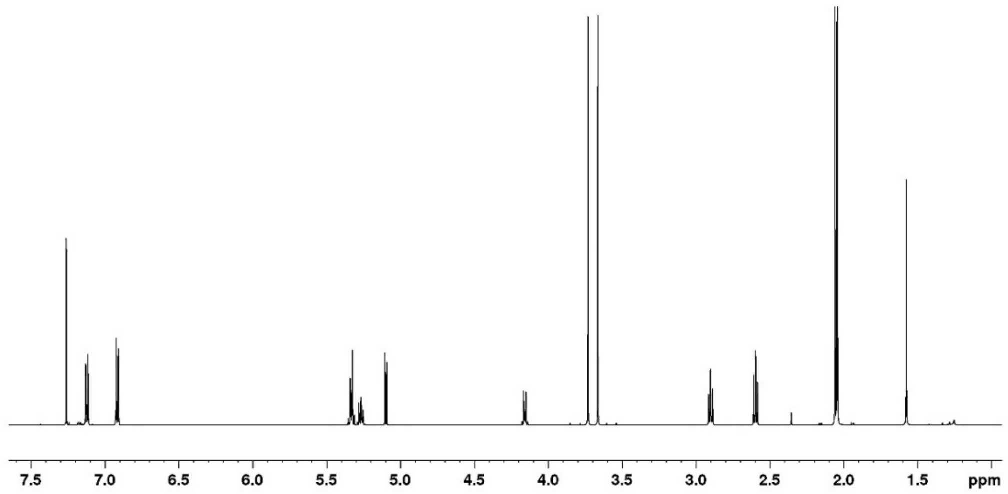

Figure S22. ^{1}H NMR spectrum of **perAc-DHPA-GlcA** (**5b** + **5b'**)
(700.13 MHz, $CDCl_3$, 20 °C)

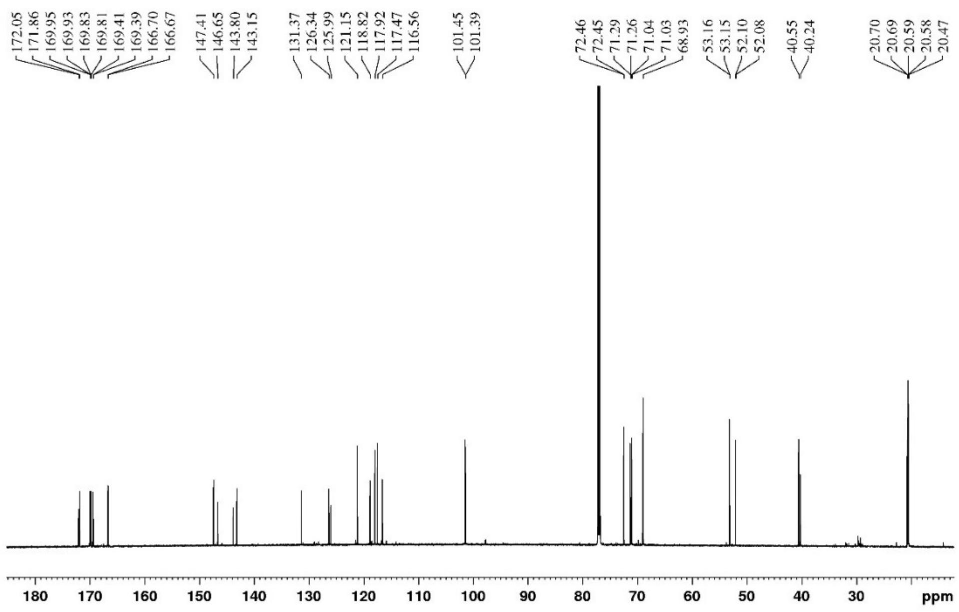

Figure S23. ^{13}C NMR spectrum of **perAc-DHPA-GlcA** (**5b** + **5b'**)
(176.05 MHz, $CDCl_3$, 20 °C)

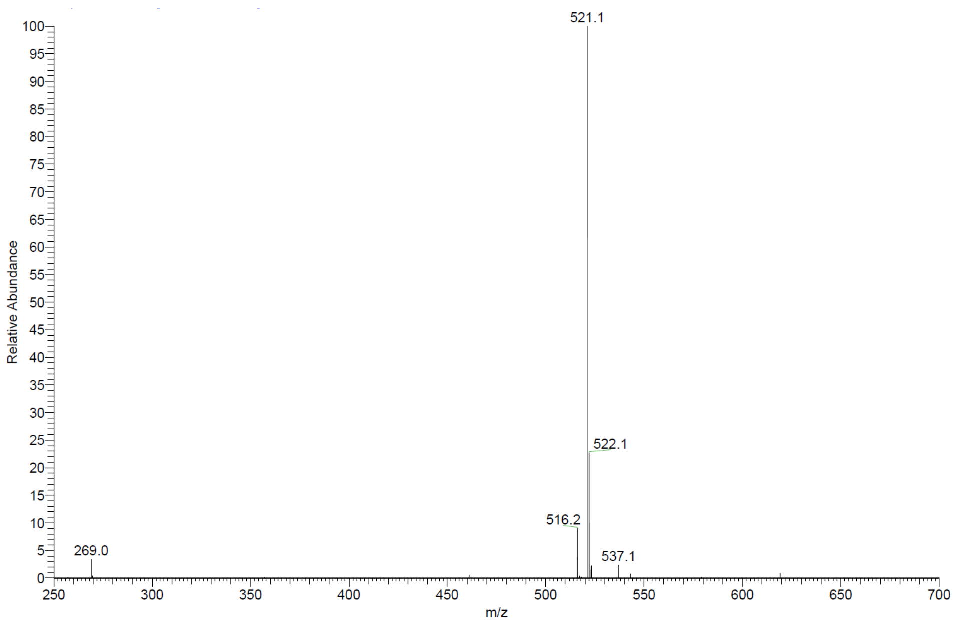

Figure S24. MS (ESI$^+$) spectrum of **perAc-DHPA-GlcA** (**5b** + **5b'**)
([M+ Na]$^+$, *m/z* 521.1)

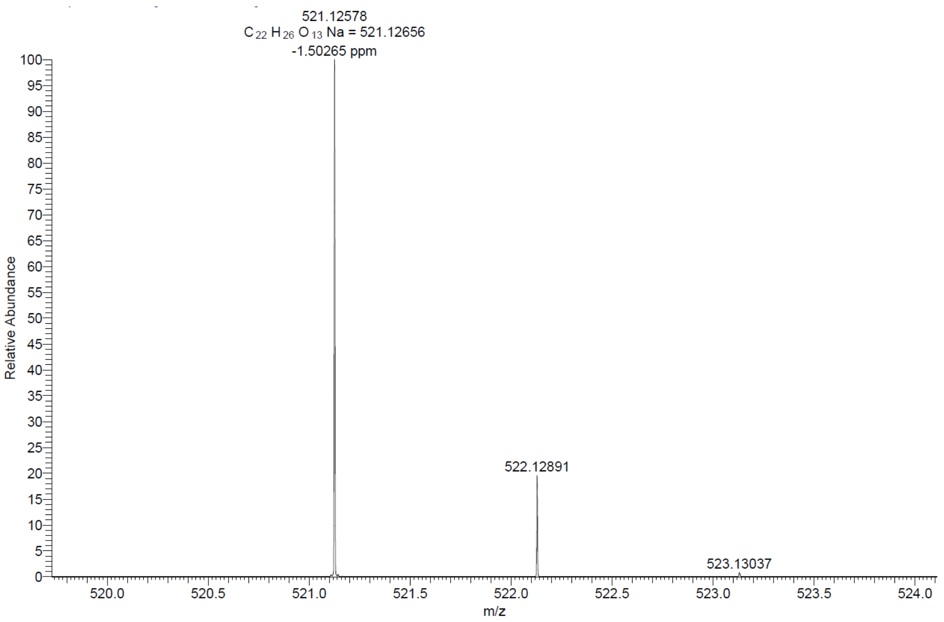

Figure S25. HRMS (ESI$^+$) spectrum of **perAc-DHPA-GlcA** (**5b** + **5b'**)
Calculated (for $C_{22}H_{26}O_{13}Na$) 521.12656, measured 521.12578 (-1.5 ppm)

Protected 3-(3,4-dihydroxyphenyl)propionic acid glucuronide (perAc-DHPP-GlcA, 6b + 6b′)

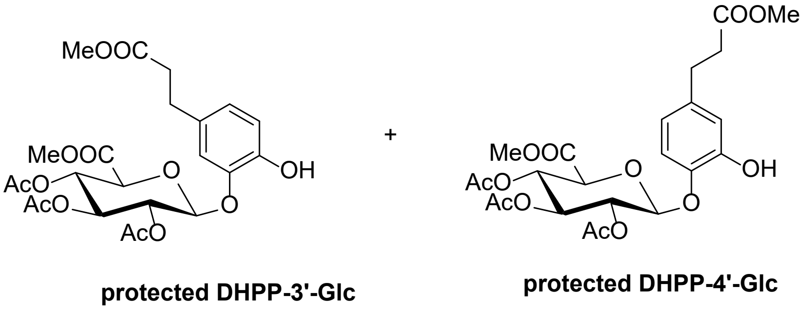

Approximate molar ratio of 3′:4′ isomer = 45:55

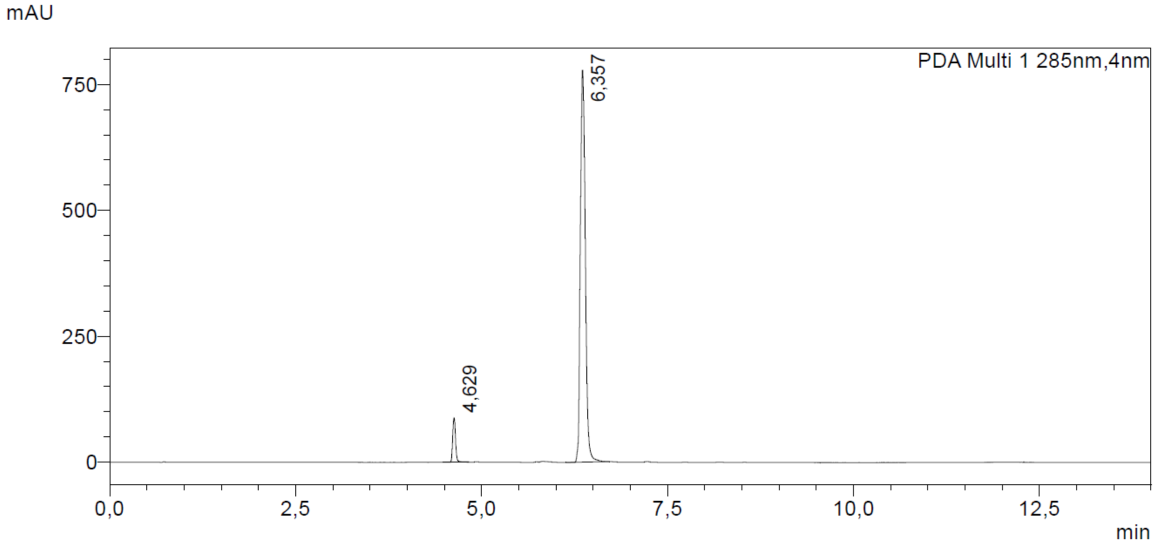

Figure S26. HPLC chromatogram for **perAc-DHPP-GlcA** (**6b** + **6b′**)
(RT= 6.357 min, 94% purity)

Table S7. ^{1}H and ^{13}C NMR data for **perAc-DHPA-3′-GlcA** (**6b**)
(700.13 MHz for ^{1}H, 176.05 MHz for ^{13}C, $CDCl_3$, 20 °C)

Atom	δ_C	m.	δ_H	n_H	m.	*J* [Hz]	diagnostic HMBC
**1**	101.44	d	4.998	1	d	7.6	5
**2**	71.05	d	5.278	1	dd	9.3, 7.6	
**2-CO**	169.82	s	-	0	-	-	2, Ac
**Ac**	20.72	q	2.123	3	s	-	
**3**	71.30	d	5.357	1	dd	9.3, 9.3	Ac
**3-CO**	169.96	s	-	0	-	-	3, Ac
**Ac**	20.60	q	2.062	3	s	-	
**4**	68.95	d	5.314	1	dd	9.4, 9.3	Ac
**4-CO**	169.41	s	-	0	-	-	4, Ac
**Ac**	20.49	q	2.054	3	s	-	
**5**	72.46	d	4.172	1	d	9.4	1, OMe
**5-CO**	166.69	s	-	0	-	-	5, OMe
**OMe**	52.96	q	3.772	3	s	-	

**1′**	132.79	s	-	0	-	-	1′′, 2′′
**2′**	117.89	d	6.803	1	d	1.9	
**3′**	143.77	s	-	0	-	-	1, 2′, 5′
**4′**	145.85	s	-	0	-	-	OH, 2′, 5′, 6′
**5′**	116.49	d	6.871	1	d	8.2	OH
**6′**	125.13	d	6.852	1	dd	8.2, 1.9	
**4′-OH**	-	-	6.030	1	s	-	
**1′′**	30.12	t	2.853	2	t	7.7	2′, 6′
**2′′**	35.78	t	2.579	2	t	7.7	OMe
**2′′-CO**	173.26	s	-	0	-	-	2′′, OMe
**OMe**	51.66	q	3.668	3	s	-	

Table S8. ^{1}H and ^{13}C NMR data for **perAc-DHPP-4′-GlcA** (**6b′**)
(700.13 MHz for ^{1}H, 176.05 MHz for ^{13}C, $CDCl_3$, 20 °C)

Atom	δ_C	m.	δ_H	n_H	m.	*J* [Hz]	diagnostic HMBC
**1**	101.64	d	4.970	1	d	7.6	5
**2**	71.05	d	5.269	1	dd	9.3, 7.6	
**2-CO**	169.77	s	-	0	-	-	2, Ac
**Ac**	20.70	q	2.111	3	s	-	
**3**	71.32	d	5.345	1	dd	9.3, 9.3	Ac
**3-CO**	169.97	s	-	0	-	-	3, Ac
**Ac**	20.60	q	2.058	3	s	-	
**4**	68.95	d	5.305	1	dd	9.4, 9.3	Ac
**4-CO**	169.40	s	-	0	-	-	4, Ac
**Ac**	20.49	q	2.049	3	s	-	
**5**	72.47	d	4.145	1	d	9.4	1, OMe
**5-CO**	166.69	s	-	0	-	-	5, OMe
**OMe**	53.17	q	3.766	3	s	-	
**1′**	138.20	s	-	0	-	-	1′′, 2′′
**2′**	116.33	d	6.794	1	d	2.1	OH
**3′**	147.72	s	-	0	-	-	OH, 2′, 5′,
**4′**	142.47	s	-	0	-	-	1, 2′, 5′, 6′
**5′**	118.09	d	6.869	1	d	8.2	
**6′**	120.11	d	6.655	1	dd	8.2, 2.1	
**3′-OH**	-	-	6.117	1	s	-	
**1′′**	30.33	t	2.280	2	t	7.8	2′, 6′
**2′′**	35.52	t	2.588	2	t	7.8	OMe
**2′′-CO**	173.22	s	-	0	-	-	2′′, OMe
**OMe**	51.67	q	3.668	3	s	-	

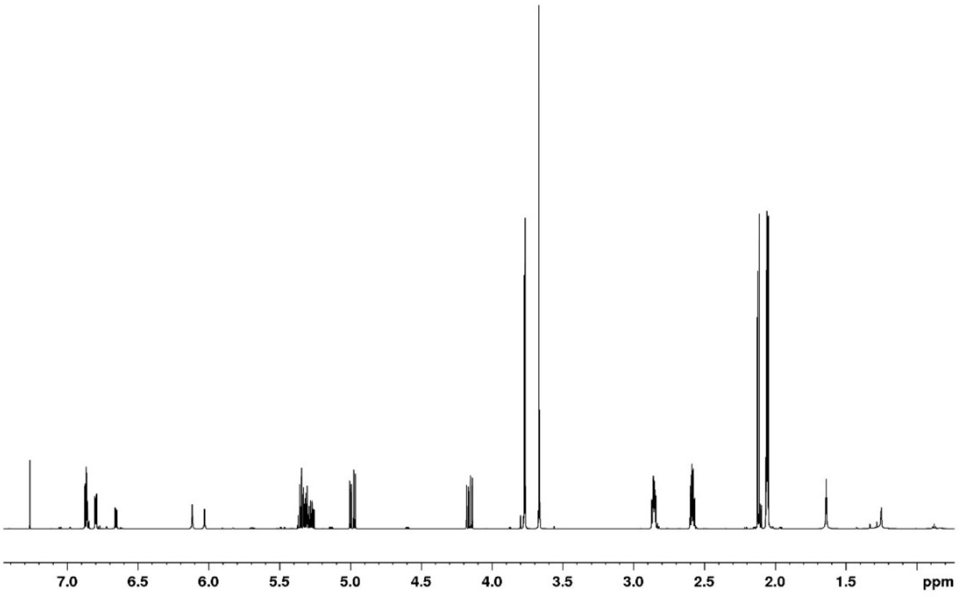

Figure S27. ^{1}H NMR spectrum of **perAc-DHPP-GlcA** (**6b** + **6b'**)
(700.13 MHz, $CDCl_3$, 20 °C)

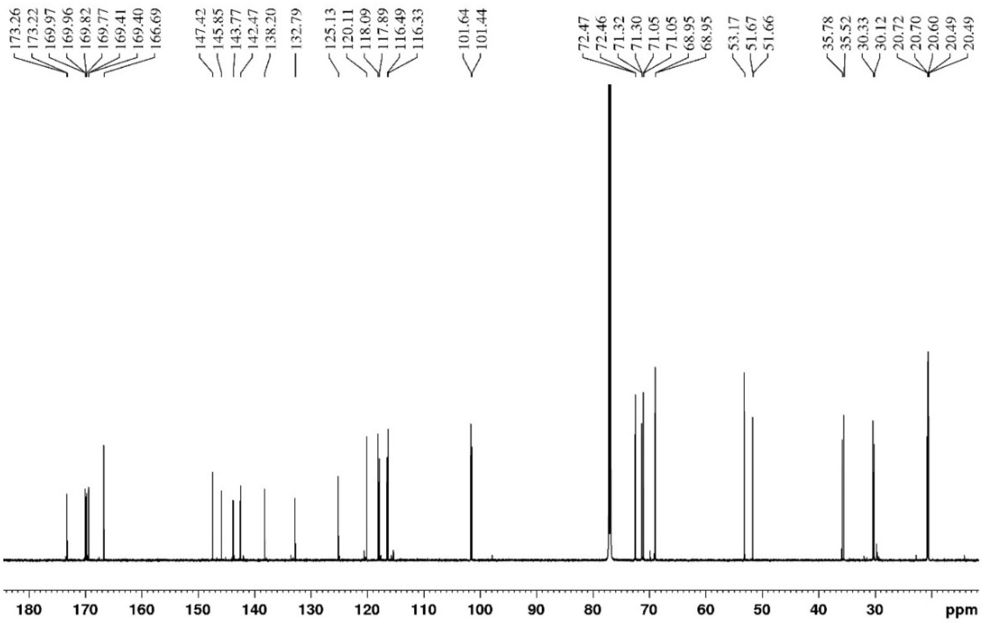

Figure S28. ^{13}C NMR spectrum of **perAc-DHPP-GlcA** (**6b** + **6b'**)
(176.05 MHz, $CDCl_3$, 20 °C)

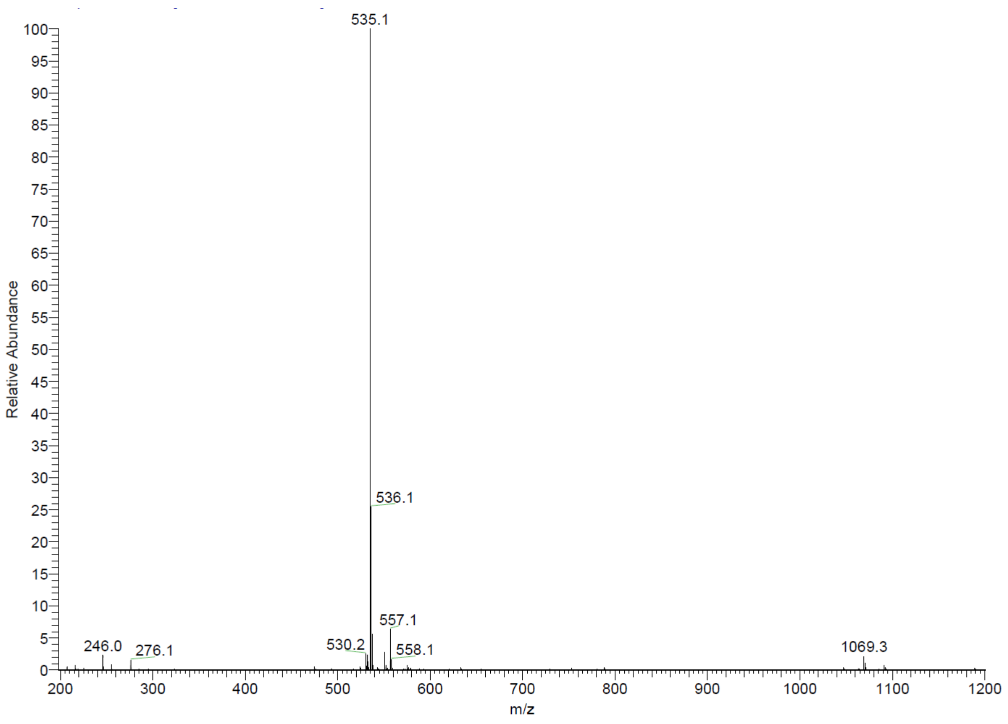

Figure S29. MS (ESI$^+$) spectrum of **perAc-DHPP-GlcA** (**6b** + **6b'**)
([M+ Na]$^+$, *m/z* 535.1)

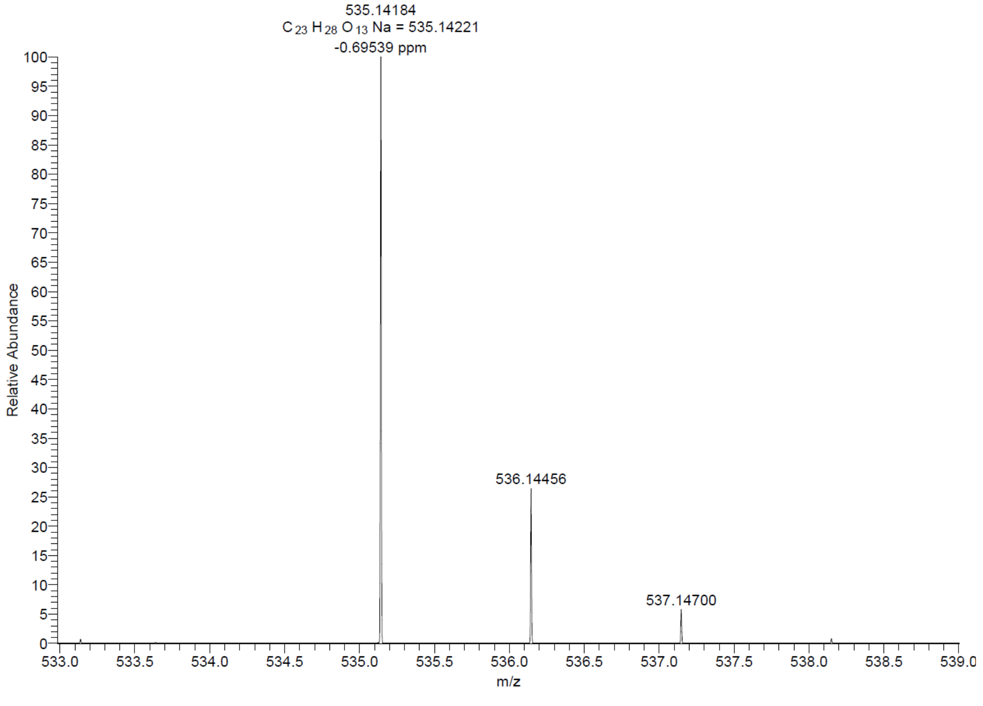

Figure S30. HRMS (ESI$^+$) spectrum of **perAc-DHPP-GlcA** (**6b** + **6b'**)
Calculated (for $C_{23}H_{28}O_{13}Na$) 535.14221, measured 535.14184 (-0.7 ppm)

2. Glucuronides – Free acids

2-Hydroxyphenylacetic acid glucuronide (**2-HPA-GlcA**, **1c**)

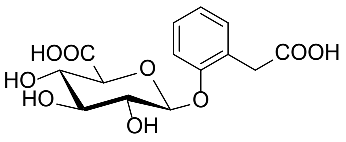

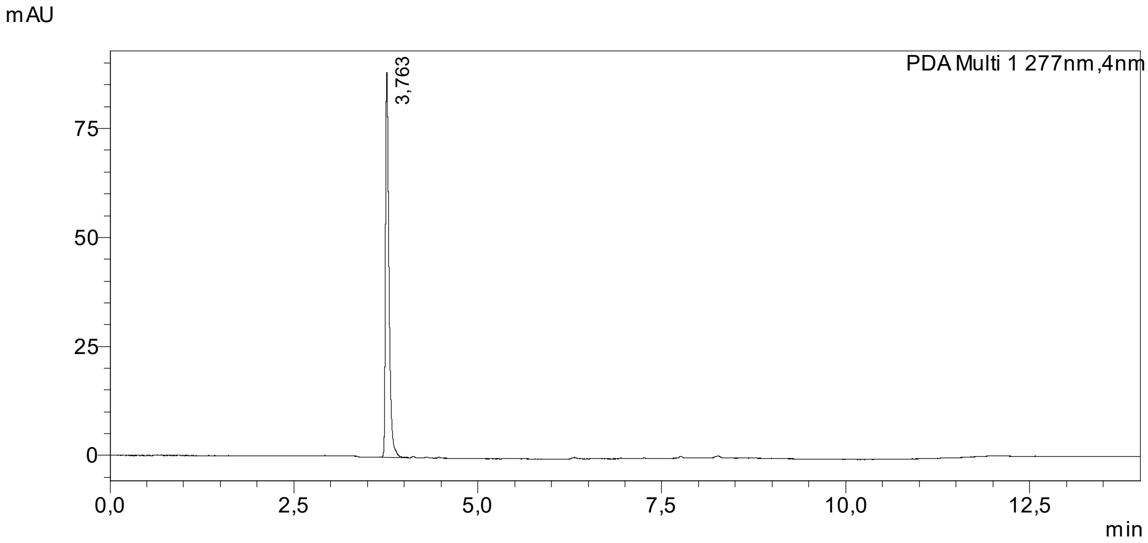

Figure S31. HPLC chromatogram for **2-HPA-GlcA** (**1c**)
(RT= 3.763 min, 98% purity)

Table S9. ^{1}H and ^{13}C NMR data for **2-HPA-GlcA** (**1c**)
(600.23 MHz for ^{1}H, 150.93 MHz for ^{13}C, D_2O, 30 °C)

Atom	δ_C	m.	δ_H	n_H	m.	*J* [Hz]	diagnostic HMBC
**1**	100.83	d	5.160	1	m	-	5
**2**	72.79	d	3.67^H	1	m	-	
**3**	75.40	d	3.67^H	1	m	-	1, 4, 5
**4**	71.36	d	3.70^H	1	m	-	1, 5
**5**	74.78	d	4.153	1	m	-	1, 4
**5-CO**	172.31	s	-	0	-	-	5
**1′**	154.94	s	-	0	-	-	1, 3′, 5′, 1″
**2′**	124.55	s	-	0	-	-	4′, 6′, 1″
**3′**	131.78	d	7.326	1	dd	7.5, 1.7	5′, 1″
**4′**	123.85	d	7.168	1	ddd	7.5, 7.5, 1.0	6′
**5′**	129.48	d	7.385	1	ddd	8.3, 7.5, 1.7	3′
**6′**	115.89	d	7.228	1	dd	8.3, 1.0	4′
**1″**	35.72	t	3.824	2	s	-	3′
**1″-CO**	176.90	s	-	0	-	-	1″

^{H} … HSQC readout

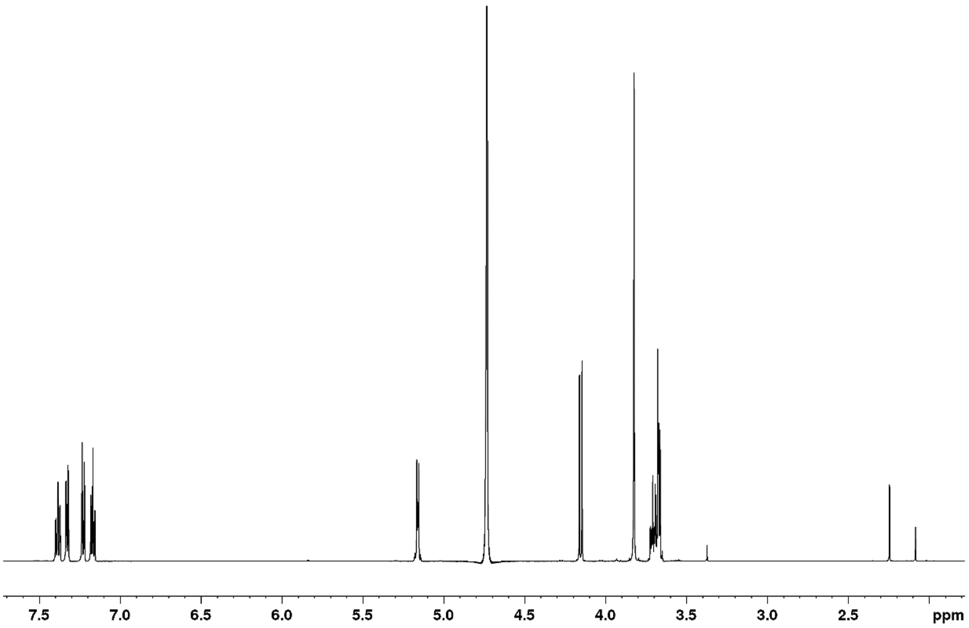

Figure S32. ^{1}H NMR spectrum of **2-HPA-GlcA** (**1c**)
(600.23 MHz, D_2O, 30 °C)

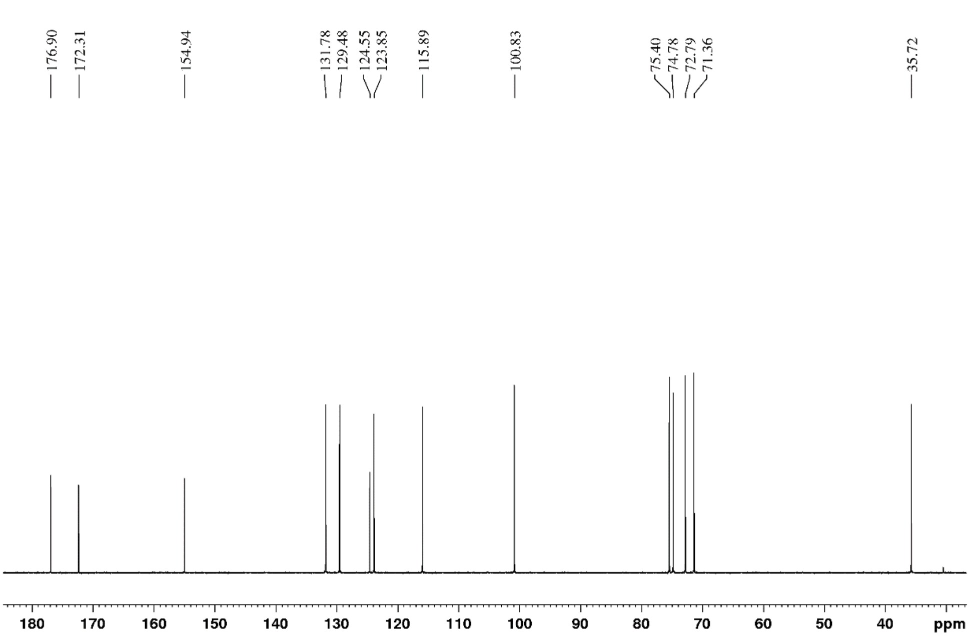

Figure S33. ^{13}C NMR spectrum of **2-HPA-GlcA** (**1c**)
(150.93 MHz, D_2O, 30 °C)

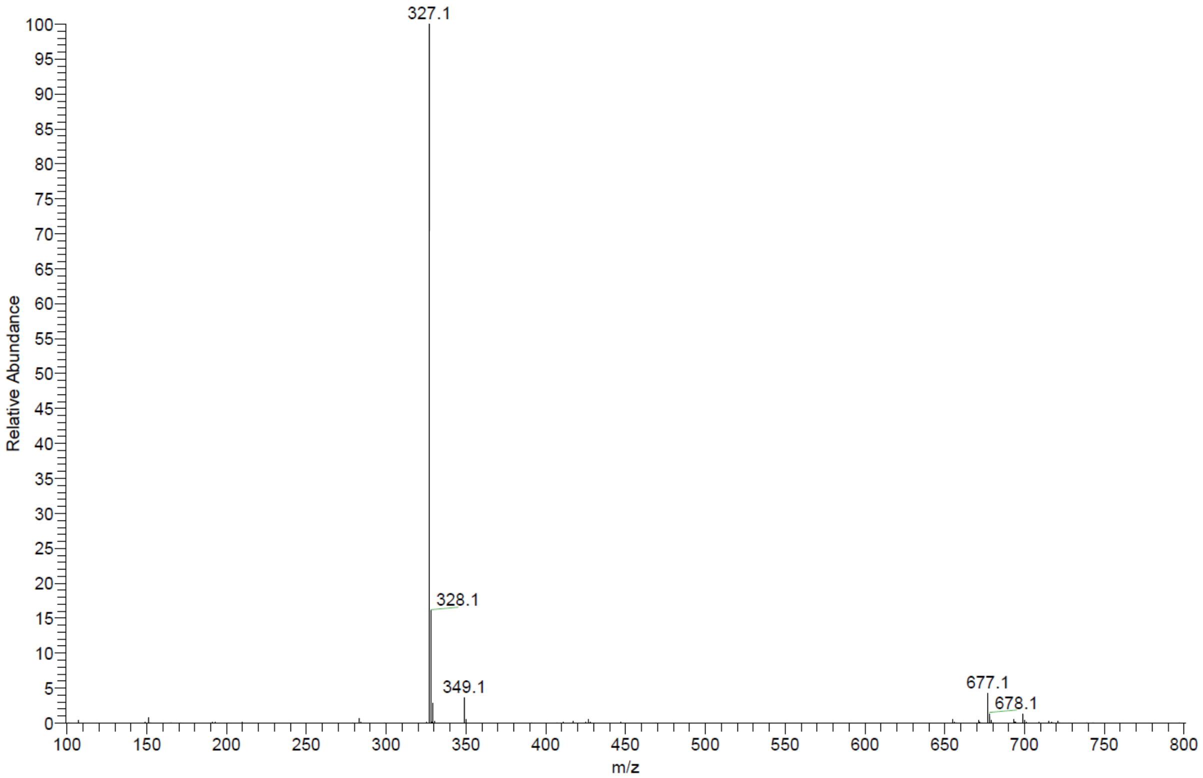

Figure S34. MS (ESI$^-$) spectrum of **2-HPA-GlcA** (**1c**)
([M-H]$^-$, *m/z* 327.1).

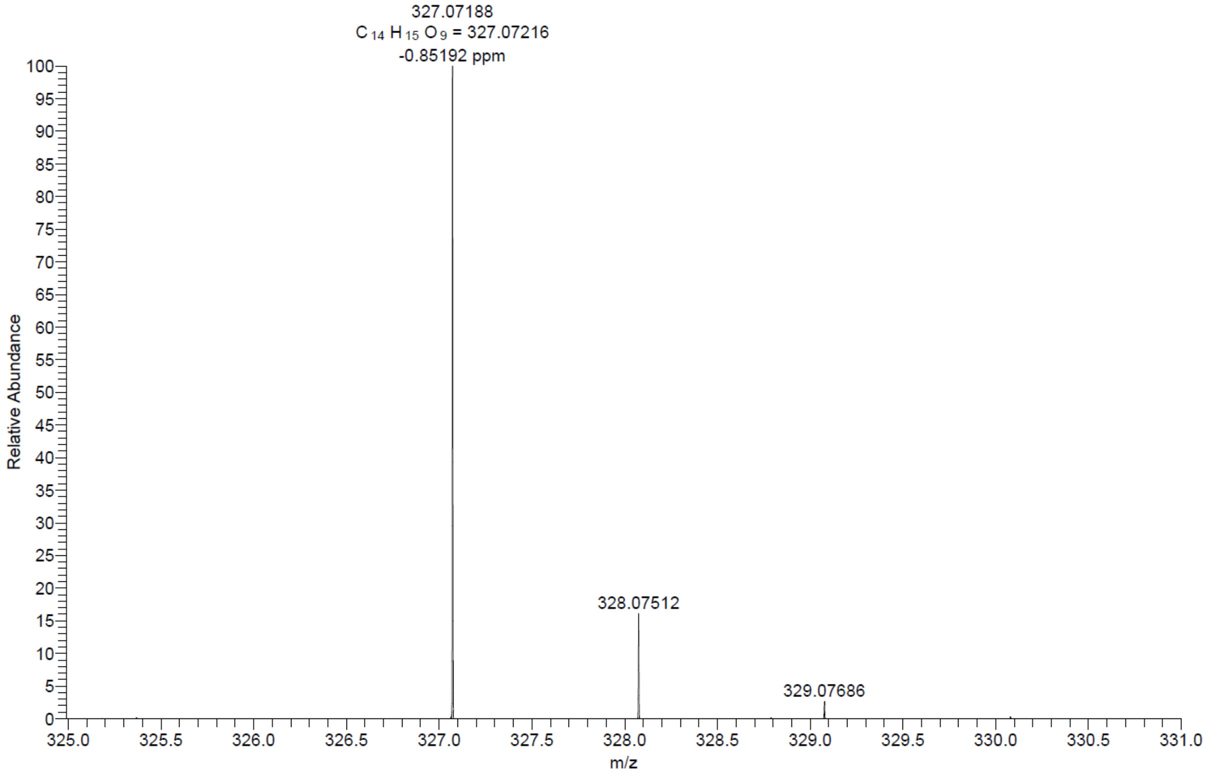

Figure S35. HRMS (ESI$^-$) spectrum of **2-HPA-GlcA** (**1c**)
Calculated (for $C_{14}H_{15}O_9$) 327.07216, measured 327.07188 (-0.85 ppm)

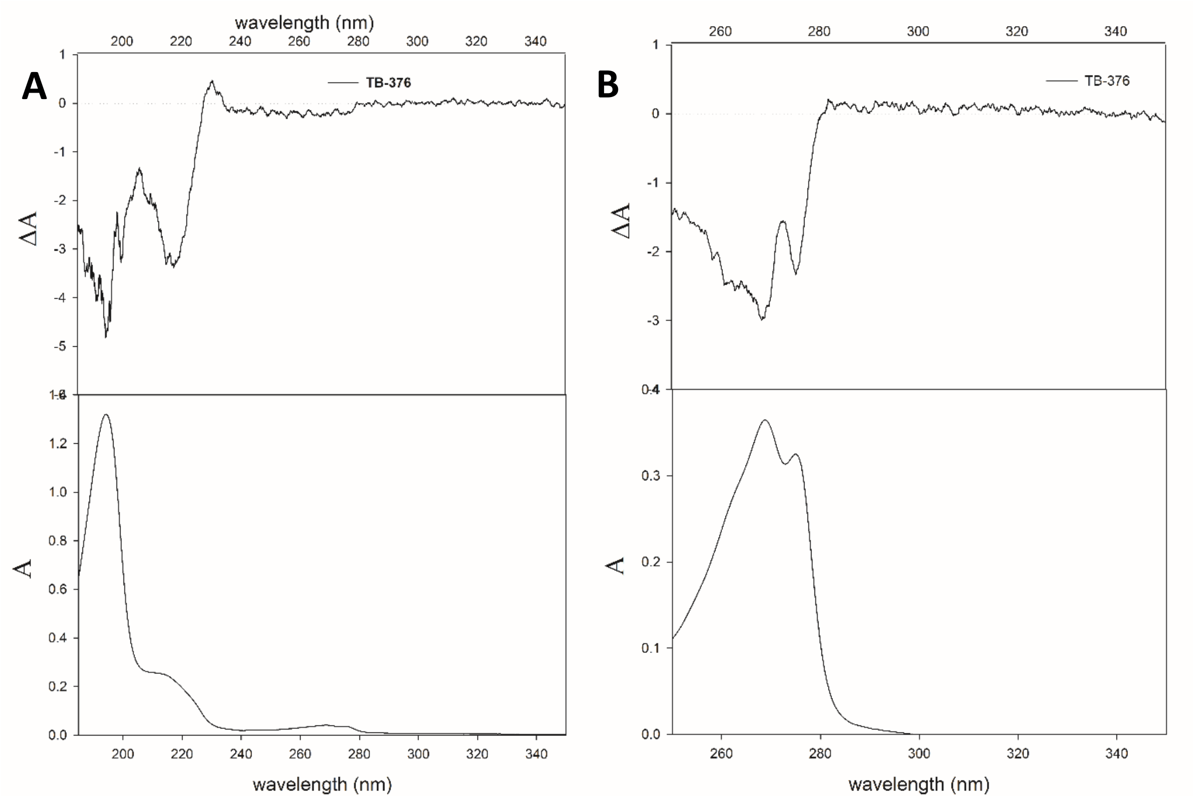

Figure S36. CD spectrum of **2-HPA-GlcA** (**1c**)
in the spectral range 190–350 nm (**A**) and in the near-UV range (250–280 nm, **B**)

3-Hydroxyphenylacetic acid glucuronide (**3-HPA-GlcA**, **2c**)

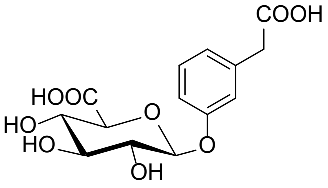

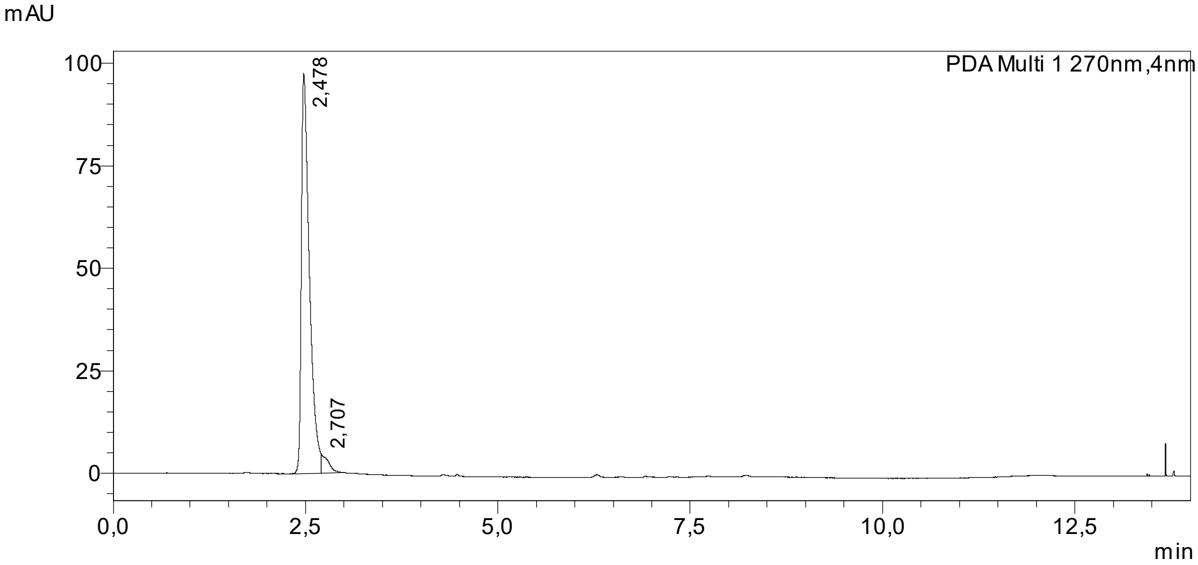

Figure S37. HPLC chromatogram for **3-HPA-GlcA** (**2c**)
(RT= 2.478 min, 96% purity)

Table S10. ^{1}H and ^{13}C NMR data for **3-HPA-GlcA (2c)**
(600.23 MHz for ^{1}H, 150.93 MHz for ^{13}C, D_2O, 30 °C)

Atom	δ_C	m.	δ_H	n_H	m.	*J* [Hz]	diagnostic HMBC
**1**	100.40	d	5.202	1	d	7.4	5
**2**	72.78	d	3.66^{H}	1	m	-	
**3**	75.25	d	3.68^{H}	1	m	-	1, 5
**4**	71.36	d	3.71^{H}	1	m	-	5
**5**	74.71	d	4.193	1	m	-	1
**5-CO**	172.19	s	-	0	-	-	5
**1′**	156.81	s	-	0	-	-	1, 5′
**2′^{x}**	117.99	d	7.07^{H}	1	m	-	1″
**3′**	136.16	s	-	0	-	-	5′, 1″
**4′^{x}**	124.73	d	7.08^{H}	1	m	-	1″
**5′**	130.37	d	7.384	1	m	-	
**6′**	115.77	d	7.09^{H}	1	m	-	5′
**1″**	40.50	t	3.744	2	s	-	
**1″-CO**	176.61	s	-	0	-	-	1″

^{H} … HSQC readout, ^{x} … might be interchanged

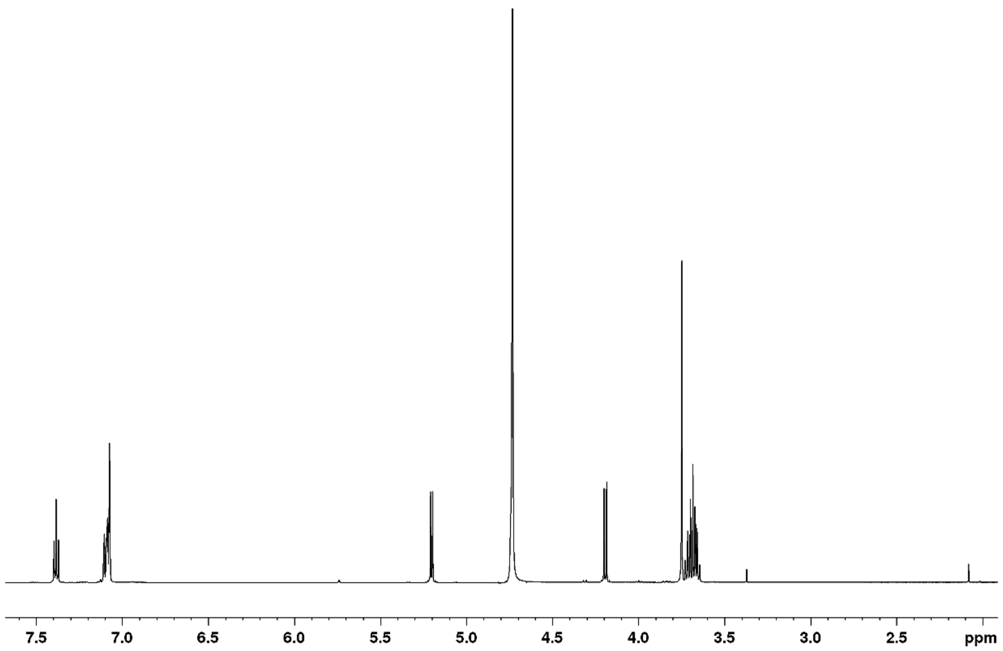

Figure S38. ^{1}H NMR spectrum of **3-HPA-GlcA** (**2c**)
(600.23 MHz, D_2O, 30 °C)

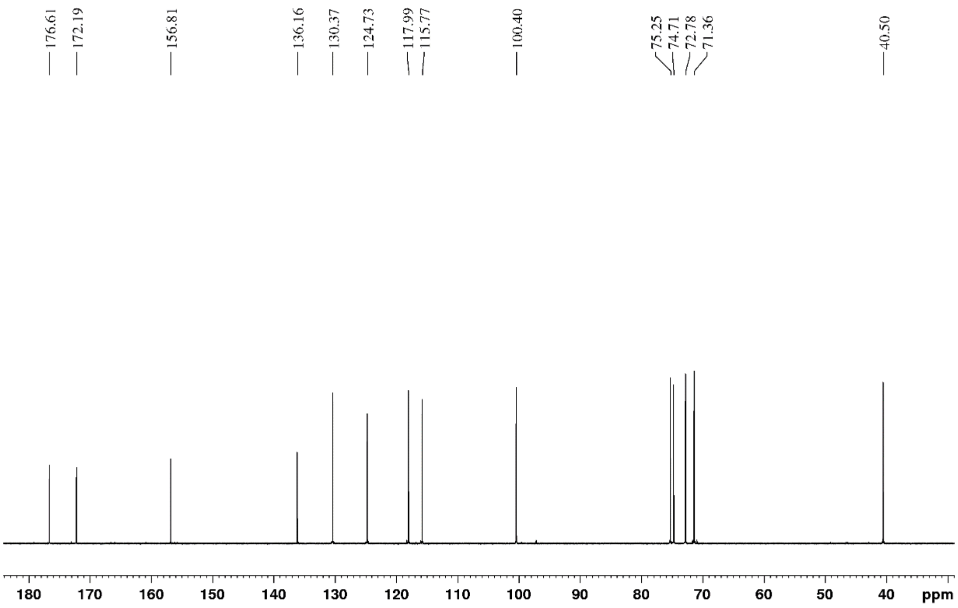

Figure S39. ^{13}C NMR spectrum of **3-HPA-GlcA** (**2c**)
(150.93 MHz, D_2O, 30 °C)

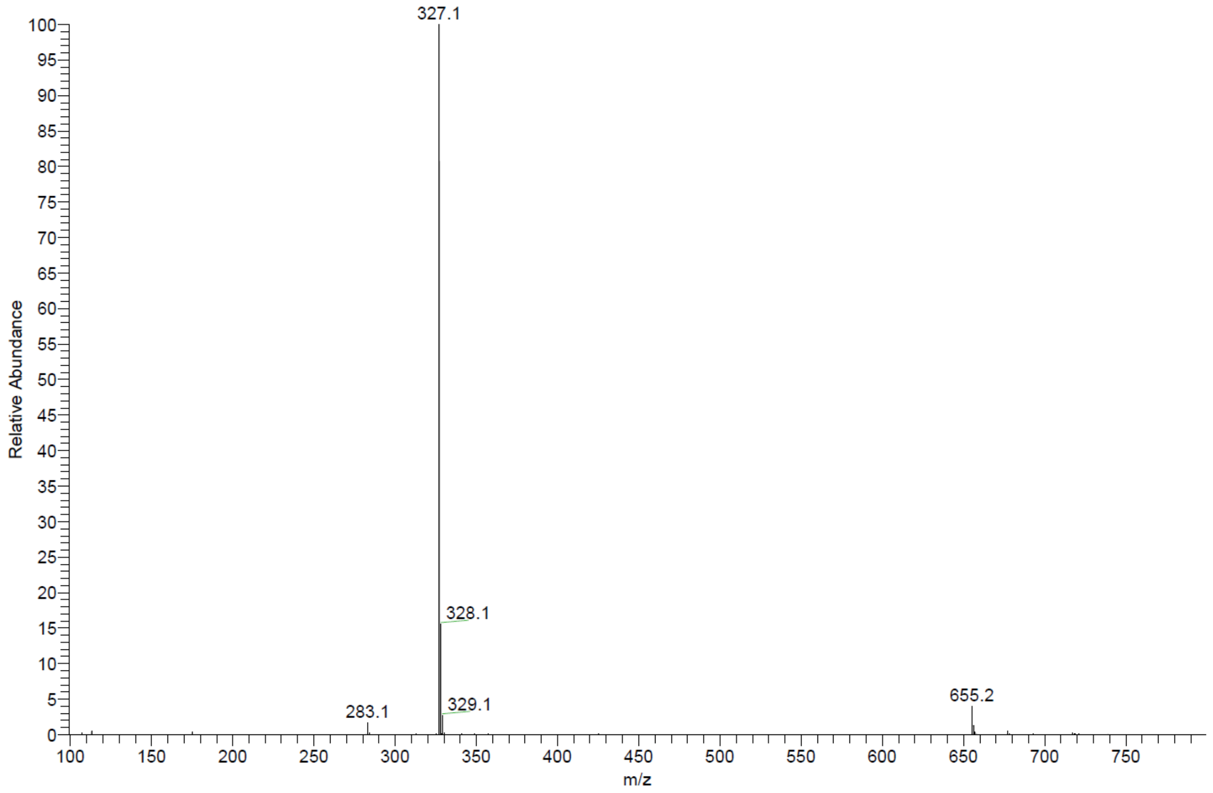

Figure S40. MS (ESI$^-$) spectrum of **3-HPA-GlcA** (**2c**)
([M-H]$^-$, *m/z* 327.1)

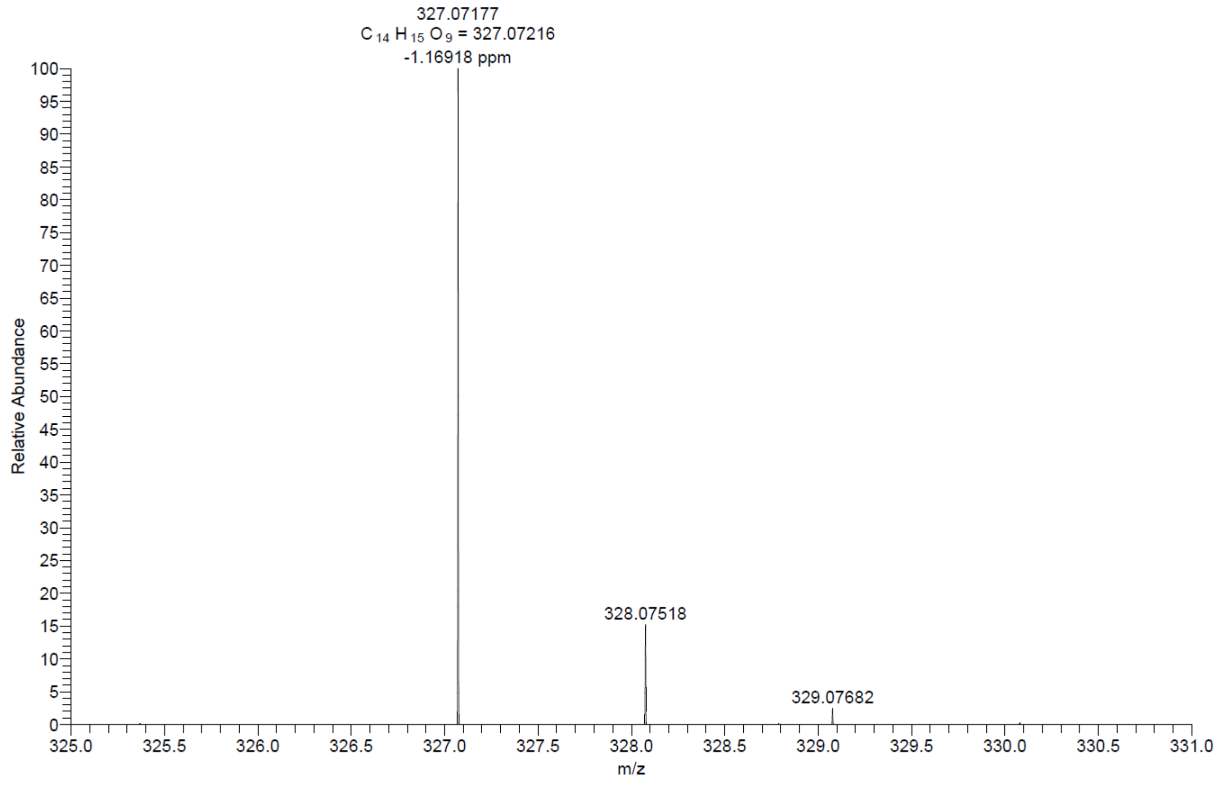

Figure S41. HRMS (ESI$^-$) spectrum of **3-HPA-GlcA** (**2c**)
Calculated (for $C_{14}H_{15}O_9$) 327.07216, measured 327.07177 (-1.17 ppm)

4-Hydroxyphenylacetic acid glucuronide (**4-HPA-GlcA**, **3c**)

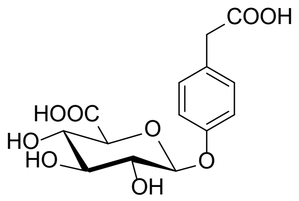

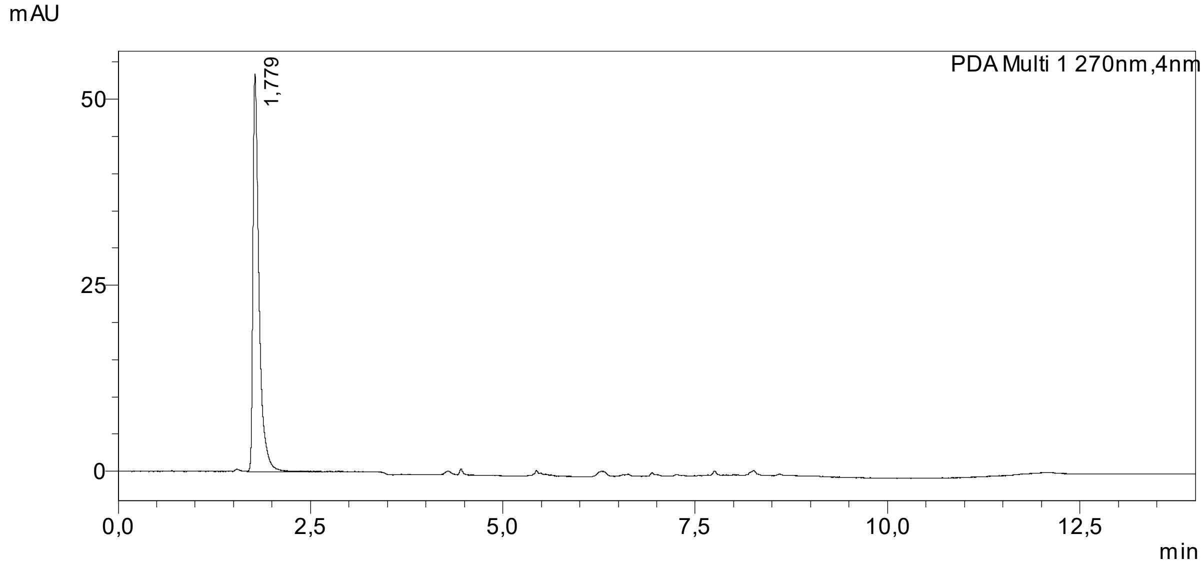

Figure S42. HPLC chromatogram for **4-HPA-GlcA** (**3c**)
(RT= 1.779 min, 93% purity)

Table S11. ^{1}H and ^{13}C NMR data for **4-HPA-GlcA** (**3c**)
(600.23 MHz for ^{1}H, 150.93 MHz for ^{13}C, D_2O, 30 °C)

Atom	δ_C	m.	δ_H	n_H	m.	*J* [Hz]	diagnostic HMBC
**1**	100.48	d	5.180	1	d	7.5	5
**2**	72.78	d	3.65^{H}	1	m	-	
**3**	75.26	d	3.68^{H}	1	m	-	1, 5
**4**	71.38	d	3.70^{H}	1	m	-	5
**5**	74.73	d	4.183	1	m	-	1
**5-CO**	172.22	s	-	0	-	-	5
ipso-	155.84	s	-	0	-	-	1, *o-*, *m-*
ortho-	117.17^{x}	d	7.130	2	m	Σ*J* = 8.8	*o-*, *m-*
meta-	131.03^{x}	d	7.292	2	m	Σ*J* = 8.8	1′, *m-*
para-	129.32	s	-	0	-	-	1′, *o-*
**1′**	39.81	t	3.714	2	s	-	*m-*
**1′-CO**	177.04	s	-	0	-	-	1′

^{x} … 2C; ^{H} … HSQC readout

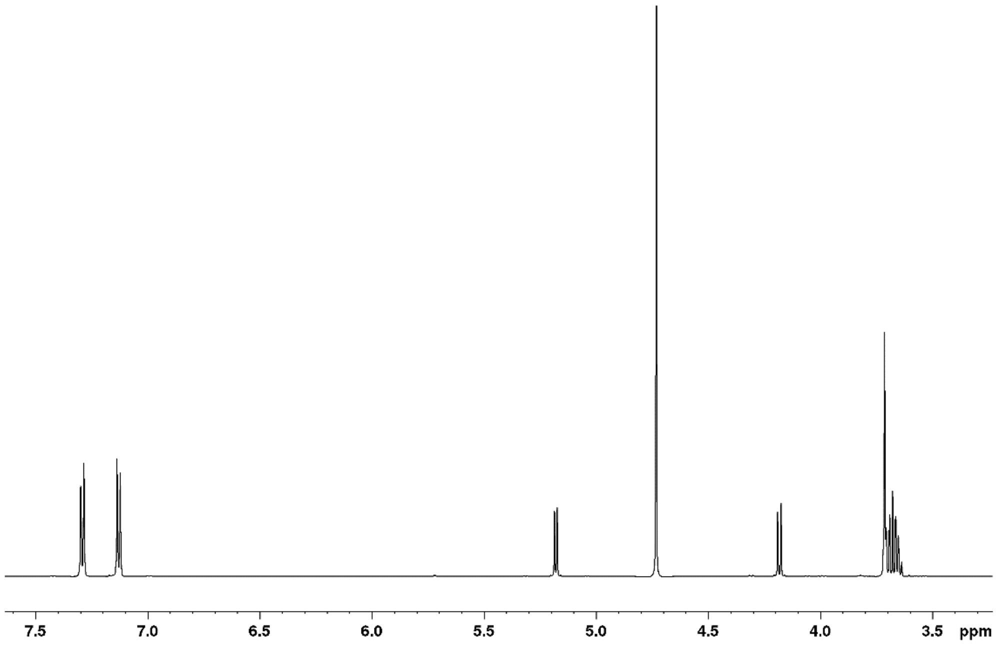

Figure S43. ^{1}H NMR spectrum of **4-HPA-GlcA** (**3c**)
(600.23 MHz, D_2O, 30 °C)

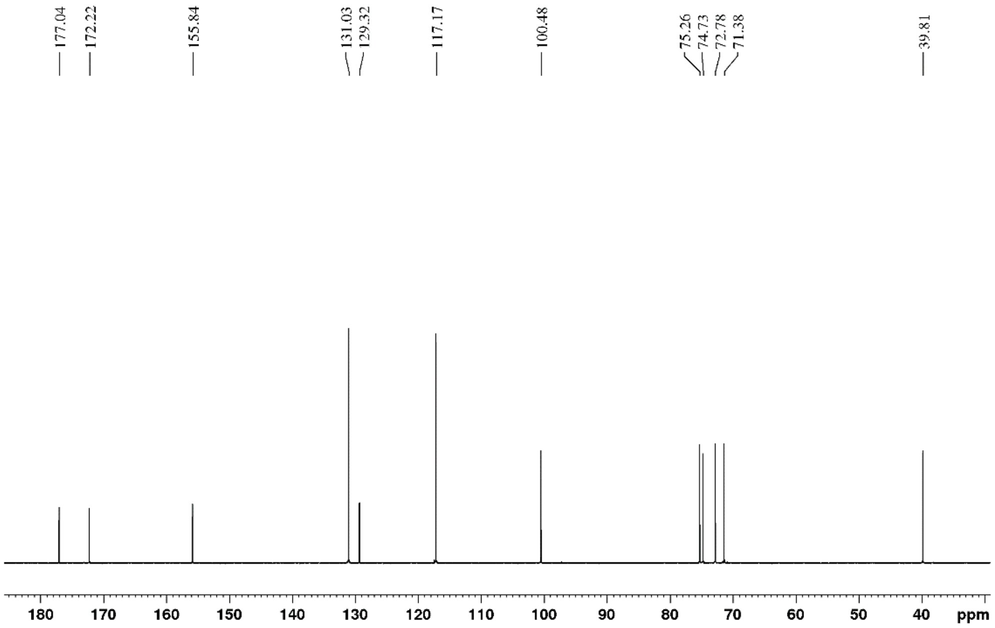

Figure S44. ^{13}C NMR spectrum of **4-HPA-GlcA** (**3c**)
(150.93 MHz, D_2O, 30 °C)

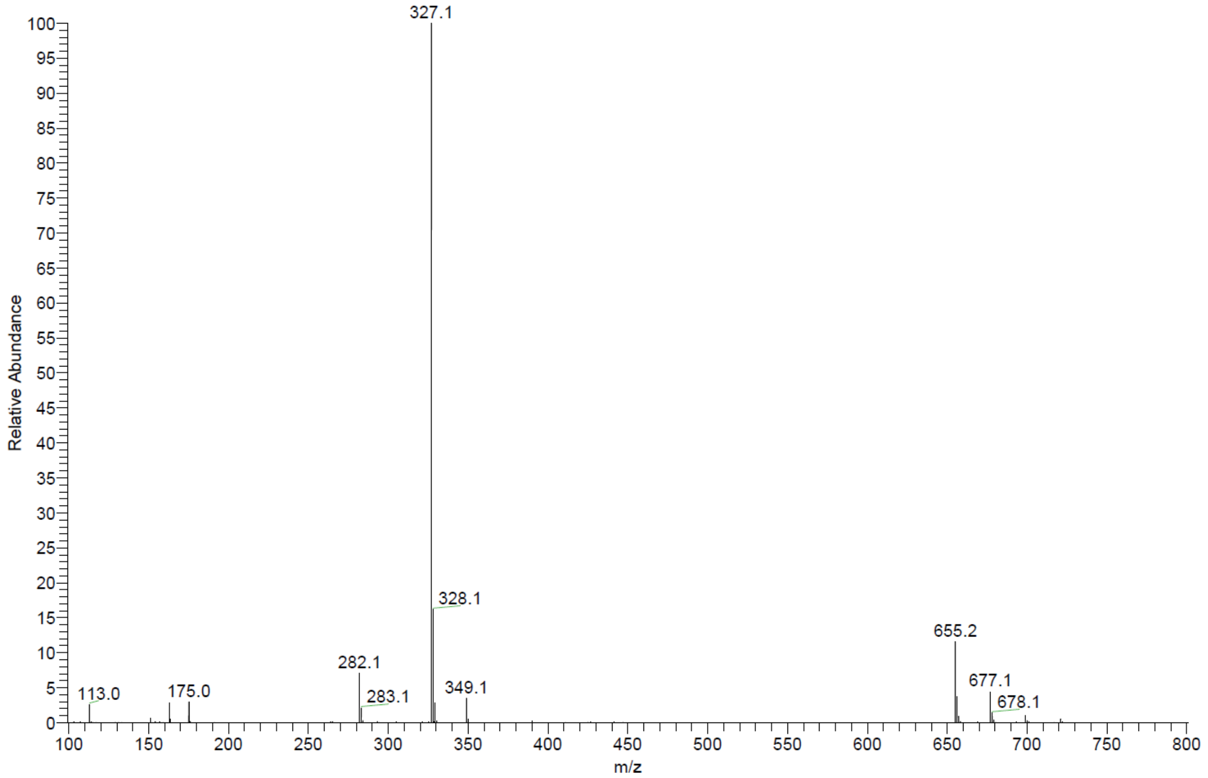

Figure S45. MS (ESI⁻) spectrum of **4-HPA-GlcA** (**3c**)
([M-H]⁻, *m/z* 327.1)

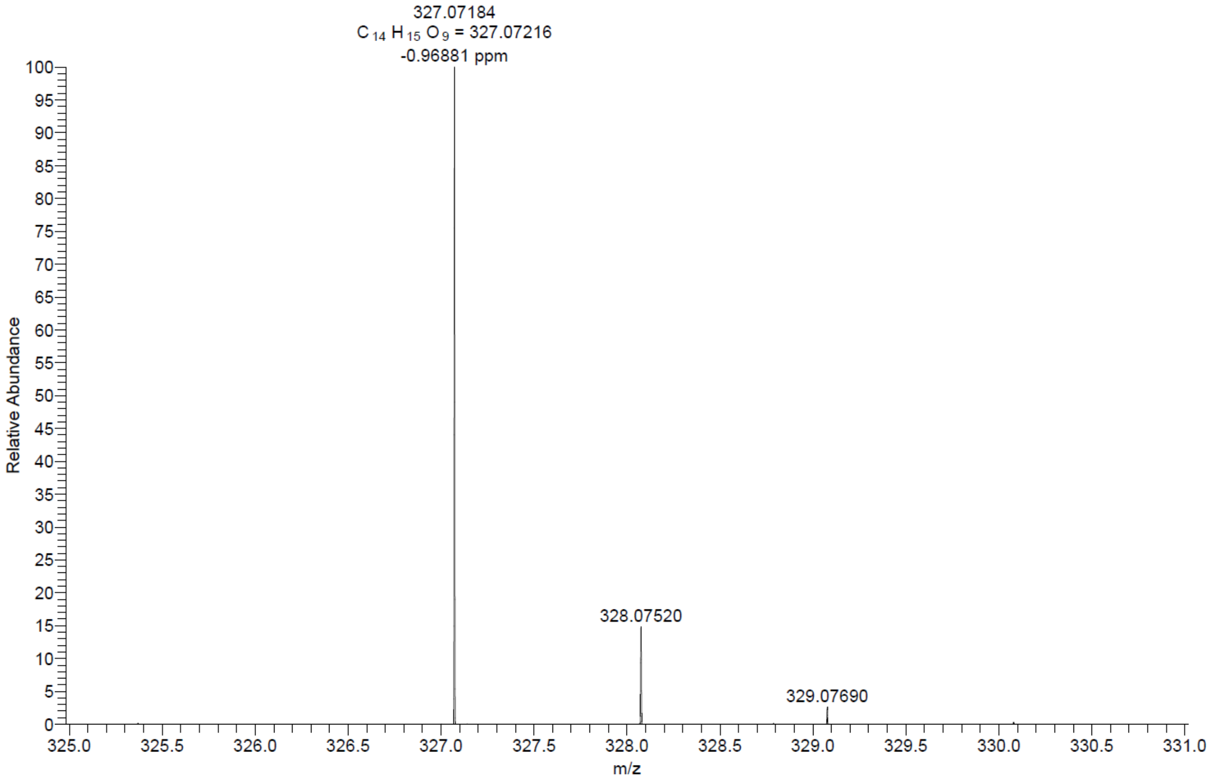

Figure S46. HRMS (ESI⁻) spectrum of **4-HPA-GlcA** (**3c**)
Calculated (for $C_{14}H_{15}O_9$) 327.07216, measured 327.07184 (-0.97 ppm)

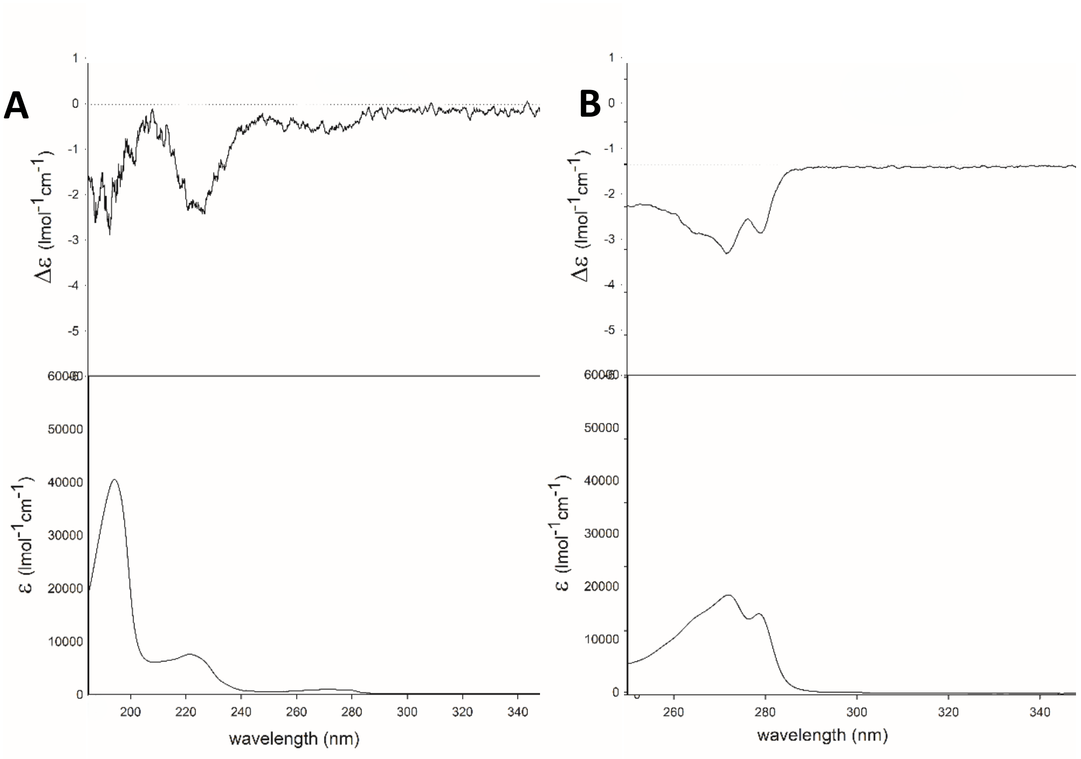

Figure S47. CD spectrum of **4-HPA-GlcA** (**3c**)
in the spectral range 190–350 nm (**A**) and in the near-UV range (250–280 nm, **B**)

4-Hydroxyphenylpropionic acid glucuronide (4-HPP-GlcA, 4c)

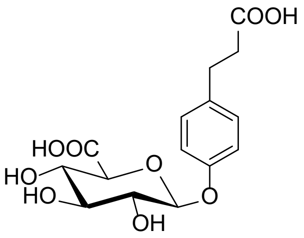

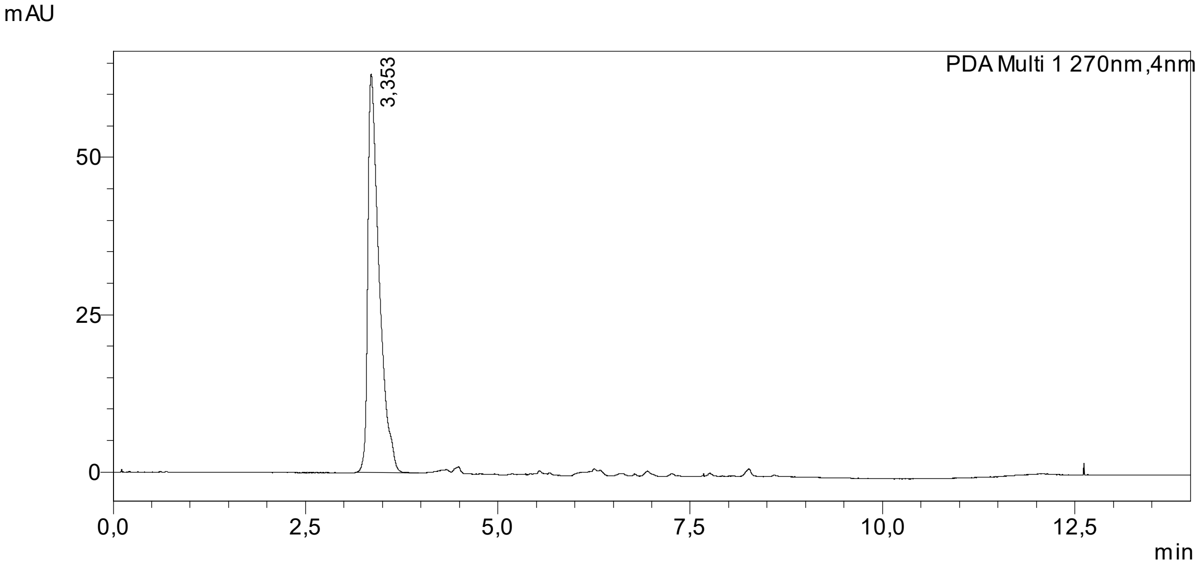

Figure S48. HPLC chromatogram for **4-HPP-GlcA** (**4c**)
(RT= 3.353 min, 97% purity)

Table S12. ^{1}H and ^{13}C NMR data for **4-HPP-GlcA** (**4c**)
(600.23 MHz for ^{1}H, 150.93 MHz for ^{13}C, D_2O, 30 °C)

Atom	δ_C	m.	δ_H	n_H	m.	*J* [Hz]	diagnostic HMBC
**1**	100.66	d	5.131	1	d	7.4	5
**2**	72.79	d	3.64^{H}	1	m	-	
**3**	75.28	d	3.67^{H}	1	m	-	1, 5
**4**	71.39	d	3.70^{H}	1	m	-	5
**5**	74.74	d	4.154	1	m	-	1
**5-CO**	172.25	s	-	0	-	-	5
ipso-	155.15	s	-	0	-	-	1, *o-, m-*
ortho-	117.15^{x}	d	7.083	2	m	Σ*J* = 8.6	*o-, m-*
meta-	129.79^{x}	d	7.247	2	m	Σ*J* = 8.6	1′, *m-*
para-	135.92	s	-	0	-	-	1′, 2′, *o-*
**1′**	29.64	t	2.898	2	t	7.4	*o-*
**2′**	35.59	t	2.681	2	t	7.4	
**2′-CO**	178.06	s	-	0	-	-	1′, 2′

^{x} … 2C; ^{H} … HSQC readout

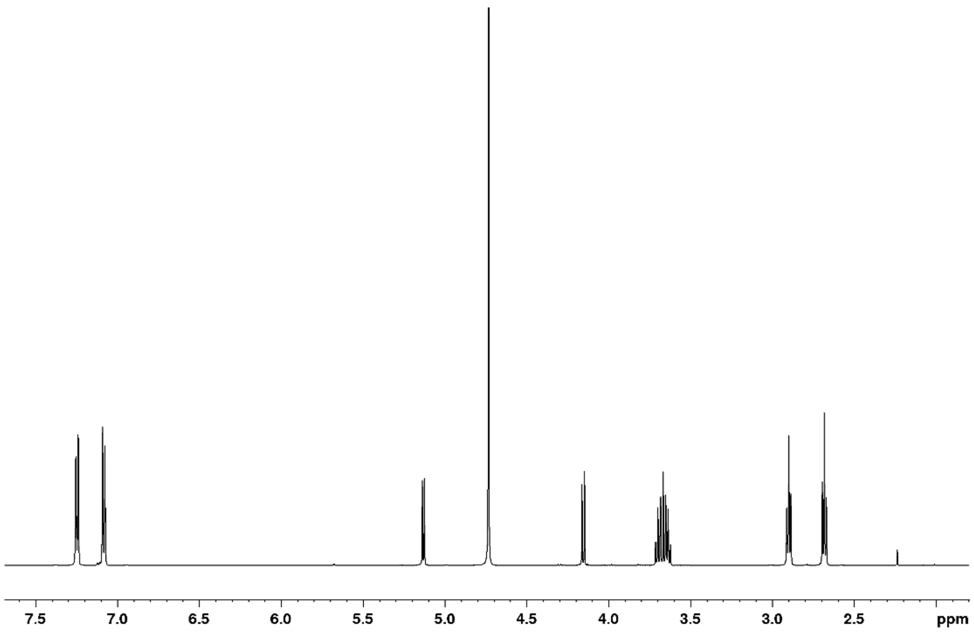

Figure S49. ^{1}H NMR spectrum of **4-HPP-GlcA** (**4c**)
(600.23 MHz, D_2O, 30 °C)

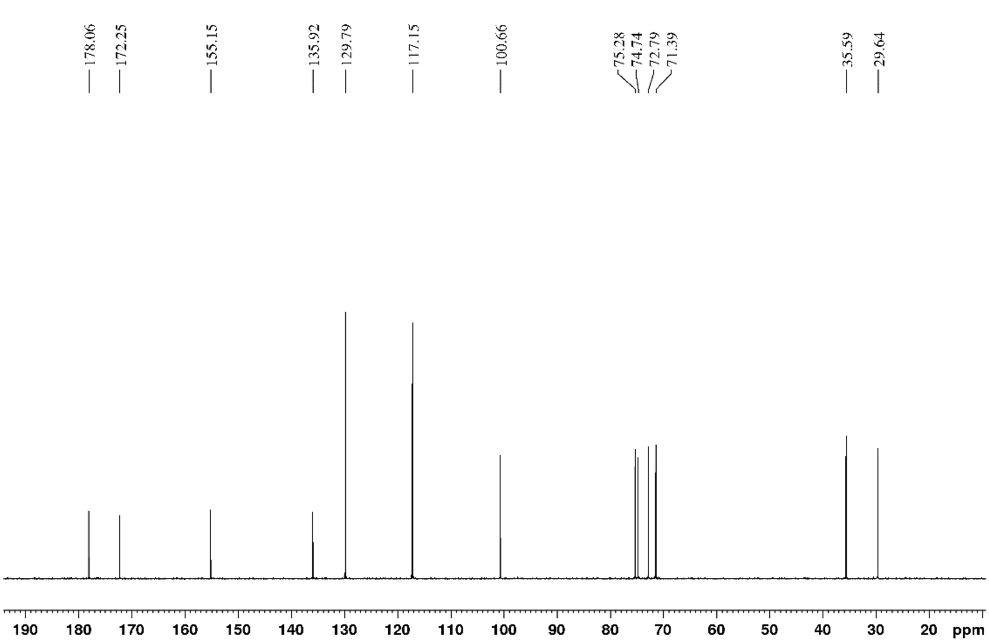

Figure S50. ^{13}C NMR spectrum of **4-HPP-GlcA** (**4c**)
(150.93 MHz, D_2O, 30 °C)

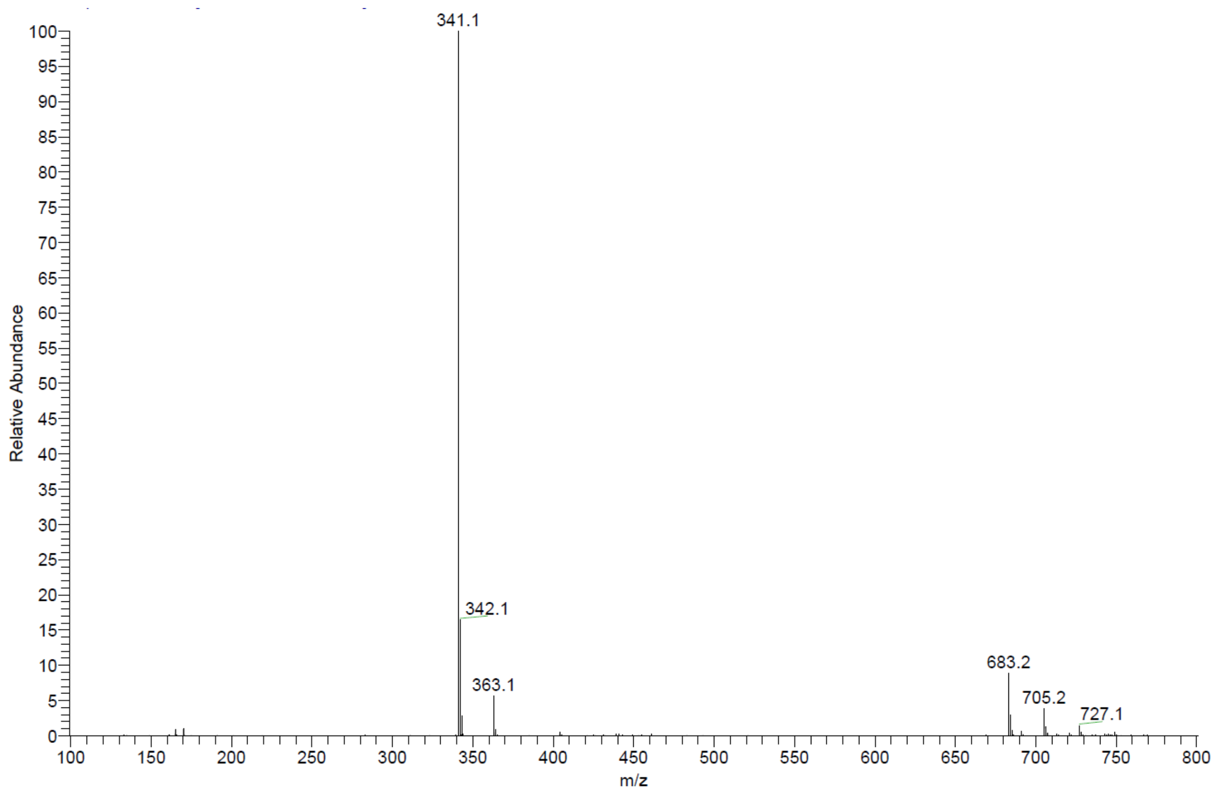

Figure S51. MS (ESI⁻) spectrum of **4-HPP-Glc** (**4c**)
([M-H]$^{-}$, *m/z* 341.1)

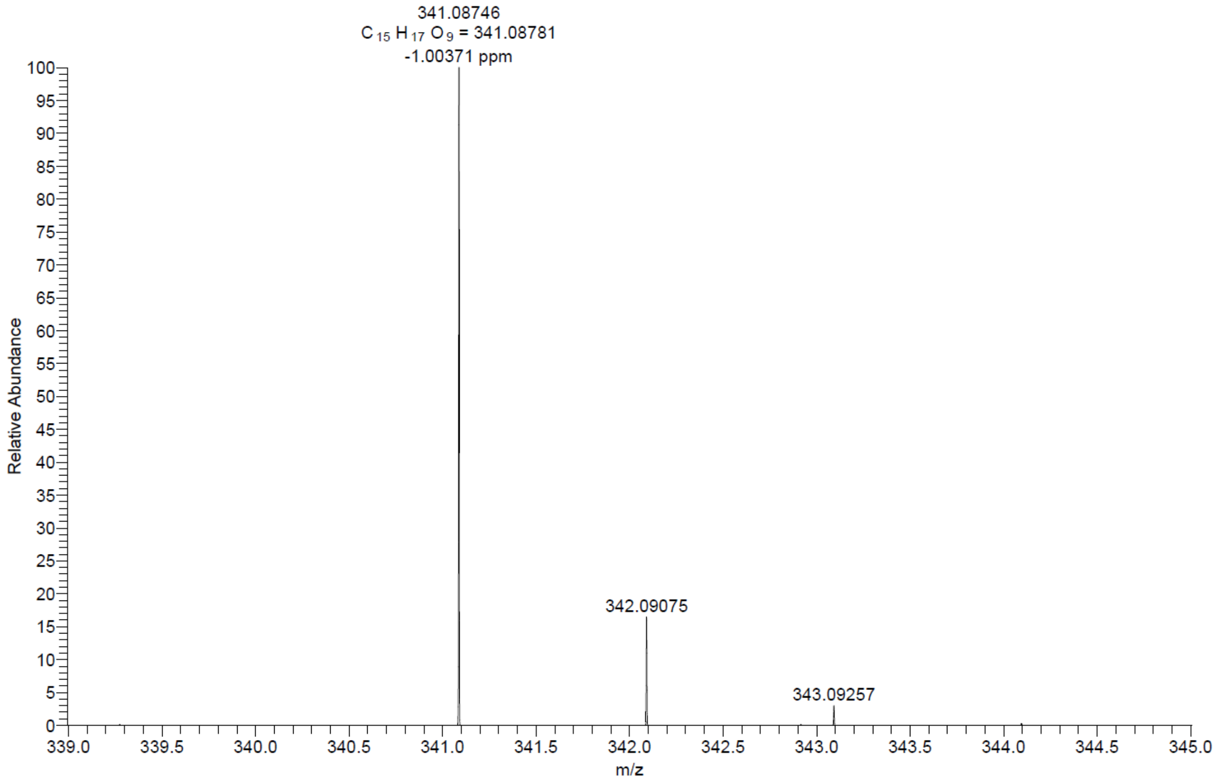

Figure S52. HRMS (ESI^{-}) spectrum of **4-HPP-GlcA** (**4c**)
Calculated (for $C_{15}H_{17}O_9$) 341.08781, measured 341.08746 (-1.00 ppm).

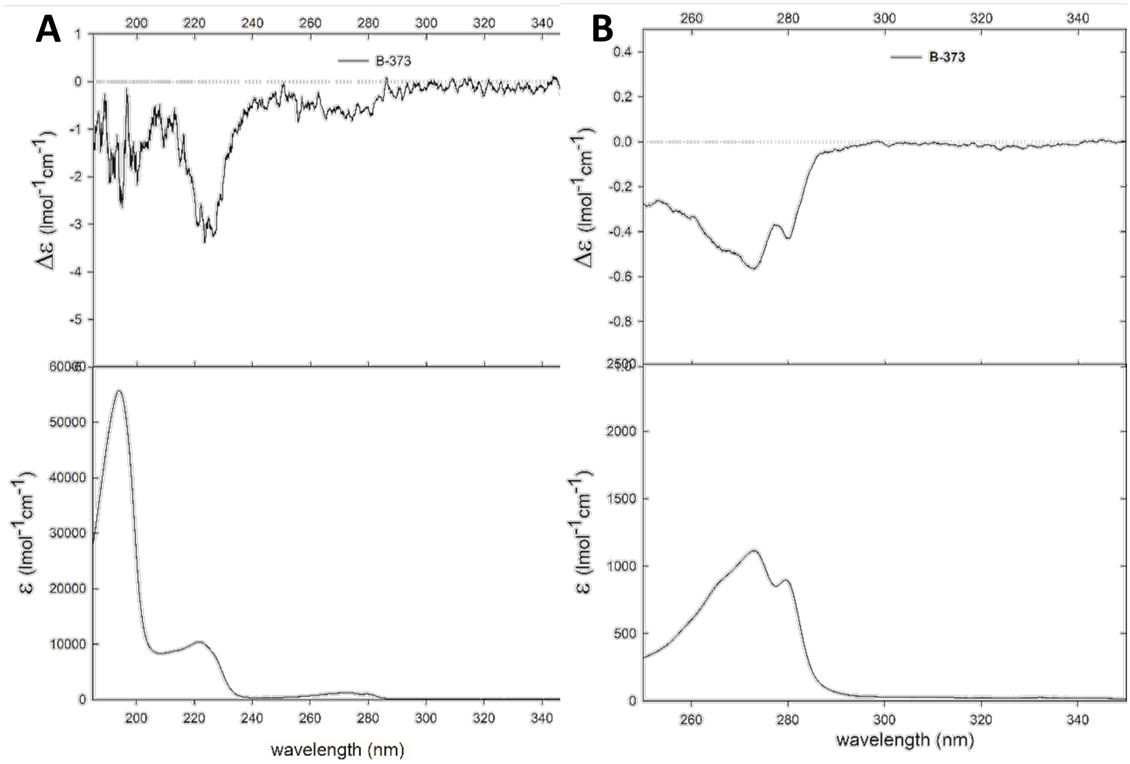

Figure S53. CD spectrum of **4-HPP-GlcA** (**4c**)
in the spectral range 190–350 nm (**A**) and in the near-UV range (250–280 nm, **B**)

3,4-Dihydroxyphenylacetic acid glucuronide (**DHPA-GlcA**, **5c** + **5c′**)

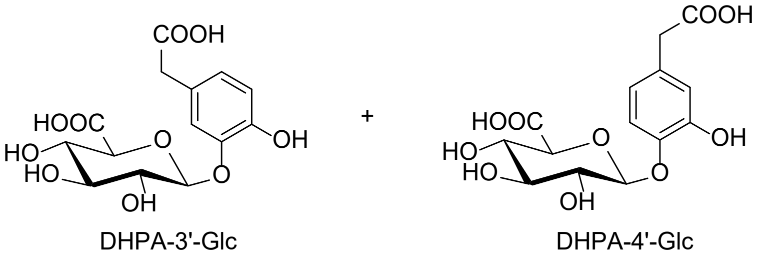

Approximate molar ratio of 3′:4′ isomer = 49:51

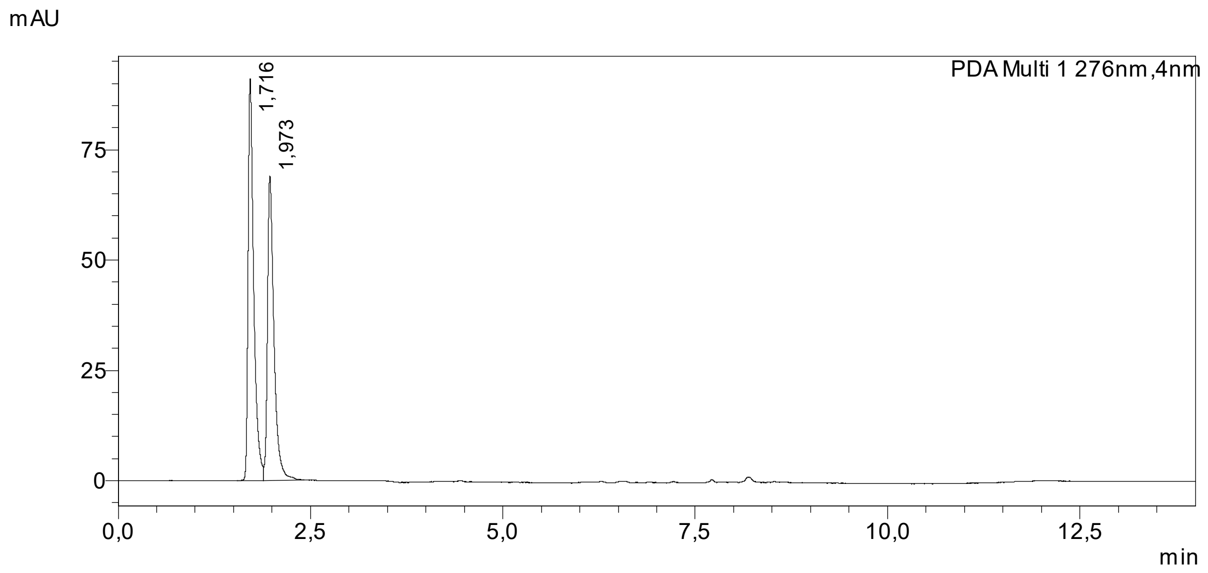

Figure S54. HPLC chromatogram for **DHPA-GlcA** (**5c** + **5c′**)
(RT= 1.973, 1.716 min, 99% purity)

Table S13. ^{1}H and ^{13}C NMR data for **4′-DHPA-GlcA (5c)**
(600.23 MHz for ^{1}H, 150.93 MHz for ^{13}C, D_2O, 30 °C)

Atom	δ_C	m.	δ_H	n_H	m.	*J* [Hz]	diagnostic HMBC
**1**	101.37	d	5.141	1	d	7.5	5
**2**	72.70	d	3.70^{H}	1	m	-	
**3**	75.18	d	3.69^{H}	1	m	-	1, 5
**4**	71.37	d	3.71^{H}	1	m	-	5
**5**	74.81	d	4.158	1	m	-	1
**5-CO**	172.33	s	-	0	-	-	5
**1′**	130.40	s	-	0	-	-	5′, 1″
**2′**	117.80	d	6.921	1	d	2.2	6′, 1″
**3′**	145.84	s	-	0	-	-	2′, 5′
**4′**	143.83	s	-	0	-	-	1, 2′, 5′, 6′
**5′**	117.33	d	7.154	1	d	8.3	6′
**6′**	122.04	d	6.836	1	dd	8.3, 2.2	2′, 1″
**1″**	39.95	t	3.662	2	s	-	3′, 5′
**1″-CO**	176.86	s	-	0	-	-	1″

^{H} … HSQC readout

Table S14. ^{1}H and ^{13}C NMR data for **3′-DHPA-GlcA** (**5c′**)
(600.23 MHz for ^{1}H, 150.93 MHz for ^{13}C, D_2O, 30 °C)

Atom	δ_C	m.	δ_H	n_H	m.	*J* [Hz]	diagnostic HMBC
**1**	101.32	d	5.154	1	d	7.5	5
**2**	72.70	d	3.70^{H}	1	m	-	
**3**	75.18	d	3.69^{H}	1	m	-	1, 5

**4**	71.35	d	3.71[H]	1	m	-	5
**5**	74.81	d	4.158	1	m	-	1
**5-CO**	172.32	s	-	0	-	-	5
**1′**	126.97	s	-	0	-	-	5′, 1″
**2′**	118.23	d	7.097	1	d	1.6	1″
**3′**	144.54	s	-	0	-	-	1, 2′, 5′
**4′**	144.98	s	-	0	-	-	2′, 5′, 6′
**5′**	116.94	d	6.972	1	d	8.2	
**6′**	125.40	d	6.955	1	dd	8.2, 1.6	2′, 1″
**1″**	39.91	t	3.662	2	s	-	2′, 6′
**1″-CO**	176.99	s	-	0	-	-	1″

[H] … HSQC readout

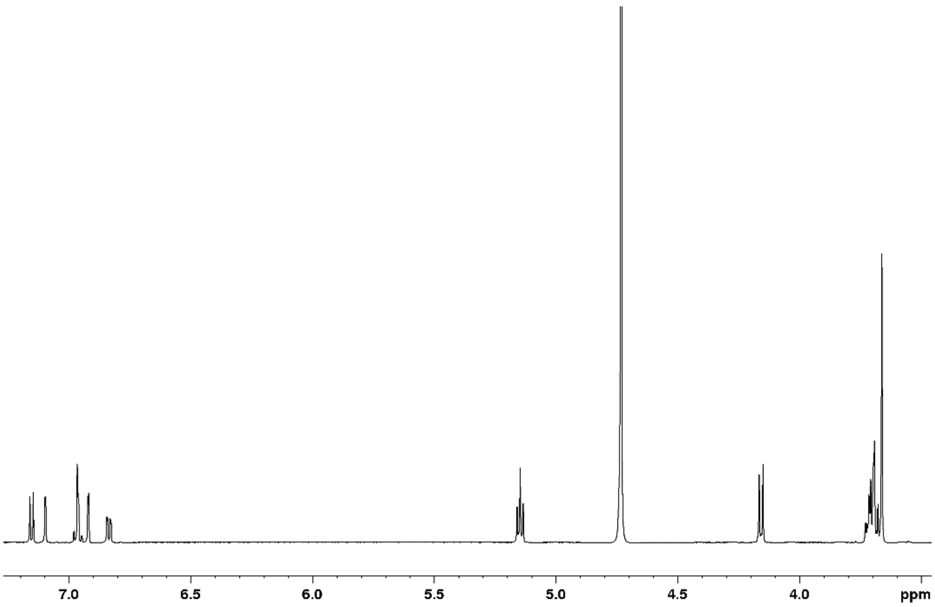

Figure S55. ^{1}H NMR spectrum of **DHPA-GlcA** (**5c** + **5c′**)
(600.23 MHz, D_2O, 30 °C)

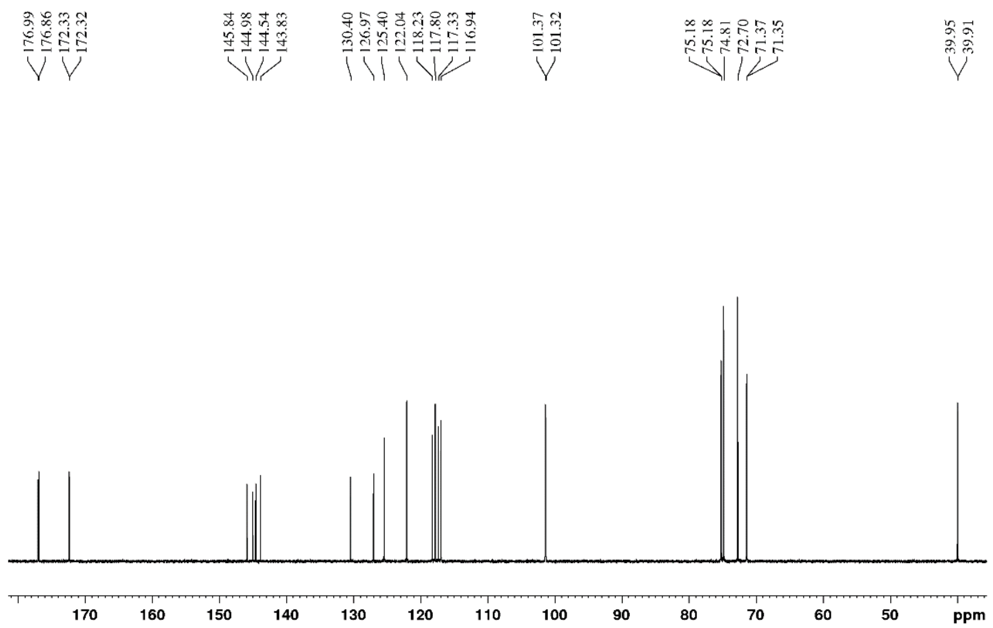

Figure S56. ^{13}C NMR spectrum of **DHPA-GlcA** (**5c** + **5c'**)
(150.93 MHz, D_2O, 30 °C)

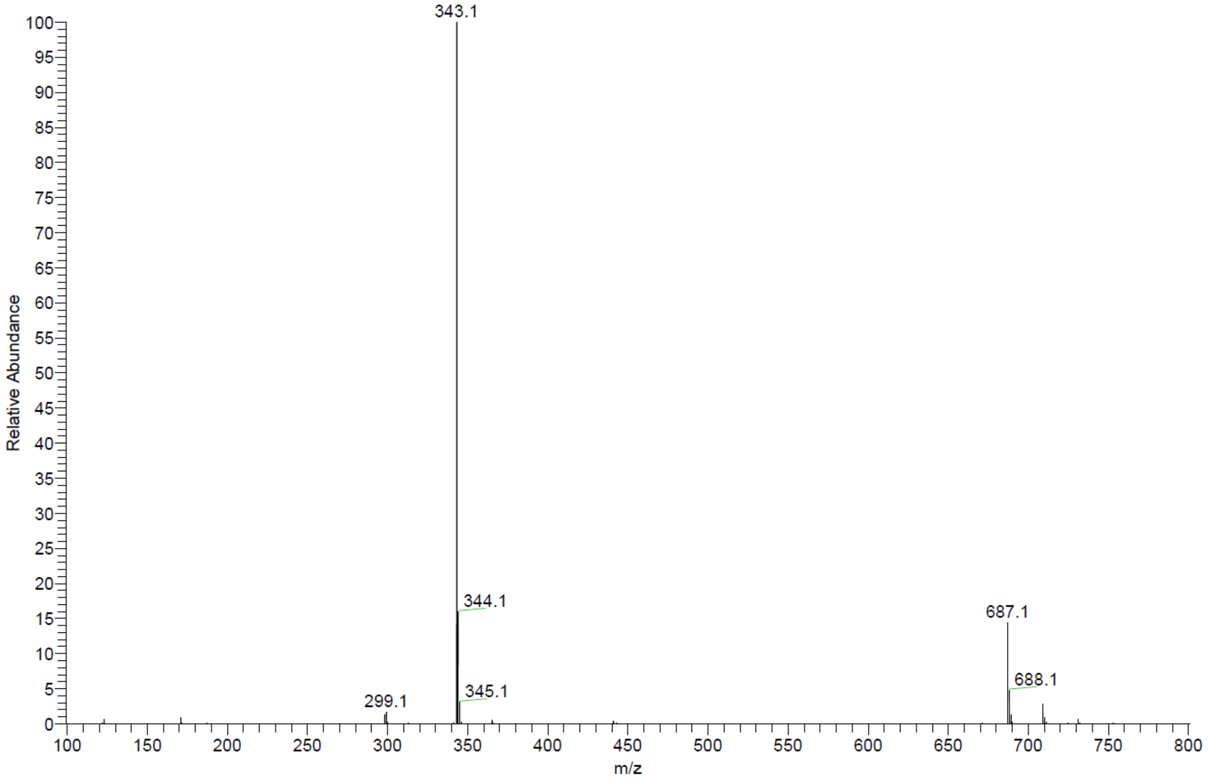

Figure S57. MS (ESI$^-$) spectrum of **DHPA-GlcA** (**5c** + **5c'**)
([M-H]$^-$, *m/z* 343.1)

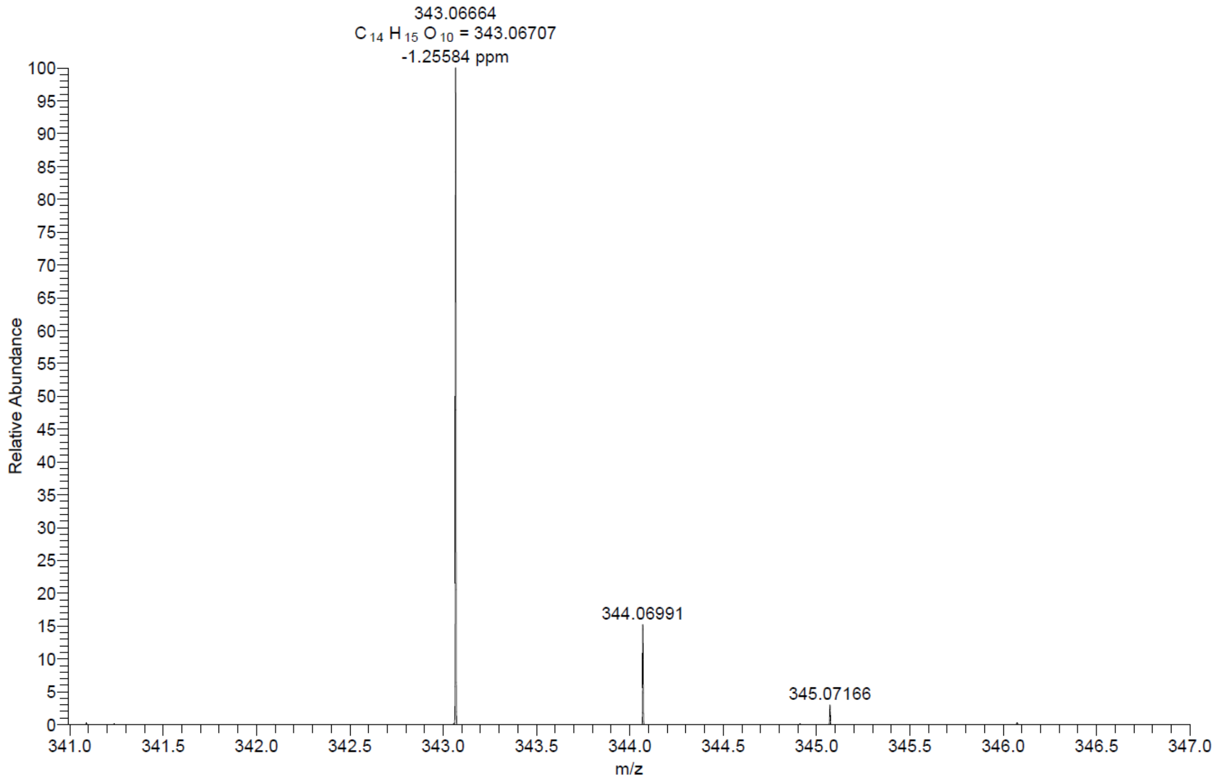

Figure S58. HRMS (ESI⁻) spectrum of **DHPA-GlcA** (**5c** + **5c′**)
Calculated (for $C_{14}H_{15}O_{10}$) 343.06707, measured 343.06664 (-1.26 ppm).

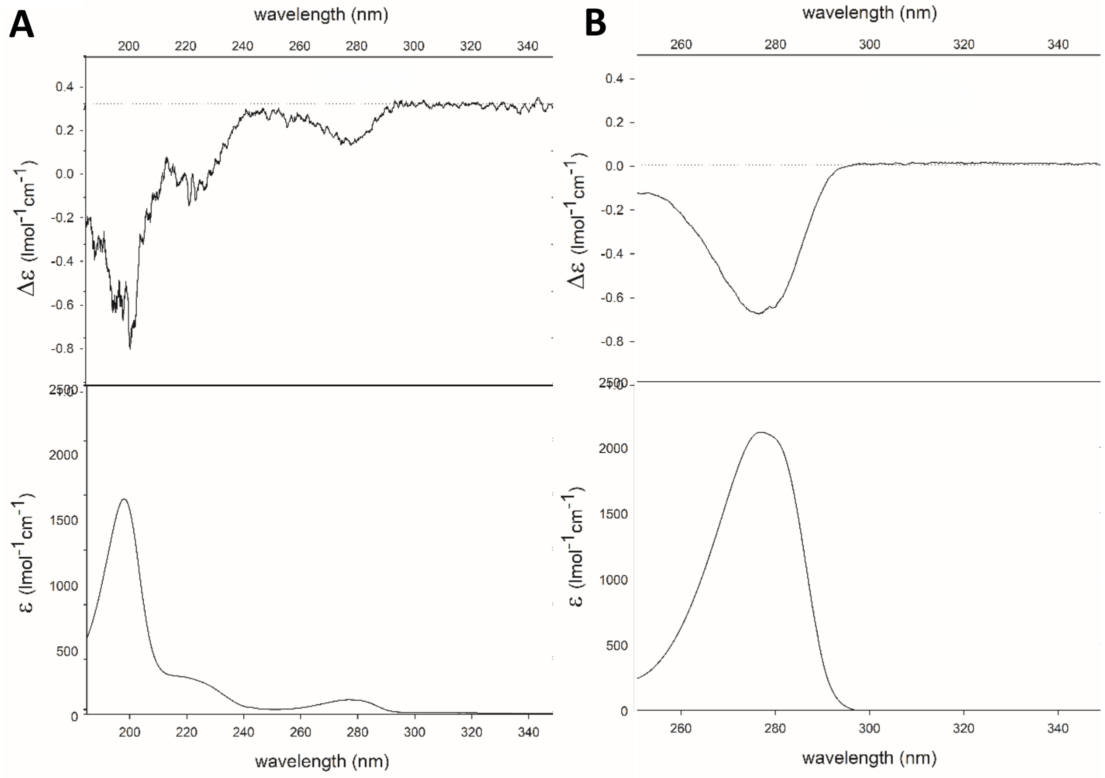

Figure S59. CD spectrum of **DHPA-GlcA** (**5c** + **5c′**)
in the spectral range 190–350 nm (**A**) and in the near-UV range (250–280 nm, **B**)

3-(3,4-Dihydroxyphenyl)propionic acid glucuronide (**DHPP-GlcA**, **6c** + **6c′**)

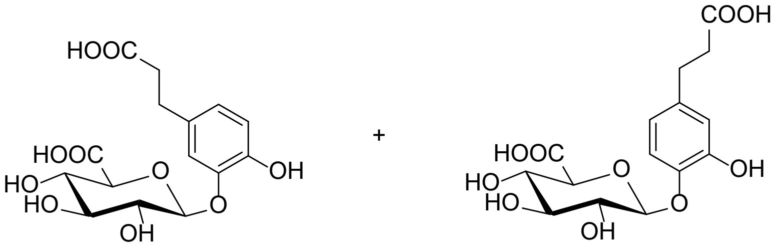

DHPP-3'-Glc **DHPP-4'-Glc**

Approximate molar ratio of 3′:4′ isomer = 42:58

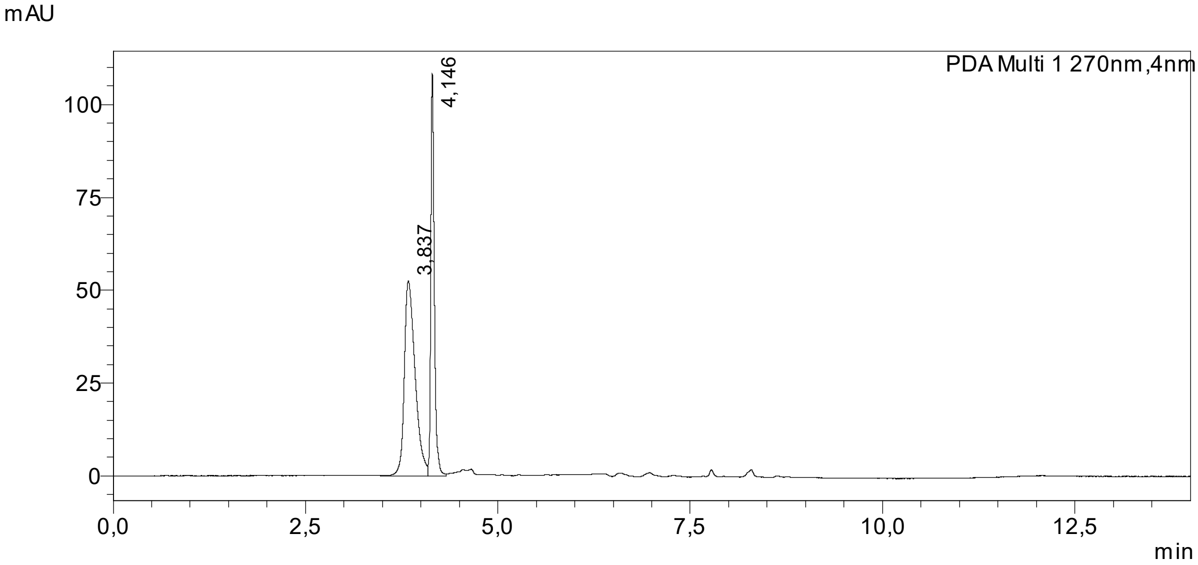

Figure S60. HPLC chromatogram for **DHPP-GlcA** (**6c** + **6c′**)
(RT= 3.837 and 4.146 min, 96% purity)

Table S15. ^{1}H and ^{13}C NMR data for **DHPP-4′-GlcA** (**6c**)
(600.23 MHz for ^{1}H, 150.93 MHz for ^{13}C, D_2O, 30 °C)

Atom	δ_C	m.	δ_H	n_H	m.	*J* [Hz]	diagnostic HMBC
**1**	101.57	d	5.097	1	m	-	5
**2**	72.72	d	3.69^{H}	1	m	-	
**3**	75.20	d	3.68^{H}	1	m	-	1, 5
**4**	71.39	d	3.71^{H}	1	m	-	5
**5**	74.84	d	4.134	1	m	-	1
**5-CO**	172.40	s	-	0	-	-	5
**1′**	137.23	s	-	0	-	-	5′, 1″, 2″
**2′**	116.64	d	6.884	1	d	2.2	1″, 6′
**3′**	145.79	s	-	0	-	-	2′, 5′, 6′
**4′**	143.06	s	-	0	-	-	1, 2′, 5′, 6′
**5′**	117.48	d	7.118	1	d	8.3	6′

**6′**	120.75	d	6.804	1	dd	8.3, 2.1	2′, 1′′
**1″**	29.78	t	2.863	2	dd	Σ*J* = 14.8	2′, 6′
**2″**	35.48	t	2.684	2	dd	Σ*J* = 14.8	
**2″-CO**	178.03	s	-	0	-	-	1′′, 2′′

^{H} … HSQC readout

Table S16. ^{1}H and ^{13}C NMR data for **DHPP-3′-GlcA** (**6c′**)
(600.23 MHz for ^{1}H, 150.93 MHz for ^{13}C, D_2O, 30 °C)

Atom	δ_C	m.	δ_H	n_H	m.	*J* [Hz]	diagnostic HMBC
**1**	101.36	d	5.134	1	m	-	5
**2**	72.72	d	3.69^{H}	1	m	-	
**3**	75.20	d	3.68^{H}	1	m	-	1, 5
**4**	71.38	d	3.71^{H}	1	m	-	5
**5**	74.84	d	4.156	1	d	9.3	1
**5-CO**	172.39	s	-	0	-	-	5
**1′**	133.73	s	-	0	-	-	1′′, 2′′
**2′**	117.05	d	7.055	1	br s	-	1′′
**3′**	144.47	s	-	0	-	-	1, 2′
**4′**	144.09	s	-	0	-	-	2′
**5′**	116.84	d	6.932	1	m	-	
**6′**	124.09	d	6.932	1	m	-	2′, 1′′
**1″**	29.73	t	2.870	2	dd	Σ*J* = 14.7	2′, 6′
**2″**	35.60	t	2.678	2	dd	Σ*J* = 14.7	
**2″-CO**	178.05	s	-	0	-	-	1′′, 2′′

^{H} … HSQC readout

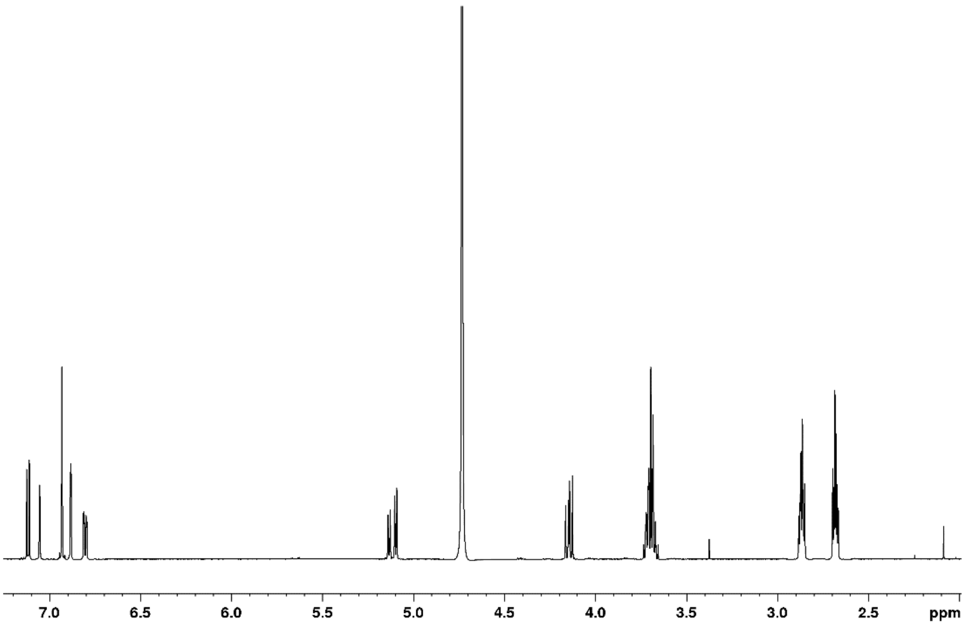

Figure S61. ^{1}H NMR spectrum of **DHPP-GlcA** (**6c** + **6c′**)
(600.23 MHz, D_2O, 30 °C)

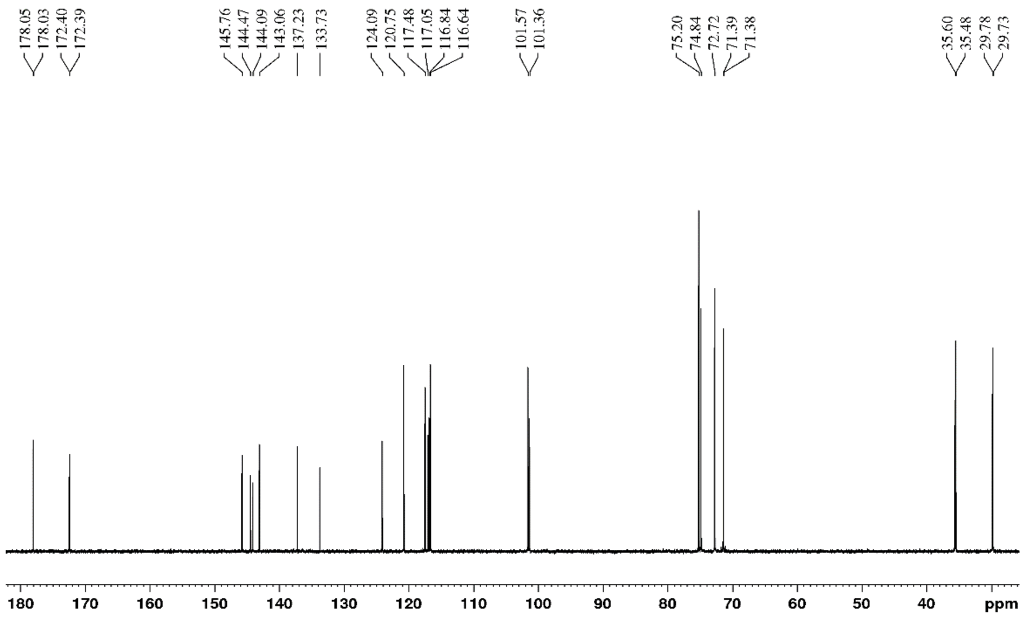

Figure S62. ^{13}C NMR spectrum of **DHPP-GlcA** (**6c** + **6c′**)
(150.93 MHz, D_2O, 30 °C)

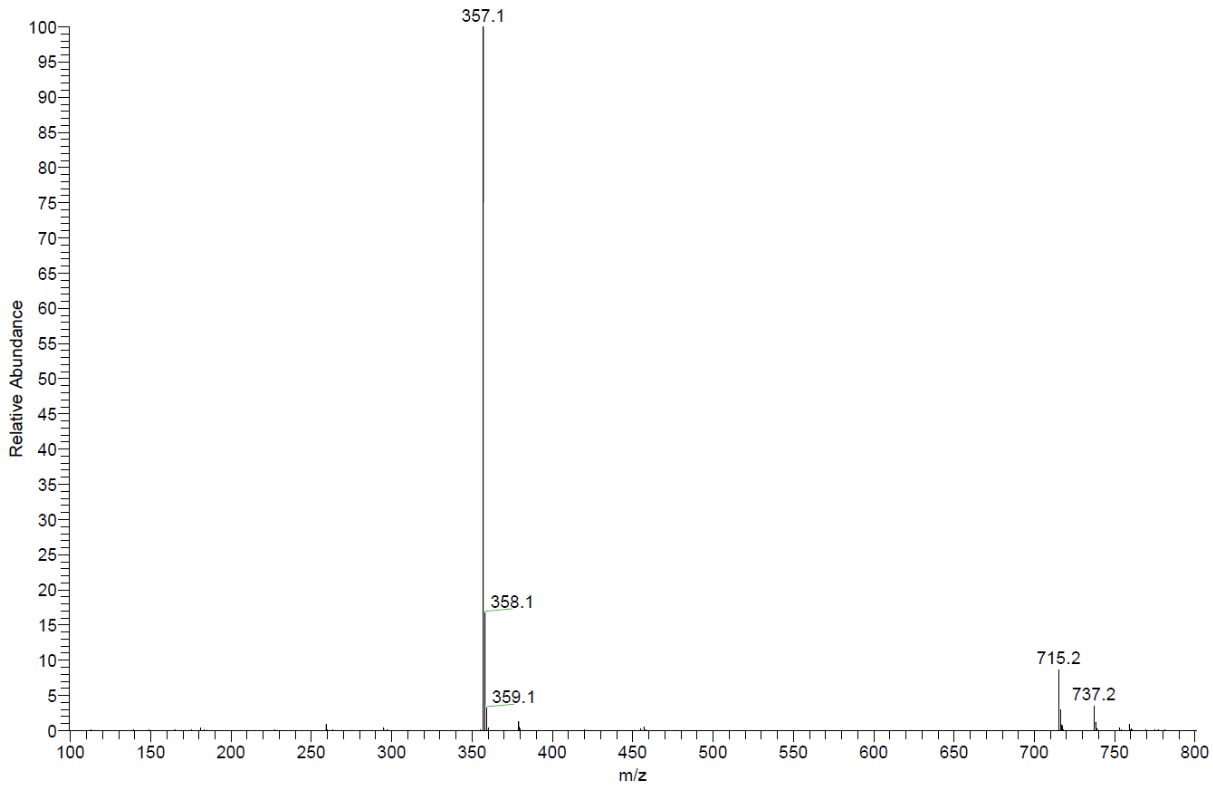

Figure S63. MS (ESI⁻) spectrum of **DHPP-GlcA** (**6c** + **6c'**)
([M-H]$^-$, *m/z* 357.1)

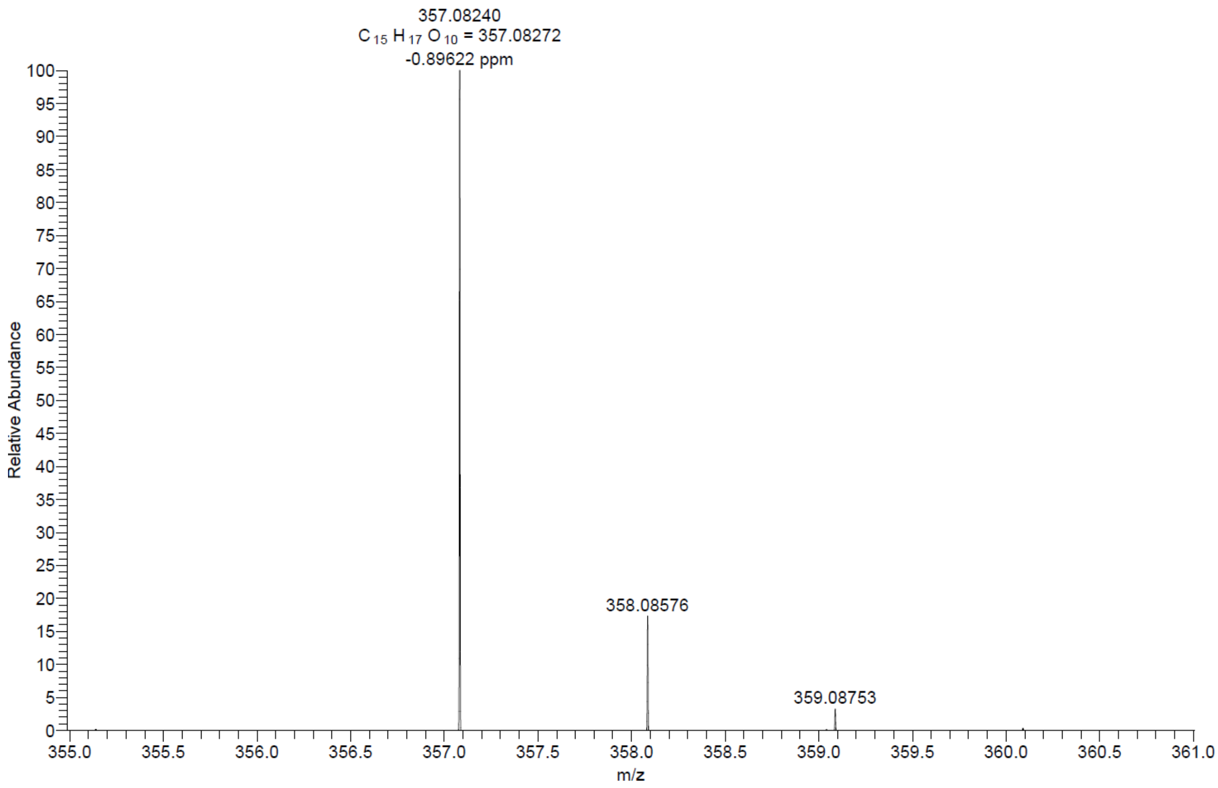

Figure S64. HRMS (ESI$^-$) spectrum of **DHPP-GlcA** (**6c** + **6c'**)
Calculated (for $C_{15}H_{17}O_{10}$) 357.08272, measured 357.08240 (-0.89 ppm).

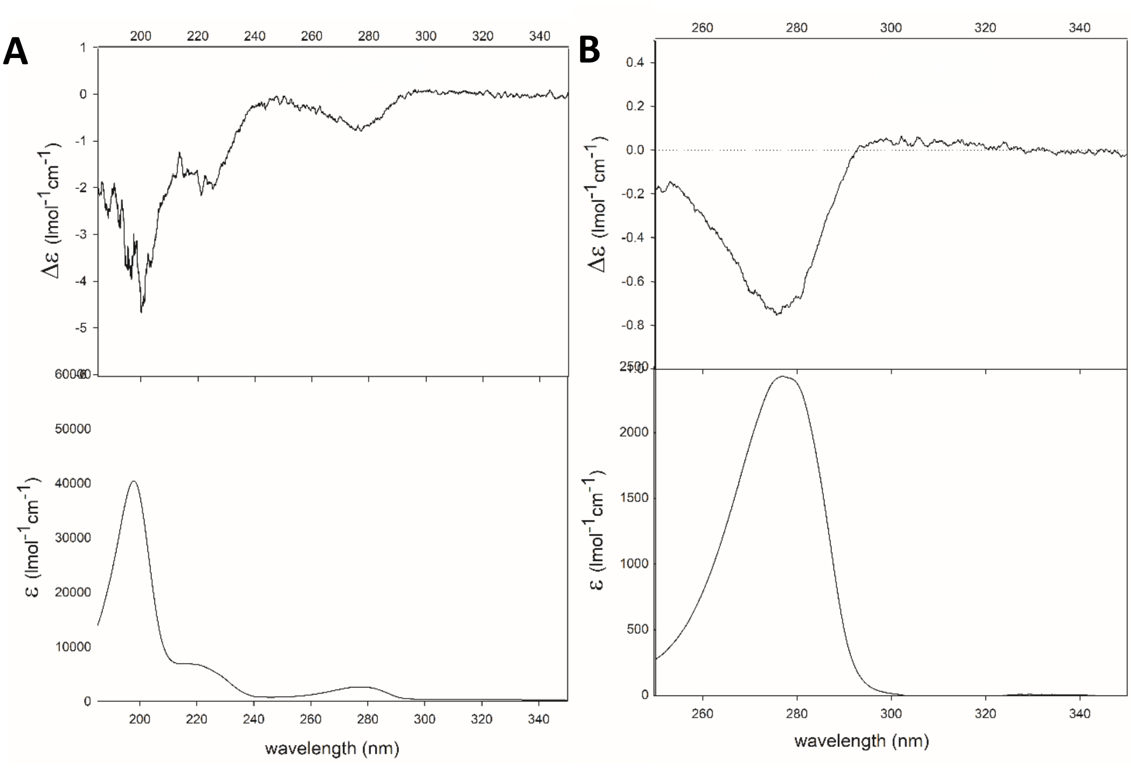

Figure S65. CD spectrum of **DHPP-GlcA** (**6c + 6c'**)
in the spectral range 190–350 nm (**A**) and in the near-UV range (250–280 nm, **B**)
